# Dogs were widely distributed across western Eurasia during the Palaeolithic

**DOI:** 10.1038/s41586-026-10170-x

**Published:** 2026-03-25

**Authors:** William A. Marsh, Lachie Scarsbrook, Eren Yüncü, Lizzie Hodgson, Audrey T. Lin, Maria De Iorio, Olaf Thalmann, Mark G. Thomas, Mahaut Goor, Anders Bergström, Angela Noseda, Sarieh Amiri, Fereidoun Biglari, Dušan Borić, Katia Bougiouri, Alberto Carmagnini, Maddalena Giannì, Tom Higham, Ophelie Lebrasseur, Anna Linderholm, Marcello A. Mannino, Caroline Middleton, Gökhan Mustafaoğlu, Angela Perri, Joris Peters, Mike Richards, Özlem Sarıtaş, Pontus Skoglund, Rhiannon E. Stevens, Chris Stringer, Kristina Tabbada, Helen M. Talbot, Laura G. Van der Sluis, Silvia M. Bello, Vesna Dimitrijevic, Louise Martin, Marjan Mashkour, Simon A. Parfitt, Sonja Vukovic, Selina Brace, Oliver E. Craig, Douglas Baird, Sophy Charlton, Greger Larson, Ian Barnes, Laurent A. F. Frantz

**Affiliations:** 1 Centre for Human Evolution Research, Natural History Museum, London, UK; 2 Chair of Animal Systems Genomics, Faculty of Veterinary Medicine, Ludwig-Maximilians-Universität, Munich, Germany; 3 Palaeogenomics and Bio-Archaeology Research Network, School of Archaeology, University of Oxford, Oxford, UK; 4 Department of Biological Sciences, Middle East Technical University, Ankara, Turkey; 5 BioArCh, Department of Archaeology, University of York, York, UK; 6 Richard Gilder Graduate School, American Museum of Natural History, New York, NY USA; 7 Department of Anthropology, National Museum of Natural History, Smithsonian Institution, Washington, DC USA; 8 Yong Loo Lin School of Medicine, National University of Singapore, Singapore, Singapore; 9 Institute for Human Development and Potential, A*STAR, Singapore, Singapore; 10 Department of Genetics, Evolution and Environment, University College London, London, UK; 11 School of Biological Sciences, University of East Anglia, Norwich, UK; 12 BioArchéologie, Interactions Sociétés Environnements, Muséum National d’Histoire Naturelle, Paris, France; 13 Histoire Naturelle des Humanités Préhistoriques, Muséum National d’Histoire Naturelle, Paris, France; 14 Bioarchaeology Laboratory, Central Laboratory, University of Tehran, Tehran, Iran; 15 University of Georgia, Athens, GA USA; 16 Paleolithic Department, National Museum of Iran, Tehran, Iran; 17 Dipartimento di Biologia Ambientale, SAPIENZA Università di Roma, Rome, Italy; 18 Section for Molecular Ecology and Evolution, Globe Institute, University of Copenhagen, Copenhagen, Denmark; 19 Department of Evolutionary Anthropology, Faculty of Life Sciences, University of Vienna, Vienna, Austria; 20 Human Evolution and Archaeological Sciences (HEAS) Network, University of Vienna, Vienna, Austria; 21 Centre de Recherche sur la Biodiversité et l’Environnement, Université de Toulouse, Toulouse INP, CNRS, IRD, CRBE, Toulouse, France; 22 Centre for Palaeogenetics, Stockholm University, Stockholm, Sweden; 23 Department of Geological Sciences, Stockholm University, Stockholm, Sweden; 24 Department of Archaeology and Heritage Studies, Aarhus University, Aarhus, Denmark; 25 Department of Archaeology, Classics and Egyptology, University of Liverpool, Liverpool, UK; 26 Haci Bayram Veli University, Ankara, Turkey; 27 Chronicle Heritage, Phoenix, AZ USA; 28 Institute of Palaeoanatomy, Domestication Research and the History of Veterinary Medicine, Ludwig-Maximilan-University Munich, Munich, Germany; 29 SNSB-State Collection of Palaeoanatomy Munich, Munich, Germany; 30 Simon Fraser University, Burnaby, British Columbia Canada; 31 Department of Prehistory, Division of Archaeology, Faculty of Arts and Sciences, Hitit University, Çorum, Turkey; 32 Ancient Genomics Laboratory, The Francis Crick Institute, London, UK; 33 Institute of Archaeology, University College London, London, UK; 34 Laboratory for Bioarchaeology, Department of Archaeology, Faculty of Philosophy, University of Belgrade, Belgrade, Serbia; 35 School of Biological and Behavioural Sciences, Queen Mary University of London, London, UK

**Keywords:** Archaeology, Population genetics, Coevolution, Genomics

## Abstract

Archaeological evidence suggests that dogs diverged from wolves during the Palaeolithic, more than 15,000 years ago^1–7^. The earliest unequivocal genetic evidence, however, is associated with dog remains from Mesolithic archaeological contexts approximately 10,900 years ago^8,9^. Here we generate both nuclear and mitochondrial genomes from canid remains at Pınarbaşı in Türkiye (15,800 years ago)^10^ and Gough’s Cave in the UK (14,300 years ago)^11^, as well as from dogs excavated from two Mesolithic sites in Serbia (Padina between 11,500–7,900 years ago and Vlasac 8,900 years ago)^12,13^. Our analyses indicate that a genetically homogeneous dog population was already widely distributed across Europe and Anatolia during the Late Upper Palaeolithic (by at least 14,300 years ago). This finding suggests that dogs were exchanged among genetically and culturally distinct western Eurasian Late Palaeolithic human populations, namely the Magdalenian, Epigravettian and Anatolian hunter-gatherers^10,14–16^. Last, we identify a major influx of eastern Eurasian dog ancestry during the Mesolithic, concomitant with the movement of eastern hunter-gatherer populations into Europe^14^, which led to the establishment of the primary ancestry characteristics that define European dog populations today.

## Abstract (web summary)

Analysis of nuclear and mitochondrial genomes from archaeological canid remains found across Europe and Anatolia shows that a genetically homogeneous dog population was already widely distributed across the region by 15,000 years ago.

## Main

Despite the application of both molecular and morphological approaches, the temporal and geographic origins of dog domestication remain unknown. Previous estimates have varied from 135,000 to 15,000 years ago^7^, which reflects both the large uncertainties associated with genetic-based dating methods and the difficulties in distinguishing skeletal remains of dogs and wolves on the basis of morphology. This is especially true in the earliest stages of domestication, during which there may have been an absence of detectable lineage-defining characteristics^17–19^.

Morphological analyses of archaeological canid remains have suggested that dogs were present across Eurasia during the Upper Palaeolithic^1–6^ (35,000–15,000 years ago). Stable dietary isotopes (δ^13^C and δ^15^N) have also been used to tentatively distinguish dogs from wolves in Palaeolithic contexts^20^. Without corroborating nuclear genomic data, however, definitive identification of these remains as dogs has been challenging. For instance, although Upper Palaeolithic remains from Europe (for example, approximately 34,000 years calibrated before present (cal bp) from Goyet in Belgium^1^; approximately 28,500 years cal bp from Předmostí in Czechia^2^) and Russia (for example, approximately 33,000 years cal bp from Razboinichya Cave in the Altai Mountains^3^; approximately 17,600 years cal bp from Eliseevichi in western Russia^5^) were initially identified as early dogs using morphological approaches, nuclear genomes generated from these individuals have shown they belonged to now-extinct wolf populations^21,22^. The earliest definitive dogs, on the basis of nuclear genetic data, have been identified at the Mesolithic site of Veretye (Karelia, Russia), dated to approximately 10,900 years cal bp (ref. ^8^).

Although Palaeolithic dogs are yet to be unequivocally identified, several candidates have been suggested. An approximately 14,300 years cal bp canid mandible from the Late Upper Palaeolithic site of Bonn-Oberkassel (Germany), for example, shows dog-like morphology, was co-interred alongside a dual human burial and shows pathologies that would have been lethal if not for prolonged human care^23^. Claims for Palaeolithic dogs, based on either morphological or biomolecular analyses, or both, have also been made for material from sites across continental Europe including Kesserloch (Switzerland^24,25^), Erralla (Spain^26^), Grotta Paglicci (Italy^27^) and Le Morin (France^28^). Other candidates for early dogs include potential burials of juvenile canids at the Epipalaeolithic site of Pınarbaşı (16,100–12,900 years cal bp) on the Central Anatolian Plateau in Türkiye^10^ (Supplementary Fig. 1) and canid remains from a Late Upper Palaeolithic Magdalenian horizon (15,100–14,200 years cal bp) at Gough’s Cave in the UK^29,30^ (Supplementary Fig. 2). Remains at both of these sites show postmortem treatment that mirrors the human remains^15,29^.

**Fig. 1:**
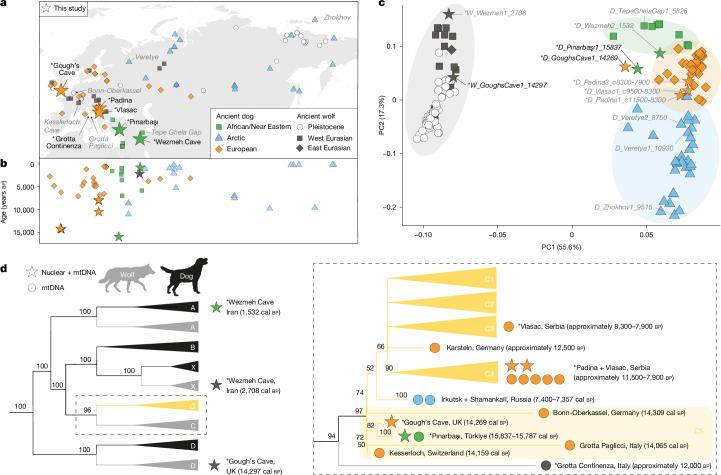
Dogs were widespread in Palaeolithic Europe and Anatolia. **a**, Location of the published ancient African and/or Near Eastern (green), European (orange) and Arctic (blue) dog, as well as Pleistocene (white) and Holocene (dark grey) Eurasian wolf genome data analysed in this study (*n* = 139), with unpublished canid specimens shown as stars (*n* = 8). Sites from which new genomic data (mitochondrial or nuclear) were generated are labelled in black text. Frequently mentioned sites from which data were already available are labelled in grey text. **b**, Temporal distribution of ancient Eurasian dog and unpublished wolf nuclear genomes in **a**, based on either direct radiocarbon dates, or securely dated contexts (Supplementary Table 4). **c**, Principal component (PC) analysis of ancient dogs (*n* = 73) and wolves (*n* = 66). Frequently mentioned samples are labelled in either black (Palaeolithic) or grey (Mesolithic-Byzantine). **d**, Simplified phylogeny based on mitogenomes (see Supplementary Fig. 6 for the complete tree) of 202 dogs (black) and 17 wolves (grey). The C haplogroup is shown in more detail (right panel) to highlight the position of C5 dogs (which included Gough’s Cave and Pınarbaşı) as a sister clade to all other C dogs, as well as the position of the Grotta Continenza wolf as a sister lineage to the whole C (including C5). Polytomies represent nodes for which bootstrap support values were less than 50. Across all figures, an asterisk (*) indicates data generated in this study.

Here, to test the hypothesis that dogs were present in western Eurasia during the Palaeolithic, we directly dated and generated nuclear genome data (median depth 1.5×, 0.1–3.0×) from canid remains from Gough’s Cave (UK, *n* = 2), Pınarbaşı (Türkiye, *n* = 1) and a suspected Palaeolithic deposit from Wezmeh Cave (Iran, *n* = 2). We also generated low-coverage genome data (median depth 0.11×; 0.07–0.17×) from morphometrically identified dogs from Mesolithic contexts in Serbia (Padina; *n* = 2; Vlasac; *n* = 1, refs. ^12,13^; Fig. 1a,b, Supplementary Tables 1 and 2 and Supplementary Fig. 3). These data were analysed alongside previously published ancient dog (*n* = 68) and wolf (*n* = 71) genomes spanning the past 100,000 years (Supplementary Table 1), as well as 276 modern dog, wolf and outgroup canid genomes (which are a representative subset of the 1,700 genomes included in the NHGRI Dog Genome Project Database; Supplementary Table 3, ref. ^31^).

In addition, we generated low-coverage sequencing data (less than 0.001×) and used in-solution capture to obtain high-coverage mitochondrial genomes (median depth 15.7×, 5.0–72.3×) from canid remains from Pınarbaşı (*n* = 3), Padina (*n* = 2), Vlasac (*n* = 1) and a Late Upper Palaeolithic canid from Grotta Continenza (Italy, *n* = 1; Supplementary Tables 4 and 5). Finally, to assess the degree to which the dietary isotopes derived from canid remains overlapped with those from Palaeolithic humans (for example, ref. ^32^), we performed carbon (δ^13^C) and nitrogen (δ^15^N) compound-specific stable isotope analysis of individual collagen amino acids of human and canid remains at Gough’s Cave (*n* = 6) and Pınarbaşı (*n* = 6; Supplementary Table 6).

## Dogs were widespread during the Palaeolithic

To determine whether these newly sequenced canids were genetically closer to modern and ancient dogs or wolves, we first performed principal component analysis (Fig. 1c and Supplementary Fig. 4) and unsupervised ADMIXTURE (*K* = 2, which differentiates dogs and wolves; Supplementary Fig. 5) analyses based on nuclear genomic data. Of the eight newly sequenced individuals, six clustered with modern and ancient dogs, including an approximately 15,800 year cal bp individual from Pınarbaşı (1.3×; 15,915–15,669 years cal bp), an approximately 14,300 year cal bp individual from Gough’s Cave (1.7×; 14,793–14,090 years cal bp), all three Mesolithic Serbian individuals (Padina (0.1× and 0.2×) and Vlasac (0.1×); 11,500–7,900 years bp), and an approximately 1,532 year cal bp individual from Wezmeh Cave in Iran (0.1×; 1,574–1,419 years cal bp).

**Extended Data Fig. 1:**
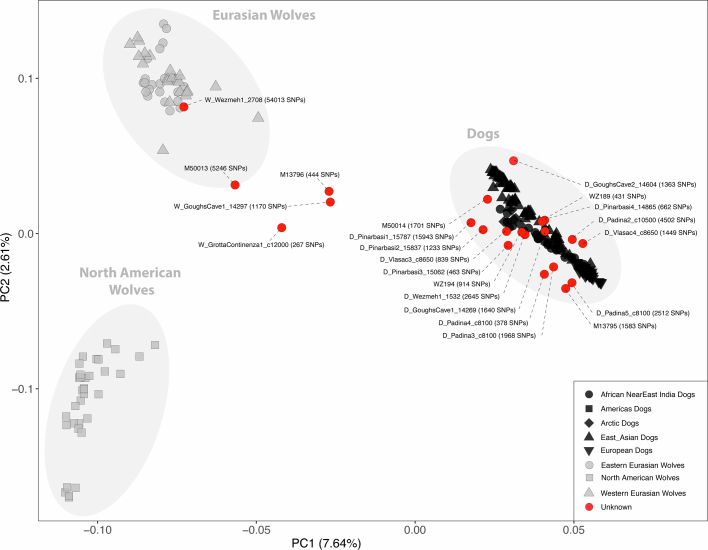
Taxonomic identification of canids using CanID. Projection-based Principal Component (PC) analysis performed as part of the CanID pipeline using ultra-low coverage sequencing data from novel ancient samples (n = 21)^34^. Taxonomic identifications (i.e., dog or wolf; Supplementary Table 3) determined based on linear discriminant analysis across the first 10 PCs were validated through visual inspection.

We also identified an approximately 14,300 year cal bp wolf from the same context at Gough’s Cave (0.2×; 14,808–14,091 years cal bp) and an approximately 2,700 year cal bp wolf from Wezmeh Cave in Iran (3.0×; 2,745–2,542 years cal bp). The discrepancy between contextual and direct age estimates for canids from Wezmeh Cave reflects recent anthropogenic disturbance of the Palaeolithic horizon^33^. All taxonomic classifications were consistent with assignments generated using a pipeline that distinguishes dogs and wolves given ultra-low-coverage sequencing (screening) data^34^. Using the screening data (a few million reads per library), this pipeline identified a further 13 dogs and two wolves at these sites, as well as a wolf from the Palaeolithic site of Grotta Continenza (Extended Data Fig. 1 and Supplementary Table 4).

**Extended Data Fig. 2:**
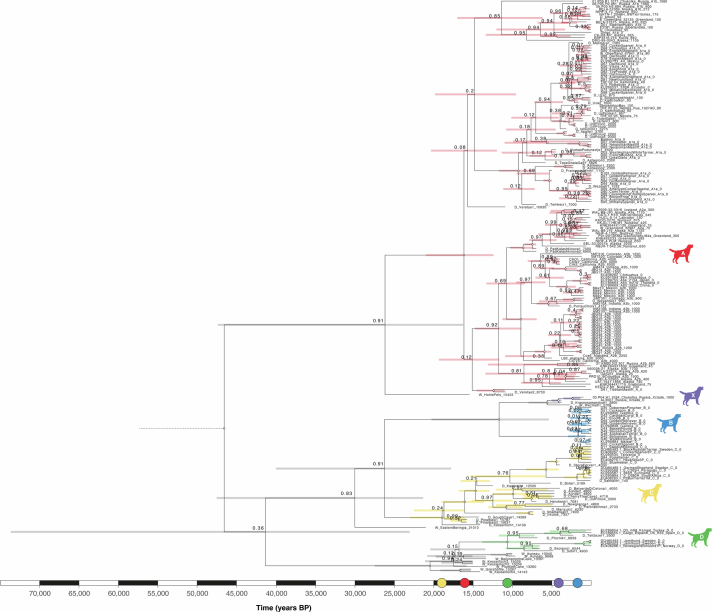
Estimating the most recent common ancestor of Haplogroup C. Bayesian time-calibrated phylogeny constructed from 240 canid mitochondrial genomes using BEAST2^83^. Tip heights greater than 0 represent calibrated radiocarbon ages of ancient samples. Node bars represent 95% highest posterior density for divergence estimates, and values represent posterior probabilities (with white circles indicating 100% support). Coloured circles indicate the mean most recent common ancestor of major dog haplogroups: A (red), B (blue), C (yellow), D (green) and X (purple). To ensure our divergence time estimates were robust, we excluded samples with no direct radiocarbon age (e.g., Mesolithic Serbian dogs), and a depth of coverage of <10x (e.g., Palaeolithic Gough’s Cave wolf; see Supplementary Table 4).

The definitive nuclear genome-based identification of dogs from Gough’s Cave and Pınarbaşı enabled us to evaluate newly generated and publicly available mitochondrial DNA (mtDNA) data to assess the status of other Palaeolithic canids. Dog mtDNA can be divided into five monophyletic haplogroups (A–D and X), each of which are sister to extinct and extant wolf lineages (Fig. 1d and Extended Data Fig. 2). Most of the suspected European Palaeolithic dogs for which mtDNA has previously been generated^20,24,27^ branch off just outside the diversity of C haplogroup dogs in previous analyses^35^. This phylogenetic placement, outside the diversity of modern and ancient dogs, yet closer to dogs than wolves, has made it difficult to assess their domestication status.

**Fig. 2:**
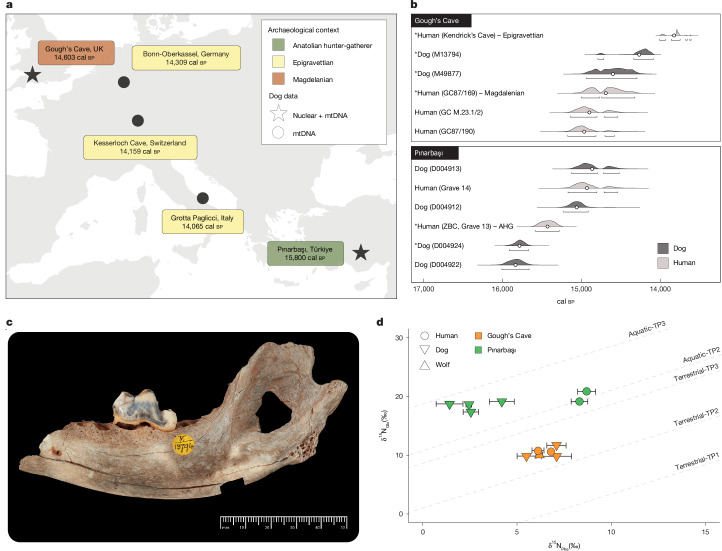
Close human–dog associations during the Palaeolithic. **a**, Earliest directly dated dogs from Late Upper Palaeolithic archaeological contexts across western Eurasia based on both mitochondrial (circle) and nuclear (star) DNA. **b**, Calibrated radiocarbon dates of canid and human remains at both Gough’s Cave and Pınarbaşı. The genetic ancestry of human remains, Anatolian hunter-gatherer (AHG), Epigravettian-associated and Magdalenian-associated, are indicated for those with publicly available nuclear genomes^36,37^. Dates were calibrated using OxCal v.4.4 and the IntCal20 calibration curve, with mean age (circles) and 95% confidence intervals reported^69,70^. **c**, The Gough’s Cave dog (NHMUK PV M 13794a) mandible in lateral view. Anthropic piercing of the masseteric fossa can be seen. **d**, δ^15^N values of glutamic acid (Glx) and phenylalanine (Phe) of bone collagen from human and canid bones. To infer trophic positions (TP), measured values are compared against isotopic thresholds (dashed lines) derived from trophic discrimination factors (TDF) and beta values, calibrated using terrestrial (vascular) and aquatic (non-vascular) autotrophs^71^. The interpretation of the Pınarbaşı neonatal canids assumes a minimal offset in _Phe_ and _Glx_ δ^15^N values between mothers and their foetal offspring^54^. Photograph in **c** reproduced with permission from The Trustees of the Natural History Museum, London.

We constructed a maximum-likelihood phylogeny using 220 mitogenomes (including 202 dogs and 17 wolves) representative of all known dog haplogroups, and a coyote as an outgroup (Fig. 1d and Supplementary Fig. 6). The dogs from Gough’s Cave and Pınarbaşı cluster together with other suspected European Palaeolithic dogs, sister to C haplogroup dogs, a haplogroup that we termed C5 (Fig. 1d). This clade also includes canids from Epigravettian sites in Germany (Bonn-Oberkassel, 14,805–14,088 years cal bp, ref. ^24^), Switzerland (Kesslerloch, 14,318–14,039 years cal bp, ref. ^20^) and Italy (Grotta Paglicci, 14,310–13,859 years cal bp, ref. ^27^; Fig. 2a). The Grotta Continenza wolf formed a sister lineage to the diversity of all C haplogroup dogs including C5.

The placement of the Bonn-Oberkassel, Kesslerloch and Grotta Paglicci individuals within a dog-specific mitochondrial clade suggests that they also carry dog nuclear ancestry. This association between dog nuclear ancestry and the C5 mtDNA clade therefore indicates that dogs were widely distributed across western Eurasia during the Late Upper Palaeolithic.

## Genetically similar Palaeolithic dogs

**Extended Data Fig. 3:**
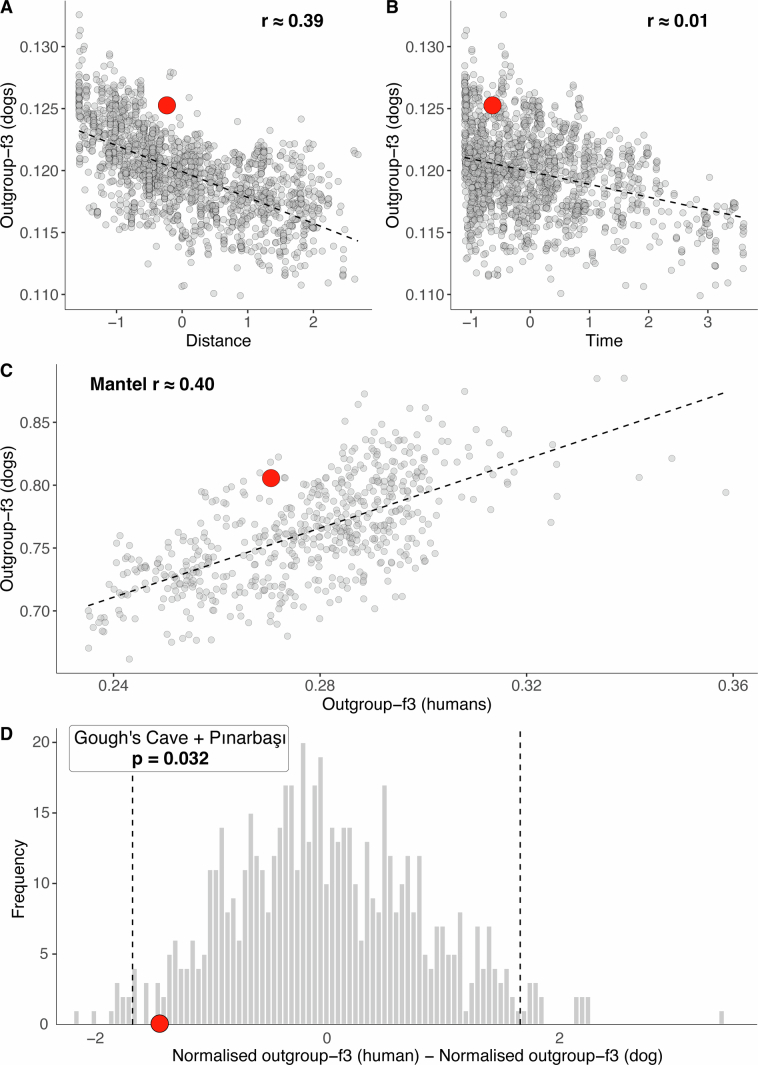
Investigating shared patterns of drift between dogs and humans. Correlation between outgroup-f3 and both (**A**) geographic distance and (**B**) time. Outgroup-f3 statistics of the form: f3(Coyote, X, Y), where X and Y represent all possible pairwise comparisons of ancient dogs. Geographic distance represents the great-circles (Haversine) distance between the archaeological sites under comparison. Time represents the age difference between the samples based on either contextual estimates or direct radiocarbon dating (Supplementary Table 1). We excluded all individuals with <0.01x coverage (n = 63). The red circle indicates the comparison between Gough’s Cave and Pınarbaşı, which was not a significant outlier when both space and time were accounted for. (**C**) Pairwise outgroup-f3 for sites (n = 35) where humans (x-axis) and dogs (y-axis) were found in the same context. (**D**) Histogram of the differences between normalized human and dog outgroup-f3 statistics following Extended Data Fig. 3c. Values for each comparison were normalized based on median absolute deviation (MAD). Dashed lines representing two standard deviations from the mean observed difference. Positive values indicate humans are more similar than expected, negative values dogs more similar than expected.

**Extended Data Fig. 4:**
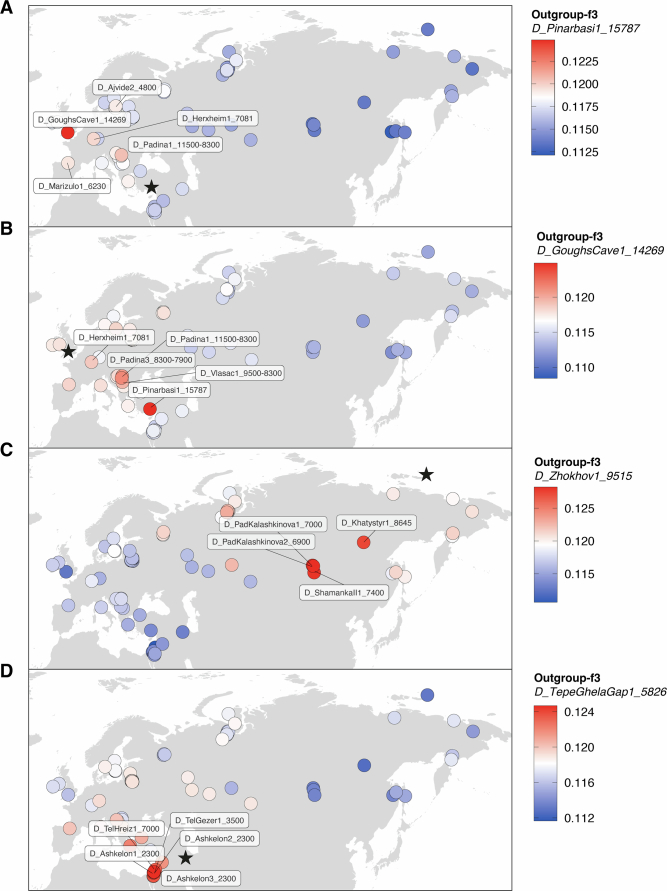
Genetic affinities of ancient dogs to major dog ancestries. (**A**) Heat maps showing shared drift (outgroup-f3) of ancient dog to major dog ancestries: (**A**) Pınarbaşı (Palaeolithic), (**B**) Gough’s Cave (Palaeolithic), (**C**) Zhokhov (Arctic), and (**D**) Tepe Ghela Gap (Near East); and all other ancient dogs (X), calculated using the following outgroup-f3 test setup: f3(Coyote, X, Pınarbaşı/Gough’s Cave/Zhokhov/Tepe Ghela Gap). Stars indicate the location of the archaeological sites that contain individuals representative of each of the major dog ancestries, and the labelled sites represent the five highest outgroup-f3 values for each comparison.

**Extended Data Fig. 5:**
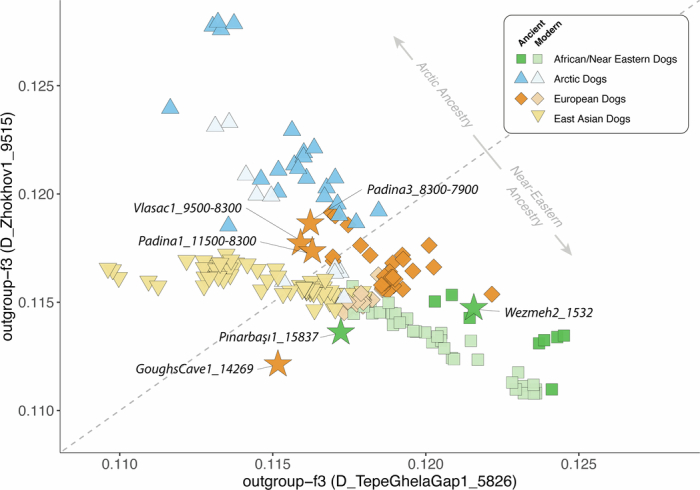
The affinities of ancient dogs to Near Eastern and Arctic dog lineages. Levels of shared drift (outgroup-f3) between ancient or modern dogs and an ancient Near Eastern dog (Iran_5826; x-axis) and an Arctic dog (Zhokhov_9515; y-axis). Samples are coloured based on both shared ancestry and geographic proximity: African/Near Eastern (green squares), European (orange diamonds), Arctic (blue triangles), and East Asia (yellow triangles).

To assess the degree of similarity between the Late Upper Palaeolithic dogs, we first inferred a Bayesian time-calibrated mtDNA phylogeny. The estimated time to the most recent common ancestor of the Gough’s Cave and Pınarbaşı individuals was approximately 16,900 years ago (95% highest posterior density, 18,569–15,860 years ago), less than 3,000 years before the death of the Pınarbaşı dog (Extended Data Fig. 2). Furthermore, pairwise distance calculations, outgroup-*f*_3_ (Extended Data Figs. 3–5) and kinship (*θ*_Gough’sCave/Pınarbaşı_ 0.010, *θ*_median_ 0.005; Supplementary Figs. 7 and 8) analysis of autosomal data indicate that dogs from Gough’s Cave and Pınarbaşı are more genetically similar to each other than to any other dog. Combined, our mtDNA and nuclear results indicate the Pınarbaşı and Gough’s Cave individuals at the eastern and western extremes of the known distribution of Palaeolithic dogs were genetically very similar, and were members of a population that expanded across western Eurasia between 18,500 years and 14,000 years ago.

Of the five Palaeolithic dogs (Fig. 2a), each is associated with one of three genetically and culturally distinct human hunter-gatherer populations found across Europe and Anatolia in the Late Upper Palaeolithic: the Magdalenian (Gough’s Cave^29,30^), the Epigravettian (Bonn-Oberkassel, Kesslerloch and Grotta Paglicci^20,24,27^) and Anatolian hunter-gatherers (Pınarbaşı^10^). The spread of these dogs across the region is likely to have been linked with the migration, dispersal and interaction of humans associated with these Palaeolithic cultures. In fact, although most Palaeolithic dogs in this study are associated with human populations of Epigravettian-associated genetic ancestry, including Anatolian hunter-gatherers at Pınarbaşı^36^, the dog from Gough’s Cave was recovered from a depositional context alongside humans with Magdalenian-associated ancestry^37^.

The presence of genetically similar dogs across sites with genetically differentiated human populations in the Palaeolithic suggests that dogs and humans have had different population histories. To assess this, we first established the degree to which humans and dog ancestries from the same archaeological contexts, across 35 sites that spanned the Late Upper Palaeolithic to the Medieval period, were correlated using outgroup-*f*_3_ statistics as a genetic similarity measure (Extended Data Fig. 3c and Supplementary Table 7). After correcting for time and spatial autocorrelation (Methods), we found a strong positive correlation (Mantel *r* ≈ 0.40, *P* < 0.0001) between human–human and dog–dog outgroup-*f*_3_ values, suggesting shared evolutionary histories between humans and dogs that cannot be explained by shared spatial or temporal structure alone.

In our comparison of the differences between human–human and dog–dog outgroup-*f*_3_ at the same site, we observed that the dogs from the Palaeolithic sites of Pınarbaşı and Gough’s Cave fell within the tail of the distribution. This indicates that there existed a greater degree of genetic similarity between the dogs than among the associated humans at the same sites (Extended Data Fig. 3d) and provides further evidence that a relatively homogeneous dog population spread between genetically and culturally distinct human populations across Europe and Anatolia in the Late Upper Palaeolithic.

One plausible scenario to explain the discrepancy between human and dog ancestry in the Palaeolithic is that the dispersal of dogs was coupled with the spread of Epigravettian-associated ancestry and material culture roughly 16,000 years ago, which, after a period of interaction, eventually replaced the previously predominant Magdalenian culture and ancestry across northern Europe^14,16,37^. This timeframe aligns well with our mtDNA-based most recent common ancestor estimate for the Gough’s Cave and Pınarbaşı dogs (95% highest posterior density, 18,569–15,860 years ago), which provides an estimated upper bound for the divergence of their ancestral populations (which must have taken place after approximately 18,500 years ago), and the youngest radiocarbon date (Gough’s Cave dog, 14,808–14,091 years cal bp; Supplementary Table 4), which provide a minimum age by which their ancestral population must have already diverged. Crucially, the timeframe for the spread of dogs in western Eurasia (between 18,500–14,000 years ago) postdates the earlier divergence of Magdalenian and Epigravettian human populations, which most probably occurred during or before the Last Glacial Maximum (approximately 24,000–21,000 years ago^14,38^). The spread of Palaeolithic dogs across western Eurasia, therefore, most probably occurred after the divergence of these two distinct human populations, possibly during the spread of Eppigravettian-associated ancestry and material culture roughly 16,000 years ago.

The Gough’s Cave dog, however, dates to the Late Magdalenian (from 15,000 years ago; Fig. 2b), a period for which there is at present no evidence of humans carrying Epigravettian-associated ancestry in the UK^37^. Yet, this period is characterized by transitions in lithic technology (for example, *Federmessergroupen*) that are associated with people carrying Epigravettian-related ancestry on the European continent (for example, at Bonn-Oberkassel)^39–42^, whereas later individuals from the UK (for example, Kendrick’s Cave, 13,780–13,354 years cal bp) carry Epigravettian-associated ancestry^14,37^. Further, the putative presence of dogs (based on short mtDNA fragments) has also been suggested at another Magdalenian context, in Spain (Erralla Cave, 17,410–17,096 years cal bp, ref. ^26^) and, unlike at Gough’s Cave, these remains postdate the earliest evidence of Epigravettian ancestry in the region by more than 1,000 years (El Mirón, approximately 18,700 years cal bp, refs. ^43,44^).

Combined, our results are consistent with a dispersal of dogs across Late Palaeolithic Europe alongside the expansion of Epigravettian-associated ancestry and material culture approximately 16,000 years ago. Under this scenario, people carrying Magdalenian-associated ancestry in the UK, and perhaps in Spain, acquired dogs through interactions with Epigravettians. These interactions did not leave any signal of Epigravettian ancestry in the Magdalenian humans at Gough’s Cave^37^, implying that the exchange of dogs between Palaeolithic human groups was not always accompanied by detectable gene flow within the human populations. However, an alternative scenario that involves dogs being exchanged through networks that are not associated with the Epigravettian expansion cannot at present be ruled out due to the paucity of Palaeolithic dog remains.

## Close Palaeolithic human–dog association

The discovery of dogs within Magdalenian, Epigravetian and Anatolian hunter-gatherer contexts across western Eurasia indicates dogs were integrated with culturally, geographically and genetically distinct human groups. At Gough’s Cave, human remains in a Magdalenian context show postmortem anthropic modification including the shaping of skulls into skull-cups and the engraving of human remains^15,45^. This is indicative of funerary cannibalism, a behaviour identified at Magdalenian sites across Europe^15,16^. Similar postmortem anthropic modification is evident on the dog remains at Gough’s Cave, most notably a perforation on the masseteric fossa (Fig. 2c and Supplementary Fig. 2), indicating the shared treatment practices of humans and dogs postmortem. Similar shared postmortem treatment of dog and human remains is also evident at Pınarbaşı, where neonatal and juvenile dogs were buried in the same area of the site as contemporaneous human burials^10^ (Fig. 2b). These shared patterns of postmortem treatment extend the symbolic treatment of dogs identified during the Mesolithic to earlier Palaeolithic hunter-gatherers^46,47^.

To investigate whether similar close associations were also evident during the lifetime of the dogs, we evaluated the degree of dietary similarity between canids and humans at both Gough’s Cave and Pınarbaşı through the measurement of bulk and amino acid stable isotopes (δ^13^C and δ^15^N) in bone collagen^48^ (Fig. 2d, Supplementary Fig. 9 and Supplementary Table 6).

At Gough’s Cave, the dogs and humans have comparable bulk isotope and amino acid δ^15^N values, and the δ^15^N_Glx-Phe_ proxy for trophic position (Fig. 2d) indicates a similar degree of omnivory^49,50^. The Gough’s Cave wolf occupied a similar trophic position to both these species (Fig. 2d). Niche partitioning between dogs and wolves (for example, ref. ^51^) may have, therefore, been less pronounced in Late Upper Palaeolithic Europe compared with later periods, notably after the emergence of starch-rich diets in the Neolithic^52^ (Supplementary Fig. 10). We note, however, that the isotopic approach lacks the resolution to definitively discriminate between wild and domestic canids, as different diets may result in identical isotopic signatures^53^. Alternatively, the dietary overlap between wolves and humans at Gough’s Cave could also indicate close association, although this remains tentative given the lack of comparable isotopic data from wolves outside anthropogenic contexts in the Late Upper Palaeolithic.

At Pınarbaşı, although perinatal dogs (and by proxy their mothers) had different isotopic signatures to the humans (Fig. 2d), the δ^15^N_Glx-Phe_ values of both species were elevated relative to Gough’s Cave. Notwithstanding uncertainties in the mother–offspring isotopic offset^54^, these data indicate an aquatic dietary component (Fig. 2d and Supplementary Table 6). The remains of small freshwater fish, probably net-caught, are common in the human-occupied layers at Pınarbaşı^10^, suggesting that dogs were either being directly or indirectly provisioned by humans.

## Genetic ancestry of Palaeolithic dogs

Dogs can be broadly separated into two lineages: an eastern lineage, found in Arctic, East Asian and precontact American dogs (which mostly disappeared after CE 1492, ref. ^55^), and a western lineage that encompasses dogs from Europe and the Near East^56^. The presence of both eastern and western ancestries in Karelian Mesolithic dogs approximately 10,900 years ago^8^ implies that the two ancestries diverged during the Palaeolithic. To establish the ancestry of Palaeolithic and Mesolithic European dogs, we calculated shared drift with representatives of eastern (an approximately 9,500 years cal bp Siberian hunter-gatherer dog from Zhokhov Island^57^) and western (an approximately 5,800 years cal bp dog from the Neolithic Iranian site of Tepe Ghela Gap^58^) dog lineages using outgroup-*f*_3_ statistics of the form *f*_3_(Coyote, *D*_TepeGhelaGap1_5826/*D*_Zhokhov1_9515, *X*).

Palaeolithic dogs from Europe and Anatolia share more drift with the western Eurasian lineage (Extended Data Figs. 4 and 5), which is also apparent from principal component analysis (Fig. 1c and Supplementary Fig. 4), and *D* statistics of the form *D*(Coyote, *X*, *D*_Zhokhov1_9515, *D*_TepeGhelaGap1_5826) (Supplementary Fig. 11). These results indicate that Palaeolithic dogs form part of the western Eurasian dog lineage, which pushes the divergence of eastern–western dog populations to at least 15,800 years ago.

## Wolf–dog admixture in the ancient Near East

**Extended Data Fig. 6:**
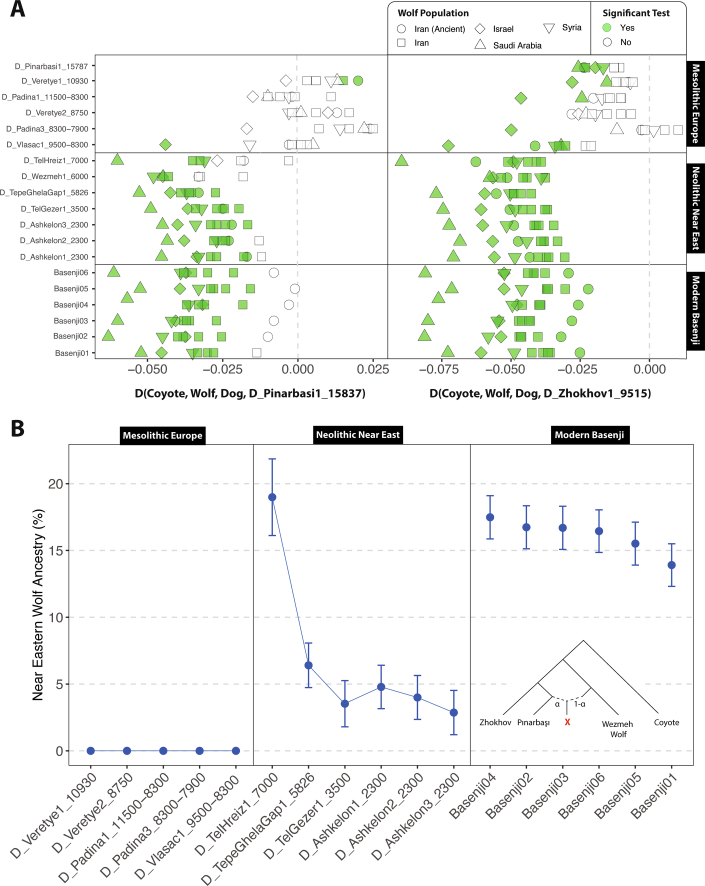
Evidence of local wolf introgression into Near Eastern dogs. (**A**) Excess of shared derived alleles between Mesolithic European and Neolithic to recent Near Eastern dogs (Y) and Near Eastern wolves (X) using D-statistics of the form: D(Coyote, X; Y, D_Pinarbasi1_15787). (**B**) F4-ratio showing proportion of Near Eastern wolf ancestry (alpha ± standard error) in Near Eastern dogs using the ancient Iranian wolf (W_Wezmeh1_2708) as a source. Alpha values for all Mesolithic European dogs (i.e. Veretye, Padina, Vlasac) exceeded 1, so the Near Eastern wolf ancestry proportion was set to 0.

A recent genomic study^22^ indicated that both modern and ancient western Eurasian dogs possess genetic ancestry similar to present-day wolves in Syria. To assess whether newly sequenced western Eurasian Palaeolithic and Mesolithic dogs also possess Near Eastern wolf ancestry, we performed *D* statistics of the form *D*(Coyote, Near Eastern Wolf, Early Western Eurasian Dogs, *D*_Zhokhov1_9515). We used a newly sequenced genome (3×) from an approximately 2,700-year-old wolf from Iran (Wezmeh Cave; Fig. 1a) as a proxy for Near Eastern wolf ancestry, because some present-day Near Eastern wolf populations (for example, Israel and Saudi Arabia) probably acquired dog ancestry through hybridization more recently (Extended Data Fig. 6a).

We identified excess allele sharing between ancient Near Eastern dogs (including the Palaeolithic dog from Pınarbaşı) and the Wezmeh wolf, consistent with gene flow between dogs and the local Near Eastern wolf population as far back as 15,800 years ago (Extended Data Fig. 6a and Supplementary Fig. 12). We found no evidence of excess allele sharing with Mesolithic dogs from Veretye (Karelia, north-west Russia; approximately 10,900 years cal bp) or Padina (Serbia; 11,500–7,900 years bp). However, tests were significant for a Mesolithic dog from Vlasac (Serbia; 9,500–8,300 years bp). These results indicate that Near Eastern wolf ancestry was variable in dogs across western Eurasia during the Palaeolithic and Mesolithic.

In the Near East, dogs from the Neolithic and onwards show excess allele sharing with Near Eastern wolves when compared with contemporary European dogs (Extended Data Fig. 6a). *D* statistics of the form *D*(Coyote, Near Eastern Wolf, *X*, *D*_Pinarbasi1_15787) were significantly negative in most cases, suggesting that these Near East dogs possess more Near Eastern wolf ancestry than the Pınarbaşı dog (Supplementary Fig. 13). To quantify the extent of excess Near Eastern wolf ancestry in these Neolithic and post-Neolithic populations, we computed *F*_4_-ratios (Extended Data Fig. 6b) and performed supervised ADMIXTURE analysis (Supplementary Fig. 14). These analyses identified the highest levels of wolf ancestry (*F*_4_ ratio 19.0%, ADMIXTURE 13.5%) in a 7,000-year-old dog from Tel Hreiz (Israel; Supplementary Fig. 14). This component declined over the subsequent millennia to less than 5% (*F*_4_ ratio 2.9–4.8%, ADMIXTURE 3.2–4.8%) in 2,300-year-old dogs from Ashkelon (Israel).

By contrast, high levels of wolf ancestry are maintained in modern Basenjis (*F*_4_ ratio 13.9–17.4%; Extended Data Fig. 6b), possibly due to the isolation of this dog population in sub-Saharan Africa until the colonial period. A previous study suggested that dogs in Africa experienced gene flow with endemic African canids^59^. It is possible, therefore, that Near Eastern wolf ancestry identified in Basenjis may derive from gene flow with these endemic canids after dogs dispersed into Africa.

**Fig. 3:**
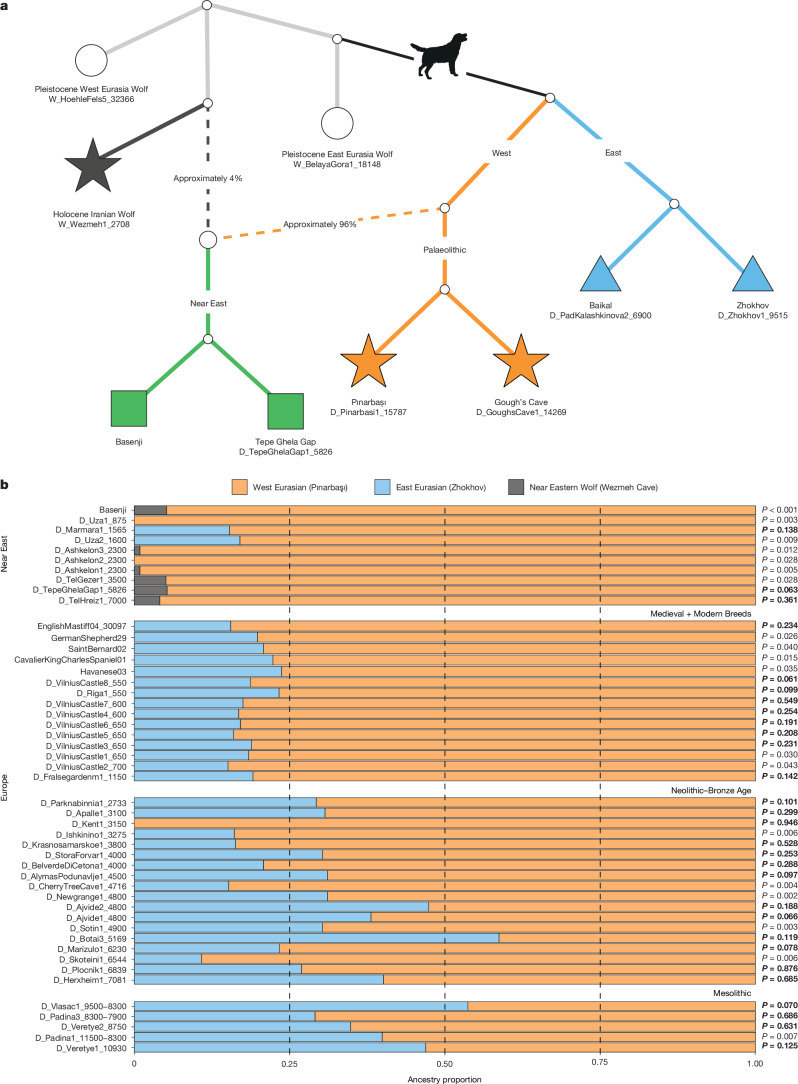
Ancestry of European dogs from Mesolithic to today. **a**, Admixture graph generated using AdmixtureBayes, allowing for a single admixture event (*k* = 1), which showed the highest posterior support (0.77) of all models (see Supplementary Fig. 16 for further admixture events). Mean ancestry proportions were estimated in AdmixtureBayes using coyotes as an outgroup. **b**, Proportion of Palaeolithic western Eurasian (orange; *D*_Pinarbasi1_15787), eastern Eurasian (blue; *D*_Zhokhov1_9515) and Near Eastern wolf (grey; *W*_Wezmeh1_2708) ancestries in modern and ancient Near Eastern (top) and European (bottom) dogs, estimated using qpAdm. Models were not rejected if their *P* value exceeded a threshold of 0.01 (those exceeding 0.05 are shown in bold). European dogs are separated into key time periods (Supplementary Fig. 21). The Gough’s Cave dog (*D*_GoughsCave1_14269), which was modelled as a mixture of Palaeolithic western Eurasian (82.4%) and Near Eastern wolf (17.6%) ancestries (*P* < 0.01), was excluded as the source of basal ancestry could not be identified (Supplementary Information).

**Extended Data Fig. 7:**
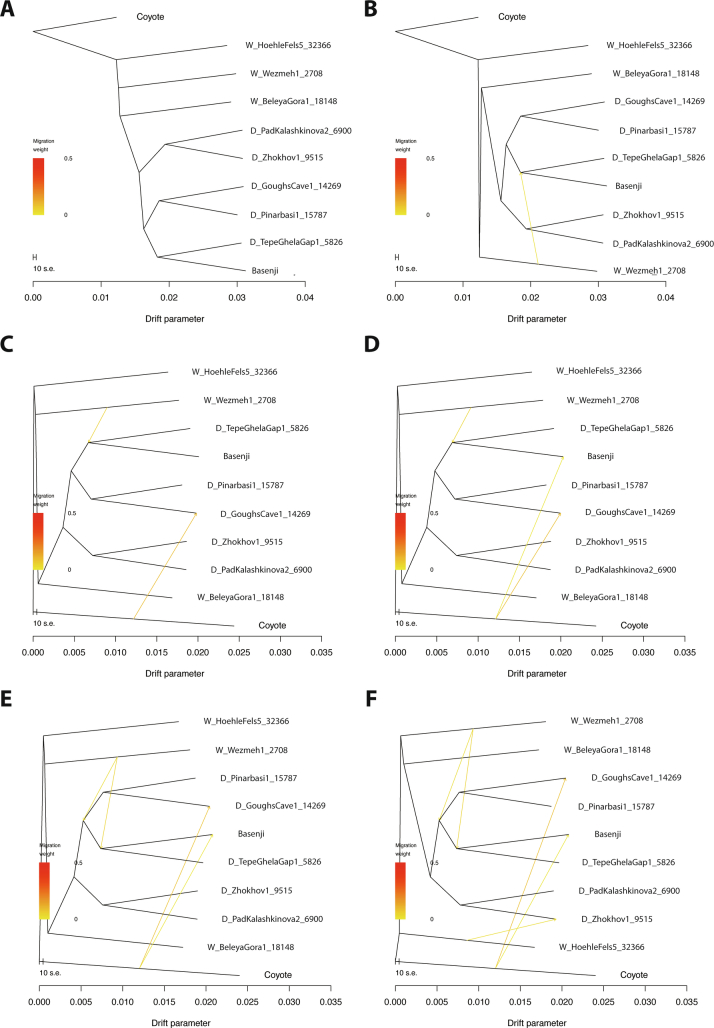
Admixture Graphs from Treemix. Maximum-likelihood phylogenies constructed in TreeMix v.1.13^60^ with (**A**) 0, (**B**) 1, (**C**) 2, (**D**) 3, (**E**) 4, and (**F**) 5 migration edges. See Supplementary Fig. 15 for residuals.

To visualize both the direction and number of these admixture events, we constructed admixture graphs using TreeMix^60^ (Extended Data Fig. 7 and Supplementary Figs. 14 and 15) and AdmixtureBayes^61^ (Fig. 3a and Supplementary Fig. 16). These results further support the conclusion that ancient Near Eastern (excluding Pınarbaşı) and African dogs possess mixed ancestry. Specifically, the two primary ancestry components are 96% western Eurasian dogs (represented by dogs from Pınarbaşı and Gough’s Cave) and 4% Near Eastern wolves (represented by the Wezmeh wolf).

Overall, our results indicate that Neolithic (and more recent) Near Eastern and African dogs possess more Near Eastern wolf ancestry than Palaeolithic and Mesolithic western Eurasian dogs. Although it remains possible that this wolf-related ancestry was the result of an independent domestication process^22,56^, it is more likely to result from geographically and temporally restricted gene flow between Neolithic and post-Neolithic western Eurasian dogs and Near Eastern wolves.

## Genomic legacy of Palaeolithic dogs

Our analyses indicate that both the Gough’s Cave and Pınarbaşı dogs derive most of their ancestry from the western Eurasian dog lineage, similar to Neolithic (and later Near Eastern) dogs. Previous work has shown that from the Late Neolithic onwards, European dogs possess mixed ancestry from both western and eastern Eurasian ancestry^22,56,58,62^. This shift in ancestry proportions has been attributed to an influx of dog ancestry from the East during migration of Steppe pastoralists associated with the Corded Ware and Yamnaya cultures of the Late Neolithic and Early Bronze Age^62^.

Nevertheless, ancient human genomes have indicated earlier migrations (before 9,000 years ago) of people with eastern hunter-gatherer genetic ancestry^14^ into eastern, central and southern Europe. Analyses of dogs from eastern hunter-gatherer contexts from north-eastern Europe (Veretye, Karelia) and the central Eurasian Steppe (Botai, Kazakhstan) have shown these individuals carried eastern Eurasian dog ancestry^63^. These results indicate that eastern Eurasian dog ancestry may have reached other parts of Europe earlier than previously thought, possibly during the Mesolithic.

To assess the degree of eastern Eurasian dog ancestry in western Eurasian dogs, we implemented a tri-source model of dog ancestry in qpAdm (Fig. 3b and Supplementary Fig. 17), using three ancestry sources based on the results of *D* statistics and admixture graphs: Palaeolithic western Eurasian (*D*_Pinarbasi1_15787) and eastern Eurasian (*D*_Zhokhov1_9515) dogs and Near Eastern wolves (*W*_Wezmeh1_2708). As expected, most ancient Near Eastern dogs possessed both western Eurasian dog and Near Eastern wolf ancestry. The eastern Eurasian dog ancestry component is absent until the Early Byzantine period (1,565 years cal bp at Marmara, Türkiye). By contrast, Balkan Mesolithic dogs from the Iron Gates in Serbia (Padina and Vlasac) and North-West Russia (Veretye) were best modelled as a combination of western Eurasian (mean 56.2%) and eastern Eurasian (mean 43.8%) dog ancestry.

Genomic analyses of Mesolithic hunter-gatherers from the same contexts as several of these dogs (Iron Gates and Veretye) have shown that they also possessed eastern hunter-gatherer genetic ancestry^14,64^. Coupled with the positive correlation between human–dog ancestries (Extended Data Fig. 3c), our results indicate that eastern Eurasian dog ancestry may have been introduced into Europe during the spread of eastern hunter-gatherers during the Mesolithic^14^, rather than during the later Steppe pastoralist migrations, as previously proposed^62^.

Our qpAdm analysis also indicates that the eastern Eurasian dog ancestry persisted in European dog populations from the Mesolithic to the present. Roughly 30% of this eastern Eurasian ancestry component is present in dogs during the Neolithic (for example, Pločnik, Serbia), 33% during the Bronze Age (for example, Belverde Di Cetona, Italy), 22% during the Iron Age (for example, Parknabinnia, Ireland), 18% during the Medieval period (for example, Vilnius Castle, Lithuania) and roughly 20% in modern breed dogs (Fig. 3b and Supplementary Fig. 17). Although this does not preclude further influxes of eastern Eurasian dog ancestry into Europe post-Mesolithic^58,62^, these findings show that the fundamental ancestry components (both eastern and western Eurasian ancestry) in European dogs were established by at least 10,900 years ago and persisted into modern breeds (Supplementary Fig. 17).

## Conclusions

Our results provide genomic evidence for the presence of genetically similar dogs in the UK, Germany, Italy, Switzerland and Türkiye during the Late Upper Palaeolithic (between 15,800–14,200 years cal bp; Figs. 1 and 2). The ancestry of this Palaeolithic population was retained in dogs throughout the Holocene (Fig. 3) and into modern breeds. We propose that this early dog population first spread across Late Upper Palaeolithic Europe alongside the expansion of Epigravettian-associated ancestry and material culture roughly 16,000 years ago. The presence of dogs in close association with humans carrying unadmixed Magdalenian-associated genetic ancestry (for example, at Gough’s Cave), however, indicates exchange of dogs between culturally distinct human populations during the Late Upper Palaeolithic in the absence of widespread gene flow or population turnover.

The Late Upper Palaeolithic dog population seems to have already been largely reproductively isolated from wolves. In fact, despite more than 15,000 years of the co-occurrence of dogs and wolves in western Eurasia, dogs have acquired little to no wolf ancestry aside from isolated instance(s) of admixture with wolves in the Near East that took place before 7,000 years ago. This contrasts with patterns of introgression seen in other domestic species, such as pigs and cattle, which interbred extensively with local populations of wild boar and aurochs following their introduction to Europe^65–67^. The near absence of wolf ancestry in ancient and modern dog genomes^68^ indicates that a substantial barrier to gene flow between wolves and dogs had been established in Europe and Anatolia by the Late Upper Palaeolithic. Combining many lines of evidence (for example, DNA, isotopes, material culture) that span the past 20,000 years across Eurasia will help to establish the precise phenotypic and cultural mechanisms responsible for the emergence of dogs, and their subsequent reproductive isolation from wolves.

## Methods

An expanded methods section that contains site and sample details, data generation and analysis is provided in the Supplementary Information. We sampled a total of 27 canid specimens (bone or tooth) from 6 archaeological sites across western Eurasia: Gough’s Cave (*n* = 7), Grotta Continenza (*n* = 1), Padina (*n* = 5), Pınarbaşı (*n* = 6), Vlasac (*n* = 4) and Wezmeh Cave (*n* = 4). Sample size was not statistically predetermined, and analysis was conducted without blinding or randomization.

### Radiocarbon dating

We generated eight new radiocarbon dates for canids from Gough’s Cave (*n* = 2), Pınarbaşı (*n* = 4) and Wezmeh Cave (*n* = 2) (Fig. 2b, Supplementary Fig. 3 and Supplementary Table 2). Collagen extraction and accelerator mass spectrometry dating was conducted at either the Oxford Radiocarbon Accelerator Unit (Oxford, UK) or the Higham laboratory at the Faculty of Life Sciences, University of Vienna (Vienna, Austria). Radiocarbon age estimates were calibrated using the International Calibration curve IntCal20 (ref. ^69^) in OxCal v.4.4 (ref. ^70^) and used to calculate mean age and 95% highest posterior density intervals.

### Stable isotope analysis

Bulk and compound-specific isotope analyses (δ^13^C and δ^15^N) were performed on both human (*n* = 4) and canid (*n* = 8) remains from Gough’s Cave and Pınarbaşı in the University of York’s BioArCh Facilities (Supplementary Table 6). Collagen extraction followed a modified^72^ protocol, with lyophilized collagen samples analysed in duplicate by elemental analysis-isotope ratio mass spectrometry (IRMS). Stable carbon (δ^13^C) and nitrogen (δ^15^N) isotope ratios were calibrated relative to the internationally defined standards (Vienna Peedee belemnite scale, AIR), with standard reference materials (Sigma fish gel, IsoAnalytical Alanine, IsoAnalytical Soy) used to monitor uncertainty. δ^15^N and δ^13^C values represent the mean of three standard-corrected measurements. For compound-specific isotope analysis, collagen for each sample was hydrolysed and filtered, before amino acids were derivatized to form *N*-acetyl-i-propyl esters following ref. ^73^ (alongside international reference standards and standards). Gas chromatography-carbon-IRMS measurements of amino acids were conducted using a Delta V Plus IRMS linked to a Trace Ultra 1310 gas chromatograph, and all data authenticated using observed versus expected bulk isotope values and observed hydroxyproline:proline δ^15^N and δ^13^C amino acid ratios (Supplementary Fig. 9).

### Ancient DNA data generation

Ancient DNA laboratory work was performed across dedicated clean-laboratory facilities in London, Munich (Ludwig-Maximilians-Universität; LMU) and Oxford.

### Ancient DNA laboratory, Natural History Museum, London

DNA was extracted from canid specimens from Gough’s Cave (*n* = 7) using a modified protocol version after ref. ^74^, with different preprocessing steps for teeth and bone. Double-stranded, dual-indexed DNA libraries were prepared from extracts following a modified^75^ protocol. Amplified indexed libraries were pooled in equimolar concentration for screening on an Illumina NovaSeq X Plus platform with paired-end (2× 150 base pairs (bp)) sequencing chemistry at Novogene. For extracts with higher endogenous DNA content and complexity, we generated further USER-treated libraries and deep sequenced these across several lanes of Illumina NovaSeq X Plus (2× 150 bp paired-end) at Novogene.

### Faculty of Veterinary Medicine, LMU, Munich

Samples from Pınarbaşı (*n* = 4) and Wezmeh Cave (*n* = 4) were subjected to a pre-lysis bleaching step^76,77^ before DNA extraction was performed semi-automatically using a Hamilton Microlab StarLet IV liquid handler (Hamilton) following a modified^78^ protocol. Double-stranded sequencing libraries were prepared using a modified^75^ protocol, with triplicate amplification using unique dual indices. Libraries were pooled at equimolar concentration for sequencing on NovaSeq X Plus lane(s) (25B flow-cell; 2× 150 bp paired-end) at Macrogen.

### Research Laboratory for Archaeology and the History of Art, Oxford

DNA was extracted from Padina (*n* = 5), Vlasac (*n* = 4) and Grotta Continenza (*n* = 1) material after ref. ^74^, and converted into double-stranded DNA libraries using the blunt-end single-tube protocol^79^. Purified libraries were dual-indexed^75^, and pooled at equimolar concentration for screening on an Illumina NextSeq 1000 (60 bp paired-end) at the Genzentrum (LMU). Deeper sequencing was then performed on an Illumina NovaSeq X Plus (150 bp paired-end) at Macrogen. We also performed mitochondrial capture on all samples from Padina, Pınarbaşı Vlasac and Grotta Continenza (*n* = 15) using a custom MyBaits Target Capture Kit (Arbor Biosciences) following a slightly modified High Sensitivity v.5 protocol, with captured pools sequenced on an Illumina HiSeqX Series (150 bp paired-end) at Macrogen.

### Ancient DNA data processing

First, to determine the taxonomic status of all ancient canids we used the canid identification (CanID) pipeline (https://github.com/lachiescarsbrook/CanID) with low-pass screening data as input^34^ (Extended Data Fig. 1). Raw reads from both newly deep sequenced and publicly available ancient genomes (Supplementary Table 1) were processed using nf-core/eager v.2.4.6 (ref. ^80^), using a modified version of CanFam3.1 including the Y chromosome as the reference. Ancient samples (*n* = 149) were then combined with an existing dataset containing more than 1,700 modern canid genomes, including 1,573 dogs and 122 wolves. To minimize the impact of DNA damage in ancient samples, we generated pseudo-haploid genotype calls by randomly sampling one high-quality read at each biallelic transversion site, which resulted in 7,454,020 single-nucleotide polymorphisms (SNPs).

### Mitochondrial DNA analysis

Majority consensus (75%) mitochondrial genomes were called for all ancient samples, and those with sufficient coverage (over 2×) were aligned with 220 publicly available ancient and modern canid mitogenomes (Supplementary Table 5) using MAFFT v.7.505 (ref. ^81^). From this alignment (15,457 sites excluding the control region) we constructed a maximum-likelihood tree in IQ-TREE v.2.1.4 (ref. ^82^) (HKY + F + I + G4; 1,000 bootstrap replicates) rooted against a coyote, and a time-calibrated Bayesian tree in BEAST2 v.2.6.7 (ref. ^83^). To ensure robustness of divergence time estimates in the Bayesian phylogeny, we excluded samples with no direct radiocarbon date (for example, Mesolithic Serbian dogs), and a depth of coverage of less than ten times (for example, Palaeolithic Gough’s Cave wolf; see Supplementary Table 4). The following priors were implemented in the Bayesian analysis: relaxed clock exponential (mean 1.0 × 10^−8^ substitutions per site per year, upper 1.0 × 10^−6^, lower 1.0 × 10^−9^), HKY + Γ4 substitution model, constant coalescent population tree model, lognormal kappa and exponential gamma shape, with default settings used for all other parameters. The clock model was selected after performing nested sampling for model selection comparing the relaxed clock exponential and a strict clock model, which favoured the relaxed clock exponential model. Posterior distributions of parameters were estimated through Markov chain Monte Carlo, with samples drawn every 10,000 steps over at least 1 billion steps (after discarding the first 15% as burn-in). Trace files were assessed using Tracer^84^ (to ensure the effective sample size exceeded 100) and samples from two independent runs were merged using LogCombiner. Finally, we estimated ages of samples without direct radiocarbon dates (Supplementary Table 4) using CanDate (https://github.com/mahaut-goor/CanDate-repo), a tool that performs semi-automated tip-dating in BEAST2 using a reference database (*n* = 192) of modern and directly dated ancient canid mitochondrial genomes.

### Population structure and admixture

We performed principal component analysis on the entire pseudo-haploid dataset (7,454,020 biallelic variants across 426 dogs and wolves) using smartpca (EIGENSOFT v.8.0.0, ref. ^85^), with ancient individuals projected into principal component space through a least-squares approach (lsqproject YES; Supplementary Fig. 4). We also performed principal component analysis using EMU v.0.9 (ref. ^86^) on a subset of the pseudo-haploid dataset that contained exclusively ancient dogs and wolves (7,454,020 biallelic variants across 146 individuals, ‘ancient-only’; Fig. 1c).

Shared drift was computed between all possible combinations of ancient and modern dogs and wolves using outgroup-*f*_3_ comparisons of the form *f*_3_(X, Y; Coyote) as implemented in the Calc-f3 function in struct-f4 (ref. ^87^) (Extended Data Figs. 3–5 and Supplementary Fig. 18). Spatial patterns were then identified through linear regression against geographic distance computed as great-circles (Haversine) distances between archaeological sites under comparison. We applied multiple regression on distance matrices^88^ to assess the influence of spatial and temporal distance on an outgroup-*f*_3_ similarity matrix assemblage from dogs over 0.1× coverage (*n* = 63). We applied a *z* transformation to spatial and temporal distance matrices, standardizing each to have a mean of zero and a standard deviation of one (Supplementary Table 3). We determined statistical significance by means of permutation testing, highlighting dog pairs with residuals exceeding four standard deviations from the mean, which may indicate potential outlier observations.

We performed model-based estimation of population structure using the algorithm implemented in ADMIXTURE v.1.3.0 (ref. ^89^). We used the ancient-only dataset, and ran ADMIXTURE using *k* = 2, with 50 bootstrap replicates (Supplementary Fig. 5).

### Relatedness

We measured genetic kinship coefficients (*θ*) between ancient European (*n* = 31) and Near Eastern (*n* = 12) dogs using READv2 (ref. ^90^), with default normalization (median) and block jackknife window-size (5,000,000) parameters^91^ (Supplementary Figs. 7 and 8). We also performed pairwise kinship analysis on ancient humans from Europe (*n* = 21) and the Near East (*n *= 11), which were either recovered from the same archaeological contexts as the dogs, or were the closest geographic or temporal proxy. Pseudo-haploid genotype data for ancient humans were collated from the Allen Ancient DNA Resource v.64 (ref. ^92^).

### Dog–human ancestry co-analyses

We extended analyses of shared evolutionary histories between humans and dogs to the entirety of Eurasia and North America. We collated pseudo-haploid genotype data for ancient humans from the Allen Ancient DNA Resource^92^ at Eurasian and North American sites (exceeding 500 years old) where both human and dogs were present (*n* = 35; Supplementary Table 7), before running all possible pairwise *f*_3_-outgroup comparisons. We standardized the data to have zero mean and variance one, and evaluated the correlation between the two corresponding matrices using a partial Mantel test while controlling for space and time. Partial Mantel test allows assessing the association between two distance matrices while statistically controlling for the underlying spatial and temporal structure. Thus, any significant positive correlation would be consistent with shared evolutionary histories between humans and dogs, beyond that expected by isolation by distance^93^ and time alone. Furthermore, we evaluated the distribution between dog and human *f*_3_-outgroup comparisons, and identified pairs that deviated significantly (|*Z* | > 3) from the median absolute deviation of all paired distances. Following suggestions in ref. ^94^, concerning inflated type I error rates when both similarity matrices show strong spatial autocorrelation, we consider a conservative significance threshold 0.0045. Using this corrected *α* level, our observed association (*P* = 0.0001) remains significant, indicating that the relationship cannot be explained by shared spatial or temporal structure alone.

### *D* statistics

We calculated *D* statistics using the qpDstat function implemented in AdmixTools v.7.0.2 (ref. ^95^) using inbreed, YES to account for pseudohaploid genotypes (Supplementary Figs. 11–13, 19 and 20). Standard errors were estimated by performing weighted block jackknife over 5 Mb blocks, and used to calculate *Z* scores. To quantify the proportion of Near Eastern wolf ancestry in both ancient Near Eastern dogs and the Basenji (*X*), we performed *F*_4_-ratio estimation using AdmixTools (qpF4ratio) with the following setup:

$$\alpha =\frac{{F}_{4}(D\_{\rm{Zhokhov}}1\_9515,\,{\rm{Coyote}};X,\,W\_{\rm{Wezmeh}}1\_2708)}{\begin{array}{c}{F}_{4}(D\_{\rm{Zhokhov}}1\_9515,\,{\rm{Coyote}};D\_{\rm{Pinarbasi}}1\_15787,\\ \,W\_{\rm{Wezmeh}}1\_2708)\end{array}}$$

Jackknife block size was set to 100 SNPs (blgsize, 0.01). Mean admixture proportions (*α*) and *Z* scores were calculated for each Near Eastern dog, with only significant (*Z* > 3) comparisons considered (Extended Data Fig. 6b).

### Ancestry modelling

We constructed admixture models using TreeMix^60^ (Extended Data Fig. 7 and Supplementary Fig. 15) and AdmixtureBayes^61^ (Fig. 3a and Supplementary Fig. 16) with representatives from East (*D*_Zhokhov1_9515, *D*_PadKalashkinova2_6900) and West (*D*_Pinarbasi1_15787, *D*_GoughsCave1_14269, *D*_TepeGhelaGap1_5826, Basenji) Eurasian dog lineages, an ancient Iranian wolf (*W*_Wezmeh1_2708) representative of Near Eastern wolf ancestry and Pleistocene East (*W*_BeleyaGora1_18148) and West (*W*_HoehleFels5_32366) Eurasian wolves. We first estimated a maximum-likelihood tree without admixture based on allele frequencies, and then added 1–4 migration edges (*-m*), with all trees rooted against the coyote (Extended Data Fig. 7). Block size for covariance estimation was set to 100 SNPs (*-k*), with a total of 1,000 bootstrap replicates (*-*bootstrap) performed. We then ran AdmixtureBayes^61^ using the same input allele frequencies, and modelled 1–3 admixture events, with each run in triplicate for one million generations using eight Markov chain Monte Carlo chains.

We also modelled the ancestry of ancient western Eurasian dogs using supervised ADMIXTURE analysis (--supervised) with two ancestral components: eastern Eurasian dogs (*D*_Khatystyr1_9682, *D*_PortauChoix1_4157, *D*_ShamankaII1_7400, *D*_Zhokhov1_9515, *D*_PadKalashkinova2_6900) and Near Eastern wolves (grey, Wolf07, Wolf20, Wolf55, Wolf56, Wolf70, Wolf71, Wolf72, *W*_Wezmeh1_2708) (Supplementary Fig. 14).

qpWave and qpAdm analysis was performed using AdmixTools v.7.0.2 to test ancestry models for dog populations across Eurasia. First, qpWave was used to test the cladidity of several combinations of dogs (see Supplementary Information for full details). We used a set of pre-LGM Pleistocene wolves (*n* = 7) as reference populations given (1) likely asymmetrical relationships with dogs, and (2) no chance of admixture between these wolves and dogs, before extending analysis post hoc to test extra reference population combinations. qpAdm was used to model the ancestry of both modern and ancient western Eurasian dogs using three potential source populations: *D*_Pinarbasi1_15787 (representing western Eurasian dog ancestry); *W*_Wezmeh1_2708 (representing introgressed ancestry from Near Eastern wolves) and *D*_Zhokhov1_9515 (representing eastern Eurasian dog ancestry). To ensure these source populations were the best fit for non-western Eurasian dog ancestry, we calculated *D* statistics of the form *D*(Coyote, *W*_Wezmeh1_2708, *X*, *D*_Pinarbasi1_15787) and *D*(Coyote, *D*_Zhokhov1_9515, *X*, *D*_Pinarbasi1_15787) (Supplementary Fig. 13).

Finally, the set of reference populations was modified to include a dog from the Americas dating to 4,500 years ago and a dingo, ensuring differential relationships of each reference population to both source and target individuals. We ranked models based on *P* values for single, dual and triplet sources, and rejected all models with *P* < 0.01 or whose ancestry proportions contained negatives (that is, impossible). We used allsnps: YES for all qpAdm and qpWave analyses to maximize the number of SNPs used to calculate *F*_4_ statistics thus improving power to reject models. Mean ancestry proportions for Mesolithic, Neolithic, Bronze Age, Medieval and Modern period dogs were calculated using dogs for which the *P* value for a dual-source western Eurasian–eastern Eurasian model was more than 0.01.

### Reporting summary

Further information on research design is available in the Nature Portfolio Reporting Summary linked to this article.

## Online content

Any methods, additional references, Nature Portfolio reporting summaries, source data, extended data, supplementary information, acknowledgements, peer review information; details of author contributions and competing interests; and statements of data and code availability are available at 10.1038/s41586-026-10170-x.

## Supplementary information

Supplementary Information This file contains detailed descriptions on the background, methodology and/or results for: (1) site descriptions, (2) radiocarbon dating, (3) stable isotope analysis and (4) ancient DNA, as well as Supplementary Figs. 1–22.

Reporting Summary

Supplementary Tables Supplementary Tables 1–7.

Peer Review File

## Data Availability

We used the CanID pipeline to determine the taxonomic status of all ancient canids, which is available through GitHub at https://github.com/lachiescarsbrook/CanID; and we estimated ages of samples without direct radiocarbon dates using CanDate, which is available through GitHub at https://github.com/mahaut-goor/CanDate-repo. All ancient DNA sequencing data generated for this study are deposited in the European Nucleotide Archive (ENA) (PRJEB104454) under the accession codes SAMEA120632851–SAMEA120632875. Raw radiocarbon age estimates are reported in Supplementary Table 2. Raw isotopic measurements are reported in Supplementary Table 6. All newly generated ancient sequences (SAMEA120632851–SAMEA120632875) are available in the ENA under BioProject no. PRJEB104454. All other data are included in the paper and/or the Supplementary Information.
