# Supplementary material for: Dogs were widely distributed across western Eurasia during the Palaeolithic
Source: Nature. 2026 Mar 25;651(8107):995–1003. doi: 10.1038/s41586-026-10170-x (PMC13017512; doi:10.1038/s41586-026-10170-x)
Supplement: Supplementary file 1 — This file contains detailed descriptions on the background, methodology and/or results for: (1) site descriptions, (2) radiocarbon dating, (3) stable isotope analysis and (4) ancient DNA, as well as Supplementary Figs. 1–22. [file 41586_2026_10170_MOESM1_ESM.pdf]

nature portfolio

https://doi.org/10.1038/s41586-026-10170-x

Supplementary information

Dogs were widely distributed across western Eurasia during the Palaeolithic

In the format provided by the authors and unedited

1. Site Descriptions and Sample Information 3
Gough's Cave, United Kingdom 3
Pınarbaşı, Türkiye 5
Grotta Continenza, Italy 9
Wezmeh Cave, Iran 10
Vlasac, Serbia 11
Padina, Serbia 12
2. Radiocarbon Dating 14
University of Vienna, Austria 14
Oxford Radiocarbon Accelerator Unit, UK 14
3. Stable Isotope Analysis 15
Compound-specific isotope analysis 15
δ^{15}N measurements of AAs 16
δ^{13}C measurements of AAs 17
Quality Control indicators 17
4. Ancient DNA 19
Sampling 19
DNA Extraction, Library Preparation and Sequencing 19
Natural History Museum (Gough's Cave) 19
Ludwig Maximilian University, Munich (Pınarbaşı, Wezmeh Cave) 19
Oxford University (Padina, Vlasac, Grotta Continenza) 20
Raw Data Processing 21
CanID Taxonomic Identification 21
Genotype Calling and Merging 22
Mitochondrial Phylogenetics and Molecular Dating 22
Population Structure and Admixture 23
Genetic similarity and relatedness 25
Dog-Human Ancestry Comparison 26
AMY2B Gene Copy Number Estimation 27
D-statistics 27
TreeMix 27
AdmixtureBayes 28
F4-ratio 28

qpWave and *qpAdm* 28
Basal ancestry in Gough's Cave dog 31
Outgroup Selection 32
5. Supplementary Figures 33

1. Site Descriptions and Sample Information

Gough's Cave, United Kingdom

Gough's Cave (51.282100, -2.765700) is an archaeological site situated in Cheddar Gorge, a deep limestone gorge in the Mendip Hills of west Somerset. Of the numerous caves in the gorge, Gough's Cave is the largest with a maximum depth of 155 m and a length of over two miles. It can be found at the base of the southern cliff face at the lower western end of the gorge around 85 m a.s.l. A section of the Cheddar Yeo, the largest underground river system in the UK, runs through the cave. The first written records of the cave date to 1892, with its rediscovery attributed to Captain Richard Gough, who subsequently excavated the cave and opened it to the public at the turn of the century. Numerous formal archaeological excavations have been performed over the last century that uncovered multiple stratigraphic horizons containing rich lithic industries and both human and faunal remains attributed to the late Upper Palaeolithic [1–4].

The largest assemblage excavated from the site is attributed to the late Upper Palaeolithic culture of the Magdalenian [1,3,5–7]. Human and faunal skeletal elements have been directly radiocarbon dated, with Bayesian recalibration of material from the Upper Palaeolithic horizon (IntCal20 curve; [8]) determining site usage to have begun between 15,070 and 14,850 calBP and cease between 14,960 and 14,610 calibrated years Before Present (calBP) (95% CI) [9]. This dates cave usage to a period directly preceding the start of the Bølling–Allerød interstadial, a period of warming at the end of the Last Glacial Maximum (LGM). Human material found in this Magdalenian horizon show evidence of anthropic modifications, with cut marks, tooth marks, and percussion damage identified on over 60% of all cranial and post cranial remains [10,11], indicative of cannibalism. Three human cranial vaults were carefully modified to form human skull cups. The cannibalism at the site has been interpreted as funerary cannibalism, and has been identified at several other Magdalenian sites [12]. More generally, the LUP Magdalenian groups deposited at Gough's Cave are considered the most Northwesterly examples of this LUP culture, that is seen to spread out of glacial refugia in southern France and Northern Spain as early as 20,000 years ago, into regions of Central and Eastern Europe. This culture is seen across these regions from around ~18,500 years ago, with Gough's Cave representing one of the latest examples of the culture, before its disappearance in the archaeological record. Such disappearance has been attributed to wide-scale population turnover across West Eurasian, where Epigravettian groups are seen to replace the Magdalenian both culturally and genetically. Whilst the assemblage at Gough's Cave is considered a single deposition, a potential secondary, later occupation of the cave by Palaeolithic humans associated with the *Fessermessergroupen* has been suggested from a single later radiocarbon date [13].

23 bone elements (MNE=19) attributed to *Canis lupus* have been excavated from the Magdalenian horizons at Gough's Cave. Three of these *Canis lupus* samples (**NHMUK PV M13794(a, b)**, **NHMUK PV M50013** and **NHMUK PV M49877** were incorporated into

all elements of this study (Supplementary Table 1; Supplementary Figure 2). These elements were selected given (i) their association with the Magdalenian horizon, (ii) their reduced size that can often indicate a domestic origin, (iii) observed anthropic modification (cut marks and perforation), and (iv) regions present on the material of suitable size and quality for AMS, DNA and CSIA sampling.

NHMUK PV M13794 (a,b): This left hemi-mandible is almost complete, with the exception of the anterior portion of the horizontal ramus, which has an oblique break between the alveoli of the P_3. Missing parts of the coronoid process and the angular process are associated with carnivore chewing marks. The corresponding, more complete right hemi-mandible (1.2./16.) is preserved in the archives of Longleat House (Wiltshire, UK). The masseteric fossa of both hemi-mandibles are perforated by sub-circular openings. These perforations are ancient in origin and were created by carefully placed percussion blows. This specimen has previously been classified as a domestic dog from morphometric analysis given its reduced size compared with Palaeolithic and modern wolves (Supplementary Figure 2). The Longleat specimen also preserves butchery cut marks, with patterns of tooth wear identified on this specimen showing strong similarities with that identified on a putative dog from Bonn-Oberkassel and dated to a similar period [14]. The Bonn-Oberkassel specimen has previously been associated with the Magdalenian, although recent reassessment of the genetic ancestry of individuals at the site (Villabruna associated) and the funerary behaviour (burial) suggests an Epigravettian/Federmessergroupen context [12].

NHMUK PV M49877: This right hemimandible is near complete, with the exception of the coronoid process and the angular process. The specimen has previously been published as a domestic dog [13]. No teeth are preserved in-situ. The specimen has been radiocarbon dated previously (OxA-13585; 12,440 ± 55BP; 14900 to 14100 calBP) and was found in the same context as NHMUK PV M13794 and NHMUK PV M50012-15. This corresponds with other dates from the Gough's Cave Magdalenian assemblage.

NHMUK PV M50013 [M50012B]: This specimen consists of a partial right maxilla (NHMUK PV M50013) portion found during the same phase of excavation and within the same level (spit 16) as three other canid elements (M50012, M50014, M50015). These three elements refit (M50012 a,c,d), with M50013 (M50012 b) believed to have been joined through the interlocking maxilla/premaxilla suture and the upper second incisor (NHM UK PV M50012d) with its respective socket prior to sampling in 2003. None of these elements exhibit ancient anthropic modifications.

We also generated low coverage sequence data from a further four canid specimens from Gough's Cave **[NHMUK PV M13795, NHMUK PV M13796, NHMUK PV M50014 and NHMUK PV M50015]**, although data was too low quality (0.001%> endogenous) for genetic analysis.

Pınarbaşı, Türkiye

Pınarbaşı (37.4939, 33.0192) is located in south central Anatolia at the southeast edge of the western Konya basin, an inland drainage basin to the north of the Taurus mountains at c. 1000 m above sea level. Much of the basin was an extensive lake during the Pleistocene, and at some point following the Last Glacial Maximum (LGM) the lake dried and smaller lake bodies, that fluctuated in size, were left around the edge of the basin. Pınarbaşı is at the end of a range of low limestone hills that mark a transition in the landscape from plain to hill. The site consists of a number of components. Against the face of the limestone cliffs are a series of rock shelters and caves; in the most northerly of which trench B was excavated, in which an Epipalaeolithic sequence was documented in 2004 that contained human material alongside fish, mammal, and bird remains. Original excavations were conducted by Trevor Watkins in 1994 and 1995 and by Douglas Baird in 2003 and 2004. Renewed excavations are now being undertaken by Baird and Mustafaoğlu, but these are not reported here in any detail.

The Epipalaeolithic of SW Asia in broad terms dates c. 20000-11700 calBP contemporary with the Late Upper Palaeolithic in Europe. The earliest dates for the Epipalaeolithic occupation at Pınarbaşı, relating to the deposition of canid remains, are early in the sequence and fall somewhere in the range c. 16100-15700 calBP. Other C14 dates coming from early in the stratigraphy include two *in situ* burials in Graves 13 (15600-15200 calBP) and Graves 14 (15200-14200 calBP; [15]), although the greater part of the Epipalaeolithic occupation sequence was deposited after 15200–14200 calBP (Grave 14). Three caprine bones from the later, upper 0.75m of the Epipalaeolithic sequence cover the range 13400-12900 calBP [15]. When dates are compared with recent dating of the onset of the Glacial Interstadial 1 (GI-1) and beginning of the Early Natufian in the Levant, we can see that the earliest dates (canid remains c. 16.1k-15.7k and 15800-15600 calBP) and the dates for the burial in Grave 13 (15600–15200 calBP) predate the GI-1 and the beginning of the Early Natufian, with later deposition (Grave 14) likely to coincide with the beginning of, or earliest, Natufian and possibly the GI-1.

There are several human burials at the site of which 2 were excavated in the 2004 season. The burial in Grave 13 was identified as a c. 25–29-year-old male (15600-15200 calBP). Most notably the cranium of this individual had been removed, which, given the presence of the maxilla within the grave, occurred after a period of initial burial and decomposition. No later disturbance of the burial that could account for the accidental removal of the cranium was identified, and as such, this is the earliest instance of clear-cut, post-burial cranium retrieval in SW Asia: a clear connection between those likely reoccupying the site after some years and the dead was emphasised by this act [15], whilst also suggesting that such connections with ancestors, so typically associated with Neolithic practices, may have little to do with sedentarisation behaviours. A later adult male burial (Grave 14; [16]) was particularly distinctive because it was accompanied by a substantial collection of grave goods.

aDNA analyses relating to one individual from Pınarbaşı, burial context code ZBC from Grave 13 demonstrate that the Pınarbaşı population shared ancestry with both European

Upper Palaeolithic and Levantine gene pools, reflecting gene flow between southeast Europe and southwest Asia that predated 15500 calBP [17]. This would encompass some gene flow between Anatolian communities and those of the Epipgravettian of southeast Europe and those of the Kebaran Levant.

The presence of marine shells in high frequency at Pınarbaşı suggests exchange with communities exploiting the south coast of Anatolia probably via intermediaries and raises the possibility of interactions with contemporary groups using the edge of Cilicia to the south or the Antalya area to the southwest. The closest definitely dated Epipalaeolithic site is Eşek Deresi *ca* 70km to the southeast as the crow flies, but on the southern edge of the Taurus so a significant distance to walk through the Taurus passes. This site dates to the GI-1 and the GS-1 Younger Dryas [18], overlapping with and post-dating Pınarbaşı but there may be a longer occupation revealed at the site when more excavation is carried out and more dates are available. Even if no earlier occupation is found at this site the community using it probably operated in the same area in the preceding more mesic GI-1. Lithics suggest closer relationships between the Cilician communities and Pınarbaşı than with Antalya sites, given the importance of lunates at Eşek Deresi and small scrapers [18], thus very like the Pınarbaşı chipped stone assemblage, although the main raw material was chert. Obsidian is also present, which shows clear links with the plateau, of probably greater intensity than the Antalya case where obsidian is rare.

It is also notable that a number of ritual, symbolic, technological and thus social, practices at Pınarbaşı strongly recall Natufian practices, suggesting interactions were likely to have occurred between plateau groups and those operating to the south and east. Shared technological practices (lunate predominance and the micro-burin technique), symbolism and ritual with the Late Epipalaeolithic communities of the Levant and shared hunting practices such as the likely frequent use of spear throwers, suggests exchange with the southeast, probably up and down the Göksu, with marine shells moving northwest and possibly obsidian moving southeast, where it may have made its way to Levantine sites, as exemplified at Late Natufian contemporary Abu Hureyra [19] and Final Natufian at Ain Mallaha.

Connections to the west are much less clear. Obsidian from central Anatolia, which may have passed through the Konya plain, does reach the southwest coast of Anatolia, but in low frequencies in the contemporary Antalya caves. Cappadocian obsidian, from both Nenezi and Göllüdağ East, has been found in, probably unmixed, late Epipalaeolithic deposits at Öküzini in GH Ia2 [20], probably dated to c. 15,000–13,000 calBP. Chipped stone assemblages are rather different, with backed bladelets, non-geometric microliths as well as geometrics, but much less common lunates. In southeastern Europe Epigravettian assemblages are diverse and cover a long time period, LGM to end of Younger Dryas at least, as relevant to discussion here. None of the Epigravettian geographical and chronological variants, encompassing backed blades and bladelets, shouldered points and microliths of non-geometric and geometric types show any similarity to the Pınarbaşı assemblage. A few Late Upper Palaeolithic/Epigravettian

burials occur in rock shelters, caves and settlements, or as part of small cemeteries, often with shallow graves in southern and eastern Europe. In the Late Upper Palaeolithic of Italy, for example, many of these burials are supine and extended, accompanied by tools and/or prolific ornamentation and other grave goods, often dentalium or other shell beads [21]. Ochre and engraved stones are associated with some burials (e.g. a male in Riparo Tagliente, Veneto) [21]. Secondary manipulation, especially skulls, is seen at Arene Candide in Italy [22]. Thus, there are some broad similarities in place-making burials and some secondary manipulation of skulls and rich burials with ochre and marine shells, these do not match to the same degree some of the specific similarities between Pınarbaşı and Natufian burials, which are presumably all part of an extensive Late Upper Palaeolithic tradition shared widely across the Mediterranean and eastern Europe. In short there are major cultural contrasts with the Epigravettian assemblages of chipped stone and indeed exchange networks.

Analysis of plant and animal remains from the site allows some reconstruction of the palaeoenvironment around the site and can be set within a broader palaeoenvironmental reconstruction from geomorphological work. The site was located at an ecotone, a transitional area between wetlands, woodland/forest and steppe. The presence of extensive local wetland is indicated by a high frequency of wetland plant and animal species with 98% of the birds being wetland species [23], with marsh frogs, water snakes and water vole predominating in the microfauna [24]. However, several birds, mammal and tree species suggest rocky and hill, steppe and woodland habitats which match areas immediately east of the site [15,16].

Thin ashy and organic rich occupation deposits separated by layers of limestone cliff face rock shatter, occasional hearths, shallow small pits and sporadic burials at different points in the sequence are characteristic of human activity at the site. No clear evidence for habitation structures has been found at the site, although dense occurrences of reed and sedge phytoliths point to the possibility of light structures, such as screens, constructed from such vegetation. The earlier phases of the occupation have more, thicker midden like, organic deposits, richer in charcoal, as if the occupation became more episodic or occupation episodes were shorter/less intense through time. Chipped stone is low in density at the site [16], although it occurs regularly through the whole sequence, with some lenses that show somewhat greater concentration, evidencing short periods of slightly more concentrated activity.

Carbon and nitrogen isotope evidence, notably the substantial trophic level difference between human and animal nitrogen isotopes, especially compared to the early Holocene evidence from Boncuklu and Pınarbaşı, strongly suggest significant meat/fish/aquatic components of the diet [16,25]. It seems likely fish and water birds may have been particularly important within the diet given their and human elevated nitrogen isotope levels [16] although the contribution of these animals to the diet is more difficult to quantify as against larger mammals. Wetland birds were an important food source within the avifauna [23], with small, probably netted fish remains regularly retrieved from the 1 and 2 mm heavy residues, attesting to their ubiquity. Frog, water vole, snake and tortoise were

also present in the deposits and probably also consumed [24]. When based at Pınarbaşı, people hunted significant quantities of sheep and goat mainly in the wooded hills behind the site (caprines 35% of NISP of bones of identified species), where they probably also hunted deer and the occasional aurochs in the wetlands, and equids across the steppe [16].

Plants including wild lentils, hackberries, terebinth and probably almond also played a dietary role, though likely less important than meat. The absence of the wild ancestors of cereal cultivars is notable. This modest plant contribution is suggested by both carbon and nitrogen isotopes and their low ubiquity and frequency in carbonised remains and seed/nut remains, whether as carbonised remains or phytoliths. Humans at Pınarbaşı certainly exploited a wide range of resources underpinning their ability to reside at the rock shelter on a multi-seasonal episodic basis, in a form of 'broad spectrum' economy well matched to local resource availability, but apparently under-exploiting plant availability. In addition to the economic and food acquisition aspects of animal and plant exploitation, we should also consider the attractiveness of food and cuisine diversity to the community. This should include considerations such as the provisioning of larger scale food consumption and/or ritual events providing a background to occasional killing of larger animals such as the aurochs and equids, or acquiring some animals for ritual practice, possibly some birds [23].

Canids are very common in the Epipalaeolithic at Pınarbaşı, with contextual information indicating that these individuals were early examples of domestic dogs. Canids form 53.5% of NISP (of bones identifiable to species N=245) from the overall sequence [16]. Since many of the juvenile canid bones were articulated or partially articulated groups, this NISP is very misleading and must represent an overestimate of canid presence. Canid MNI is 4 but there were at least 5 discrete canid pup burials, which suggests that MNI is conversely a major underestimate of canid presence at the site. Thus, canids were clearly unusually common within the assemblage. A high proportion of the canid bones are from perinatal and very young individuals but there are also 11 additional bones from older, sub-adult and adult individuals.

Four canid specimens were incorporated into the project (Supplementary Figure 1):

- **PB04 BIF**
- **PB04 BIF 123 #4081**
- **PB04 BIB 115 #4057**
- **PB04 BIE AC09**

The specimens selected for aDNA analysis came from a series of contexts described in detail in this paragraph. At least 3 canid burials are attested in context BIF which was a discrete oval concentration of dark organic sediment with very small charcoal fragments and flecks of red ochre. The feature seemed to be a shallow scoop or depression in surrounding deposits, in which debris from an episode of human occupation was preserved and was a focus for the burial of the pups. This scoop/depression with the pup burials overlay the lower legs of the male buried in Grave 13. Whilst this feature, with fill

BIF, clearly postdates the burial the proximate juxtaposition of the pup burials and the adult male human is particularly notable. Clusters of perinatal- young canid bone in context BIE were probably also dog pup burials. BIE was a context in the middle of the excavated sequence postdating Grave 14. It was a mix of rock shatter debris and lenses of darker more organic material and charcoal deriving directly from human activity. Context BHL was in the upper part of the sequence dating c. 13000 calBP. BHL consisted of a layer of rock face shatter with a mixture of brown sediment, with darker areas of more organic material. Chipped stone and animal bone were scattered through this deposit, which suggested natural accumulation with episodic human activity. All of these canid burials in BIF, BIE and BHL seem to have been of pups, both neonates and very young individuals only a few weeks old.

Unlike other instances of young canid burials, for example in the Levantine Natufian [26], these pup burials do not seem to be directly buried with the human inhumations at the site. In fact, they seem to both predate and postdate those burials, sometimes by very significant amounts of time (for example, context BHL). On the other hand, at least in the case of BIF there is a close spatial and stratigraphic association between a group of pup burials and an earlier human burial. That the community retained memory of the location of the human burials is attested by the act of cranial retrieval in the very case of Grave 13. In this case it is certainly plausible that the pup burials referenced the earlier grave. More generally it seems that this restricted area of the rock shelter was a focus for human and canid burials for very long periods, further evidenced by the discovery of further human burials in this very restricted area of the rock shelter in recent excavations, albeit human burials seem concentrated in the earlier part of the sequence. Such persistent practices in spatially discrete areas within the settlement zone would seem to interrelate the treatment of the humans and dogs and suggest that the dog burials are anthropogenic and both purposive and ritualised.

Given the relatively frequent presence of buried canid remains, the lack of evidence for carnivore gnawing on the other animal remains is notable [16] with access to foods for these putative dogs seemingly carefully controlled by humans or perhaps dogs were located outside the immediate settlement and burial area. Further, there is a high proportion of canid feet and mouth elements amongst the 11 sub-adult to adult bones which suggests that complete bodies did not contribute significantly to this canid sub-adult/adult assemblage at the site, at least in this studied assemblage. It does raise the possibility that canid/dog skins may have been utilised, alongside other uses.

Grotta Continenza, Italy

La Grotta Continenza is a cave site on the southern side of the Fucino basin of central Italy (41.958929, 13.542322) that has delivered archaeological material from Pleistocene and Holocene horizons [27]. The basin is a now dry palaeolake, with the cave situated on the northern side of Mt. Labrone overlooking the basin. Throughout the Late Pleistocene period, the water level would have oscillated considerably, before drying out between the Roman period and late 19th century [28]. The cave is found at the base of a limestone cliff,

and is 20m wide by 8m deep. Recovered material from the sites signifies cave usage over the last 20,000 years, with evidence of use by Epigravettian, Mesolithic and Neolithic populations [29].

Epigravettian occupation is attested to with several heaths and abundant lithics during the early Epigravettian periods (EP1 and EP2; max. 15600 calBP [LTL6187a] to min. 13300 calBP [R-1198]), with burials beginning to appear in the late Epigravettian horizon (EP3; max. 12600 calBP to 10800 calBP) and at least 7 individuals represented [30].

One canid specimen from a Palaeolithic horizon was incorporated into the project:

Taglio38: a single *Canis lupus* from an Epipalaeolithic horizon (layer 38). Although no material from this layer have been directly dated, layers above and below have direct radiocarbon dates: [Layer 37 - LY10755 = 11,830±110] and [Layer 40 - LY10754 = 11560±100] indicative of a Late Epigravettian occupation [29]. Layers 31 to 44 are associated with the Late Epigravettian, and include multiple human burials, hearths and abundant lithics.

Wezmeh Cave, Iran

Wezmeh Cave is an archaeological/paleontological site in Western Iran (34.058575, 46.64795), that has delivered a series of archaeological horizons since its initial discovery in 1999. The cave is approx. 45 m2 with a depth of 27 m, and has an elevation of 1.430 m above sea level [31]. It is located in a steep valley slope of the Kermanshah Province, in an area dominated by a contrasted topography, rocky outcrops and low vegetation [32].

Unfortunately, prior to its discovery during an archaeological survey of the region by a team under general direction of Kamyar Abdi, the cave was subject to looting by illegal diggers, who disturbed the faunal material's position in the stratigraphy. Since then, several excavations have been performed at the site over the last 25 years [33,34], revealing a large deposit of faunal remains across several distinct archaeological horizons, with carnivores in particular in great abundance. This indicates its persistent use as a den by wolves, hyenas, foxes, bears, and visits by at least ten species of carnivores in total [31,32,35,36]. This makes Wezmeh one of the only known important fossil carnivore accumulation for the Pleistocene record in the Zagros. Fragmented human remains have also been excavated from both Holocene and Pleistocene horizons, with a single human premolar attributed to *Homo neanderthalensis*, discovered of a size that places it at the upper limits of Late Pleistocene human variation [37,38].

Uranium-series and radiocarbon dating techniques have been performed on both faunal and human material, with animal material dating to between 70,000 and 11,000 years ago, and a single human metatarsal bone dating to 9460-9010 calBP. Genetic data from this human metatarsal shows the individual (WC1) carries similar patterns of shared drift with individuals from Neolithic burials at Tepe Abdul Hosein (in Iran) from a similar time

period (approx 10500-9500 calBP), indicating this individual was a member of a largely homogeneous human population in the region [39].

Canis lupus remains (NISP: 176; MNI: 6) have been excavated from the late Pleistocene layers [31]. Teeth are well represented, and all fall within the size variation of wolves. No Canid material had been dated prior to this study, with the aforementioned U-series dating restricted to six specimens (three *Crocuta crocuta*, two *Ursus arctos*, and one *Sus scrofa*).

Two canid specimens were incorporated into the project, both of which came from the initial gathering of the faunal material in 1999 and 2001:

- **WZ-77:** a lower second left premolar, showing moderate use-wear.
- **WZ-190:** a lower right canine, also showing moderate use-wear.
- **WZ-189:** a lower right canine, also showing moderate use-wear.
- **WZ-194:** a lower left canine, also showing moderate use-wear.

Vlasac, Serbia

Vlasac (44.533333, 22.033333) is an archaeological site in Serbia, on the western banks of the Danube river, in the middle part of the Danube Gorges, which is called Gospodjin Vir. The region is rich in open-air Early Holocene settlements, best known for extraordinary artworks in the form of sculpted boulders, trapezoidal dwellings with reddish limestone floors and numerous burials within settlements, often placed beneath dwelling floors [40–45]. Chronostratigraphy is based on intensive radiometric dating and generally recognizes the following phases in the occupation: Early Mesolithic (11500-9500 calBP), Late Mesolithic (9500-8300 calBP), Transformational/Early Neolithic (8300–7900 calBP) and Middle Neolithic (7900-7500 calBP) [42,46]. The occupants relied on hunting, mostly deer, and fishing for large sturgeon, carp and catfish. A significant degree of sedentism in the Late Mesolithic is inferred based on the seasonal schedule of food resource acquisition [47]. The presence of dogs is attested to (morphometric analysis) from the Late Mesolithic phase onwards. Domestic livestock appeared after the Transformational/Early Neolithic phase in which other Neolithic elements, such as pottery, "Balkan" flint and polished stone artefacts were gradually introduced [48].

More recent excavations at Vlasac (2006-2009), performed at an area of 326 m2, allowed for better understanding of the formation processes and stratigraphy and more detailed contextual research [49]. Important data about burial practices were obtained based on both primary and secondary inhumations containing at least 16 individuals, as well as at least seven cremation burials. Two features that were probably remains of dwellings are identified. Radiometric dating confirmed the occupation of the site from the last century of the 8th millennium B.C. to the first century of the 6th millennium B.C. The faunal composition is similar to the assemblage recovered in 1970–1971, but dog remains are more numerous, possibly related to the peripheral position of the portion of the settlement excavated in 2006-2009. The share of fish in the diet was significant, with Isotopic signals of the diet based on bones from five dogs showing similar carbon and nitrogen values as

freshwater and anadromous fish possibly indicating the consumption of fish as human food leftovers [50] or direct provisioning.

Genetic data retrieved from 17 humans interred at the site are shown a genetic ancestry most similar to that carried by humans of the Epigravettian and other Mesolithic individuals from across Serbia [51], with elevated patterns of drift with a 16,000 year old individual from Pınarbaşı in Türkiye over Neolithic genomes from the same region [52]. These two individuals from Vlasac are determined to carry “Iron Gates” ancestry, indicative of a largely homogenous population in Serbia and the surrounding regions between 10,500 and 9,000 years ago.

Heavily fragmented animal remains were found in the Mesolithic and Neolithic horizons that delivered human material, and included terrestrial mammals, fish, birds, amphibians and reptiles. *Canis lupus* (MNI: 27) material is found, with morphometric analysis (tooth crowding and jaw shortening) indicating a likely domestic origin for the majority of these Canids (*Canis familiaris*; MNI: 20; [49]). Cut marks and evidence of burning postmortem are also found on these dog remains, indicative of defleshing by humans, with dietary isotope data showing ð15N enrichment indicative of fish consumption [53].

For this study, a single dog specimen was incorporated, a fragmented left mandible with m1 and m2 in-situ **[inv.no VL 224 x-find 5/1]**. It was found in yellowish-brown scree deposits in the trench 3/2007, in the zone characterized by exclusively Late Mesolithic, mid-7th millennium B.C. burials ([49], Fig.4).

Padina, Serbia

Padina, like Vlasac, is an archaeological site on the right bank of the Danube, in modern day Serbia, located around 10 km upstream from the sites of Vlasac and Lepenski Vir [42,54,55]. It was excavated in 1968-1970, on an area of approximately 1100 m2, revealing Mesolithic hearths and at least 21 trapezoidal dwellings. Radiocarbon dating (four charcoal and 23 AMS - 13 human and 10 animal bones) indicate occupancy over a period of 4 thousand years (the second half of 12th to mid-8th millennium calBP).

Thirty seven skeletons were excavated from burial context at the site, with the majority in a poor state of preservation [56]. Genetic data from 13 individuals from the Mesolithic and Neolithic transitory period (max. 11200 calBP [AA-57771] to min. 7890 calBP [AA-57770]) show a similar genetic ancestry to that at Vlasac, with the majority of individuals carrying indicative “Iron Gates” genetic ancestry seen throughout the region during this period [51], albeit with two individuals showing evidence of elevated drift with Mesolithic populations from Ukraine indicative of EHG ancestry.

Animal remains from the site are numerous, with 4518 specimens of mammals, birds, fish, reptiles, and molluscs identified to a species or genus level. Among 281 remains of domestic mammals, 222 originated from dogs (79%). The majority of dog bones were associated with trapezoidal dwellings, i.e. to the Transformational/Early Neolithic phase.

Morphometric analysis of canid cranial remains from Padina, including 3 upper, 42 lower jaws and three isolated carnassials, and comparison to recent wolf populations from the region indicated that dogs were morphologically and by their size distinct from wolves. However, two fragmented mandibles and one isolated lower carnassial showed mixed characteristics of dogs and wolves and are determined as *Canis* sp.

A single specimen is incorporated into this study – a mandible fragment with p1–p4 alveoli that exhibits a size closer to wolves than dogs **[PAD 11.70/170/1]**[57] (Supplementary Table 1). Premolars' alveoli are tightly spaced, and p2 is rotated in this specimen, which are features more often found in dogs. The specimen is found in the context attributed to the Early/Middle Mesolithic (Sector II, trench 2, Block 52). The second specimen sampled for this study is an almost complete right mandible lacking only its oral incisive portion and with a slightly damaged coronoid process, but lacking also most of the teeth except for the p2 and broken canine **[PAD 7.70/224/1]**. By its size and morphology, it is attributed to a dog, while the context from which it originates corresponds to the Transformational/Early Neolithic age (Sector III, profile 3, Segment 1, House 7).

2. Radiocarbon Dating

We generated eight new radiocarbon dates for canids from Gough's Cave (n = 2), Pınarbaşı (n = 4), and Wezmeh Cave (n = 2) (Figure 2B; Supplementary Figure 3). Collagen extraction and AMS dating was conducted at either the Oxford Radiocarbon Accelerator Unit (Oxford, UK), or the University of Vienna (Vienna, Austria) using ~500mg of either tooth or bone powder/fragments.

University of Vienna, Austria

The samples of bone for AMS radiocarbon dating were drilled using clean tungsten carbide drill bits and analysed at the Higham Lab, Faculty of Life Sciences, University of Vienna (Austria). The samples weighed between 475-522 mg. Collagen was extracted using a modified Longin collagen method outlined in [58] and [59] The samples were gelatinised in weakly acidic pH3 water and ultra-filtered using pre-cleaned 30kDa Sartorius ultrafilters, before being freeze-dried and weighed. A 1 mg portion of each sample was measured for $\delta^{13}C$ and $\delta^{15}N$ values using an Elemental Analyser/Isotope Ratio Mass Spectrometer (EA-IRMS) to a precision of ±0.3 per mil relative to VPDB and AIR respectively. The C/N_{atomic} ratios generated are within the acceptable range (2.9-3.5) in our laboratory. All other parameters were acceptable. The remaining collagen was combusted, graphitised and measured at the Keck AMS Facility, University of California at Irvine (USA). Specifics of target preparation and AMS measurement can be found in [60] and [61]. The fraction modern values were corrected using blanks prepared in Vienna from a beyond radiocarbon background (the Hollis mammoth bone) which yielded values of 0.0022 and 0.0024 (±0.0001), or 49.1 and 48.5 ka BP. We dated in parallel a sample of VIRI I whale bone which has an international consensus value of 8331±6 BP. Our result was 8380±20 BP, in close agreement with the calibrated ages when we compare them.

Oxford Radiocarbon Accelerator Unit, UK

Samples were cleaned of visible dirt and/or carbonate crusts using an aluminium oxide air abrasive. Collagen was isolated using a modified 'gelatinization method' (adapted after [62]), and ultrafiltered [59]. ~2 mg of purified collagen from each sample was weighed into a tin capsule, and combusted in a GSL elemental analyser. A small portion (~2%) of this gas was separated via an open split and used for isotope ratio mass spectrometry (IRMS), however, these values were not reported (see below). The remaining gas was cryogenically collected, and reacted with hydrogen over an iron catalyst to produce graphite for radiocarbon dating. AMS measurements were undertaken on an IonPlus MICADAS accelerator mass spectrometer (AMS). Conventional Radiocarbon Dates (BP) were then calculated following [63], and corrected using AMS-derived $\delta^{13}C$ for carbon added during pre-treatment, with measurement uncertainty calculated following [64].

3. Stable Isotope Analysis

Isotopic analysis of $\delta^{13}C$ and $\delta^{15}N$ was undertaken at BioArCh, Department of Archaeology, University of York (UK). Collagen extraction of bone powder/fragments followed a modified [62] protocol with the addition of Ezee™ filters [65]. Briefly, samples were manually cleaned using a sterile scalpel to remove contamination, and demineralised in 8ml 0.6M aq. HCl at 4℃. Samples were rinsed to neutrality using deionised water and the resulting insoluble fraction gelatinised in 8ml pH3 HCl for 48h at 80℃, before being Ezee™ filtered to remove unwanted particulate matter. The resulting retentate was then frozen at -20℃ and lyophilized.

Lyophilized collagen samples (c.1mg) were analysed in duplicate by Elemental Analysis-Isotope Ratio Mass Spectrometry (EA-IRMS) on a Sercon HS2022 continuous flow isotope ratio mass spectrometer at BioArCh, University of York. Stable carbon and nitrogen isotope ratios were calibrated relative to the internationally defined standards of VPDB for $\delta^{13}C$ and AIR for $\delta^{15}N$, using the standard reference materials IsoAnalytical Cane (δ13C = -11.64‰ ± 0.03), Sigma Methionine (δ13C = -35.83‰ ± 0.03, δ15N = -0.76‰ ± 0.05) and IAEA N2 (δ15N = 20.41‰ ± 0.12). Uncertainty was monitored using the standard reference materials Sigma fish gel (δ13C = -15.27‰ ± 0.04, δ15N = 15.21‰ ± 0.13), IsoAnalytical Alanine (δ13C = -23.33‰ ± 0.10, δ15N = -5.56‰ ± 0.14) and IsoAnalytical Soy (δ13C = -25.22‰ ± 0.03, δ15N = 0.99‰ ± 0.07). Collagen quality fell within prescribed quality ranges [66,67]. Collagen yields (wt.%) were calculated using the formula [(bone mass (mg)/bone collagen mass(mg)) × 100].

Compound-specific isotope analysis

Compound specific isotope analysis was also undertaken at BioArCh, Department of Archaeology, University of York. Collagen (2-4 mg) was hydrolysed by adding 50 µl of a Norleucine internal standard (Sigma; d13C and d15N values measured in house at BioArCh) to each sample and 200 µl of 6M HCl. Vials were flushed with N2 and capped tightly, followed by heating at 110°C (24 h). The hydrolysate was then transferred to a nano-sep filter (0.45 um; PALL Life Sciences P/N ODM45C34) and centrifuged at 10000 rpm (60 s). The filtrate was transferred to a clean tube and extracted (x3) with a mixture of *n*-hexane/DCM (3:2 v/v; 200 µl) to remove any contaminating lipids that were present in the original collagen. The remaining acid fraction containing hydrolysed amino acids was then blown down to dryness under a gentle stream of Nitrogen and stored frozen until required for derivatization.

Amino acids were then derivatized to form *N*-acetyl-*i*-propyl (NAIP) esters (55) following [68]. Briefly, isopropanol and acetyl chloride (1 ml; 4:1 v/v) were added to the dried samples, and the tubes were sealed and heated at 100°C (1 h). After 1 hour, sample mixtures were cooled (at −20°C), and the solution was dried under a gentle stream of N_2 at room temperature. Dichloromethane (DCM) was added (2 × 0.5 ml) and blown down under a gentle stream of N_2 to remove excess reagents. Next, a mixture of acetic anhydride, triethylamine, and acetone (1 ml; 1:2:5, v/v/v) was added to the tubes and heated at 60°C

(10 min). The mixture was cooled (at −20°C) and then evaporated to dryness under a gentle stream of N_2 at room temperature. NAIP esters were then dissolved in ethyl acetate (EtAc; 2 ml). A saturated NaCl solution (1 ml) was added to separate polar and/or inorganic components, and the organic phase was transferred into a new culture tube. The phase separation was repeated with additional EtAc (1 ml). Trace amounts of water were removed from the organic phase with molecular sieves (sodium aluminium silicate, 0.3 nm: Merck KGaA, Darmstadt, Germany). The EtAc containing the NAIP esters was blown down under a gentle stream of N_2, and then DCM (0.5 ml) was added and dried to remove excess water. Samples were redissolved in known quantities of EtAc and stored at −20°C until required for analysis by GC-C-IRMS. The same derivatization procedure was used for preparing mixtures of international reference standards (Indiana, USA and SHOKO Science, Japan) and standards purchased from Sigma-Aldrich (Sigma-Aldrich Company Ltd., UK).

The GC-C-IRMS measurements of the AAs were conducted using a Delta V Plus IRMS (Thermo Fisher Scientific, Bremen, Germany) linked to a Trace Ultra 1310 gas chromatograph (Thermo Fisher Scientific, Bremen, Germany) with a GC IsoLink II interface fitted with a Cu/Ni combustion reactor maintained at 1000°C. Ultrahigh-purity–grade helium with a flow rate of 1.4 ml min−1 was used as the carrier gas. Ethyl acetate was used to dilute the samples, and 1 µl of each sample and 2 µl of each standard were injected at 240°C with a 3.5 s pre-injection dwell time onto a custom DB-35 fused silica column (60 m × 0.32 mm × 0.50 µm; Agilent J&W Scientific Technologies, Folsom, CA, USA). All samples were injected in triplicate. The oven temperature program used for samples and standards was as follows: 40°C (hold 5 min) and then increasing by 15°C min−1 up to 120°C, then by 3°C min−1 up to 180°C, then by 1.5°C min−1 up to 210°C, then by 5°C min−1 up to 280°C (hold 8 min). A Nafion membrane removed water, and a cryogenic trap was used to remove CO_2 from the oxidised and reduced sample when operated in nitrogen mode. In carbon mode, eluted products were combusted to CO_2 and ionised in a mass spectrometer by electron impact. Ion intensities of mass/charge ratio (m/z) 44, 45, and 46 were monitored to automatically compute the ^{13}C:^{12}C ratio of each peak in the samples. In nitrogen mode, ion intensities of m/z 28, 29, and 30 were monitored to automatically compute the ^{15}N:^{14}N ratio of each peak in the samples. Measurements were made with Isodat (version 3.0; Thermo Fisher Scientific) and were based on comparisons with a repeatedly measured high-purity standard reference gas (CO_2 or N_2). Further workup of the Carbon data was carried out using IonOS or Lyticos software (Elementar). The results from the analysis are reported in parts per mil (‰) relative to international standards using the δ notation.

$\delta^{15}N$ measurements of AAs

The $\delta^{15}N$ values reported in the present study are the mean of triplicate $\delta^{15}N$ measurements for each sample. An AA international standard mixture of known isotopic composition was run multiple times before the first sample to check for consistency and then after triplicate sample injections to monitor instrument performance and drift. The AA

standard mixture used for $\delta^{15}N$ determinations comprised eight international standards (Indiana and SHOKO Science) and L-norleucine (Sigma-Aldrich). $\delta^{15}N$ true value of L-norleucine was determined in-house by EA-IRMS. International standard reported values are as follows: Ala, +43.25 ± 0.07‰; Gly, +1.76 ± 0.06‰; Val, −5.21 ± 0.05‰; Leu, 6.22‰; Nle, +14.31 ± 0.08‰; Asp, 35.2‰; Glu, −4.52 ± 0.06‰; Hyp, −9.17‰; Phe, 1.70 ± 0.06‰. Sample $\delta^{15}N$ raw values were corrected via a calibration curve generated from mean raw values of the derivatized standards measured repeatedly throughout each run (Pearson R >0.999).

$\delta^{13}C$ measurements of AAs

The $\delta^{13}C$ values reported are a mean of triplicate $\delta^{13}C$ measurements for each sample. $\delta^{13}C$ AA measurements were then corrected by specific correction factors to account for the derivatising carbon and the kinetic isotope effect according to [69]. A standard AA mixture made up of 16 individual amino acids (purchased from Sigma-Aldrich, UK) was run multiple times before the first sample to check for consistency and then run after every three sample injections, and the average correction factors from the standard mixture were used for the correction of the samples. True $\delta^{13}C$ values of standards were measured at BioArCH via EA-IRMS. The $\delta^{13}C$ true values for each amino acid were: Ala, -19.31 ± 0.02‰; Gly, -33.31 ± 0.02‰; Val, -10.89 ± 0.02‰; Leu, -13.78 ± 0.06‰; Ile, -24.89 ± 0.07‰; Nle, -27.73 ± 0.08‰); Thr, -10.46 ± 0.01‰; Ser, -12.69 ± 0.09‰; Pro, -12.23 ± 0.02‰; Asp, -27.52 ± 0.12‰; Met, -29.88 ± 0.14‰; Glu, -28.57 ± 0.09‰; Hyp, -12.52 ± 0.03‰ ; Phe, -11.52 ± 0.05‰; Lys, -13.7 ± 0.11‰; Tyr, -24.85 ± 0.02‰.

Correction factors introduce a new source of error; therefore, the error propagated for each AA was calculated according to [69] incorporating measurement errors associated with the true standard values, in-run repeat measurements of derivatized standards, and the measured sample replicates.

Quality Control indicators

The relationship between proline and hydroxyproline stable isotope values is valuable tool to assess the quality of the analysis since hydroxylation of proline to form hydroxyproline via post-translational modification does not involve the exchange of nitrogen or carbon atoms and therefore the $\delta^{13}C$ and $\delta^{15}N$ values of both amino acids in collagen should be the same. The values are plotted in Supplementary Figure 9A-B and were highly correlated (Pearson R = 0.99 for $\delta^{13}C$, 0.98 and $\delta^{15}N$)

The AAs measured by GC-C-IRMS represent 90.4 % and 80.7 % of total carbon and nitrogen, respectively, in human collagen. We calculated the estimated bulk collagen $\delta^{13}C$ and $\delta^{15}N$ values by mass balance equations considering the relative contribution of each amino acid to collagen and compared the obtained values with those measured via EA-IRMS. Collagen sequences from humans (UniProt P02052, P02058)) to obtain AAs composition and the number of carbon and nitrogen atoms. The values are plotted in

Supplementary Figure 9C-D and were highly correlated (Pearson R = 0.94 for $\delta^{13}C$, 0.99 and $\delta^{15}N$).

4. Ancient DNA

Sampling

A total of 27 specimens were sampled from six archaeological sites across West Eurasia. For material from Gough's Cave, Wezmeh Cave, Grotta Continenza, Vlasac and Padina, ~500mg of bone or tooth was subsampled from each specimen using a Dremel multitool (with diamond saw/drill attachment). For material from Pınarbaşı, which was highly fragmentary, we selected elements with the most-dense cortical bone.

DNA Extraction, Library Preparation and Sequencing

Ancient DNA laboratory work was performed across dedicated clean-lab facilities in London (Ancient DNA Laboratory, Natural History Museum), Munich (Department of Veterinary Sciences, Ludwig Maximilian University of Munich) and Oxford (Research Laboratory for Archaeology and the History of Art, University of Oxford).

Natural History Museum (Gough's Cave)

DNA was extracted using a modified version after [9] of the Dabney DNA extraction protocol [70], with different processing steps for teeth and bone. Tooth fragments underwent an initial pulverisation step (45 seconds), followed by complete pulverisation of any remaining material. Powder was collected separately across each step, and subject to either a single ("interior portion" – second powder) or double ("exterior portion" – first powder) digestion [71]. Bone fragments were crushed using a sterilised mortar and pestle, with single digestion performed on the resulting bone powder. Extracts were eluted using 100 µL EB Buffer.

Double-stranded, dual-indexed DNA libraries were prepared from DNA extracts following a modified version of a double-stranded DNA library preparation [72] that incorporated column-based clean-up steps (20 PCR cycles). Amplified indexed libraries were quantified on an Agilent Tapestation 4200 using high-sensitivity D1000 reagents, and pooled in equimolar concentration for screening on multiple lanes of Illumina NovaSeq X Plus platform with paired-end (2x150BP) sequencing chemistry (Novogene, Cambridge).

For extracts that showed high levels of endogenous DNA preservation and complexity, and low levels of non-endogenous contamination, we generated additional double-stranded DNA libraries. Here, a UDG (USER) treatment step was implemented prior to the blunt-end repair step to improve data quality. USER-treated libraries were quantified as above and deep sequenced on multiple lanes of Illumina NovaSeq X Plus (150x2PE; Novogene Cambridge).

Ludwig Maximilian University, Munich (Pınarbaşı, Wezmeh Cave)

Bones and tooth roots were sectioned into fragments using a hand drill, pulverized using a mechanical press, and finely ground using a Retsch MM400 swing mill. Samples were subjected to a pre-lysis bleaching step [73,74], specifically a 15-minute incubation in 1mL

bleach (0.25% NaOCl), followed by two washes (vortexed and centrifuged at 3,000 rpm for 2 minutes) in 900µl HPLC-grade water, and one final wash in 700µl of 0.5M EDTA (pH 8.0). The powder was digested using 975µl of extraction buffer (0.45M EDTA, 0.05% Tween-20) and 25µl of Proteinase K (0.25mg/ml; Thermo Scientific™); incubated at 37°C for 48 hours, with a final 2-hour incubation at 56°C.

DNA extraction was performed semi-automatically using a Hamilton Microlab StarLet IV liquid handler (Hamilton, Germany) following a modified [75] protocol. Specifically, 150µl of lysate was combined with 1560µl of binding buffer (5M GuHCl, 40% 2-propanol, 0.12M sodium acetate, 0.05% Tween 20; [70]) and 10µl of washed silica-coated magnetic beads (G-Biosciences). After a 15-minute binding step, the beads were pelleted on a magnet, the supernatant discarded, and the beads washed three times with PE buffer (Qiagen) and air-dried for 3 minutes. DNA was then eluted twice by incubating 30µl of TET buffer (1mM EDTA, 10mM Tris-HCl, 0.05% Tween-20) at room temperature for 5-minutes, with eluate combined for a total extract volume of ~50µL. Two negative controls were included in each extraction round.

Double-stranded Illumina sequencing libraries were prepared from 32µl of each DNA extract using a modified [72] protocol. Blunt-end repair was performed by adding T4 DNA Ligase buffer, dNTPs, T4 PNK, and T4 DNA Polymerase, followed by incubation at 20°C for 30 minutes and 65°C for 30 minutes. Products were purified with silica-coated beads and washing buffer PB (Qiagen) with minor volume adjustments to the [76] protocol. After washing with PE buffer and air-drying, DNA was eluted with 21µl of TET buffer. Illumina P5 and P7 sequencing adapters were ligated by incubating 20µl of the purified blunt-end product with T4 DNA Ligase buffer, PEG-4000, T4 DNA Ligase, and adapters at 20°C for 30 minutes and 65°C for 10 minutes. Clean-up was performed on a Hamilton Microlab StarLet IV using Sera-Mag SpeedBeads™ (Cytivia) and 80% ethanol washes, followed by elution with 21µl of TET buffer. The libraries were finalized with a fill-in reaction using Isothermal amplification buffer, dNTPs, and BST 2.0 Polymerase, incubated at 65°C and 80°C for 20 minutes each.

Unique dual indices (UDIs) were added to 10µl (out of 30µl) of the library reaction for multiplex sequencing using AmpliTaq Gold™ DNA Polymerase. The amplification protocol included an initial denaturation at 95°C for 10 minutes, 15 cycles of 95°C for 30 seconds, 60°C for 30 seconds, and 72°C for 1 minute, followed by a final extension at 72°C for 7 minutes. Three library amplifications were obtained and subsequently pooled at equimolar concentrations for shotgun whole genome sequencing (PE, 150bp) on NovaSeq X Plus lane(s) (25B flow cell) at Macrogen, Amsterdam.

Oxford University (Padina, Vlasac, Grotta Continenza)

DNA was extracted from ~50mg of bone powder [70], and converted into double-stranded DNA libraries using the blunt-end single-tube (BEST) protocol [77]. Purified libraries were dual-indexed through the addition of unique 6 bp barcodes to 5’/3’ ends [72], with PCR

amplification optimised through qPCR to reduce clonality and maximise complexity. Concentration, and fragment size distribution of purified, amplified libraries were estimated on a Qubit™ 3 Fluorometer using high-sensitivity dsDNA standards, and an Agilent 2200 TapeStation using the high-sensitivity D1000 marker, respectively. Libraries were then pooled at equimolar concentration (5nM), and initially screened on an Illumina NextSeq 1000 at Genzentrum (Munich, Germany) using 60 bp paired-end sequencing chemistry. Libraries with relatively high levels of endogenous DNA and complexity were then shallow sequenced on an Illumina NovaSeq X Plus (150x2PE) at MacroGen (Amsterdam).

To increase the utility of Palaeolithic/Mesolithic individuals with low endogenous DNA (n = 15), we performed mitochondrial capture enrichment (Supplementary Table 3). Specifically, libraries were pooled according to endogenous DNA content (while ensuring index compatibility), and captured using a custom MyBaits® Target Capture Kit (Arbor Biosciences) following the High Sensitivity v.5 protocol with the following modifications: only a single round of capture was performed, using 5.5 µL baits per pool; hybridization reactions were incubated at 60°C for 48 hours; bait-target hybrids were washed at 60°C; post-amplification cleanup was carried out using AMPure XP beads. Captured library pools were then sequenced on an Illumina HiSeqX Series at Macrogen (Amsterdam, Netherlands).

Raw Data Processing

Raw reads from newly sequenced and publicly available ancient canid genomes (Supplementary Table 1), were processed using nf-core/eager v.2.4.6 [78]. We utilised the following EAGER workflow: pre-processing (FastQC v.0.11.9, AdapterRemoval2 v.2.3.2); mapping (BWA *aln* v.0.7.17-r1188; RL≥30; map. Q≥20), duplicate removal (dedup) and post-processing statistics (samtools v.1.12); evaluation of ancient DNA, and alignment quality (DamageProfiler v.0.4.9, Qualimap v.2.2.2-dev; [79]); and estimation of library complexity (preseq v.3.1.1); with a modified version of *CanFam3.1* including the Y-chromosome used as the reference.

CanID Taxonomic Identification

To determine the taxonomic status of all canid samples from which we generated low-pass screening data (n = 24), we utilised the canid identification (CanID) pipeline (https://github.com/lachiescarsbrook/CanID). This workflow generates pseudohaploid genotype calls at sites present in a reference panel of modern dogs and wolves (~2-million diagnostic transversions), and uses both projection-based Principal Component (PC) analysis and Linear Discriminant Analysis (LDA) to classify individuals as either dogs or wolves. With as few as 175 SNPs, this method can accurately (95-100% success rate) distinguish most ancient dog and wolf ancestries (excluding Pleistocene wolves, which require 275 SNPs for the same accuracy). 21 out of 24 samples yielded enough SNPs for accurate classification (between 267–54,013 SNPs), with five wolves and 16 dogs identified (Extended Figure 1). This included three wolves, and four dogs from Gough's

Cave, with one of the dogs directly dated to within the Magdalenian occupation layer (D_GoughsCave2_14604: 1,363 SNPs); four dogs from Pınarbaşı; and a wolf from Grotta Continenza (W_GrottaContinenza1_c12000: 267 SNPs). Of the two samples with too few SNPs for CanID classification, D_Padina1_11500-8300 was deep sequenced and identified as a dog, and D_Vlasac2_c8650 shared maternal affinities with dogs at the same site (see below).

Genotype Calling and Merging

We incorporated ancient samples into an existing dataset containing over 1,700 modern canid genomes, including 1,573 dogs and 122 wolves, which have been previously used for population genomic analyses of ancient canids [80]. To minimise the impact of ancient DNA damage on downstream analyses (i.e. C-to-T and G-to-A substitutions), non-biallelic markers (-v snps), and transitions (-m1 -M2) were filtered out using the view function in bcftools [81]. We generated pseudo-haploid genotype calls for all modern genomes by randomly sampling an allele at each variant to match the pseudohaploid call of the ancient genome (see below). This resulted in 7,454,020 biallelic transversions.

For ancient individuals, genotypes were called at each biallelic SNP by randomly sampling a single read (≥30 bp), with mapping (MAQ) and base (BQ) quality scores of at least 20, excluding bases within 5 bp of the start/end of each read, and reads shorter than 30bp in length, using ANGSD. For each ancient canid, every position where the genotype did not match with the major or minor allele in the modern reference panel was marked as missing.

Mitochondrial Phylogenetics and Molecular Dating

Majority consensus (75%) mitochondrial genomes were called at all sites covered by at-least three reads (-*d* 3) using samtools *consensus* v.1.16.1 [82], with missing sites retained for alignment. We also downloaded 220 publicly available mitogenomes from NCBI, representing the majority of ancient and modern dog and wolf diversity, including previously-published 15 Palaeolithic canids, as well as the coyote reference (NC_008093) for use as an outgroup. To minimise gaps, and improve the overall alignment accuracy, we removed the hypervariable control-region (15458–16727bp) from each sequence. Truncated sequences were aligned using MAFFT v7.505 [83] with default parameters (--*auto*).

We then constructed phylogenies from the mitochondrial alignment (15,457 sites across 244 dogs and wolves). First, we constructed a maximum-likelihood tree in IQ-TREE v.2.1.4 [84] from 1,000 ultrafast bootstrap replicates (-*B* 1000), with the best-fit substitution model (HKY+F+I+G4) selected automatically using ModelFinder (-*m* TEST; [85]), and the coyote specified as the outgroup (-*o*) (Figure 1D).

To calculate the time-to-most-recent-common-ancestor of Palaeolithic canids, we constructed a time-calibrated Bayesian phylogeny in BEAST2 v.2.6.7 [86], which was

calibrated using the mean estimates of direct radiocarbon dates (Supplementary Table 3). The other priors implemented include the relaxed clock exponential with a mean in real space of 1.0 x 10-8, an upper bound of 1.0 x 10-6 substitutions/site/year, and a lower bound of 1.0 x 10-9 substitutions/site/year (with the bounds as part of a separate uniform prior). To determine the best fit clock model for our dataset, we nested sampling analyses comparing the strict clock vs the relaxed clock exponential using the NS package implemented in BEAUTi2 [87]. The Marginal Likelihood value of the strict clock (-35611.4; SD=11.4) is substantially larger than the Marginal Likelihood value of the relaxed clock exponential (-35426.9; SD=11.9), indicating that the relaxed clock exponential is a more favourable clock model for our dataset.

Next, the HKY+Γ4 substitution model was used, and for the tree prior we used the constant coalescent population model. A lognormal prior for kappa and an exponential prior was selected for the gamma shape prior, with default parameters. For all other parameters, default settings were used. Posterior distributions of parameters were estimated by Markov chain Monte Carlo (MCMC) sampling. Samples were drawn every 10,000 steps over a total of at least 1 billion steps. The first 15% of samples were discarded as burn-in. Sampling was considered sufficient when the effective sample size of each parameter exceeded 100. The trace files were assessed using Tracer [88] and samples from two independent runs were merged using LogCombiner.

Finally, we estimated ages of samples without direct radiocarbon dates (i.e. Mesolithic Serbian dogs; Supplementary Table 4) using CanDate (https://github.com/mahaut-goor/CanDate-repo), a tool which performs semi-automated tip-dating in BEAST2 using a reference database (n = 192) of modern and directly-dated ancient canid mitochondrial genomes. For unknown age 'target' samples the tip-date is set to 0 with a monophyletic MRCA prior of uniform distribution ranging from 0–100,000, whereas, for known age 'reference' samples, a fixed tip-date corresponding to the mean calibrated radiocarbon age is set. CanDate then performs four independent Markov chain Monte Carlo (MCMC) runs of 20 million generations each in BEAST2 using the following parameters: HKY substitution model, with estimation of the transition/transversion ratio (κ) and base frequencies; fixed κ value of 27 (mean) and one gamma rate category; strict molecular clock model with a lower rate bound of 1.0×10^{-8}, an upper bound of 1.0×10^{-6}, and a mean rate of 7.0×10^{-8} substitutions per site per year; and a coalescent Bayesian Skyline prior for the tree model. All other parameters are kept at their default settings. Chains were rerun if the ESS for any parameters failed to exceed 200.

Population Structure and Admixture

We performed Principal Component (PC) analysis on the entire pseudo-haploid dataset (7,454,020 biallelic variants across 426 dogs and wolves) using *smartpca* (EIGENSOFT v.8.0.0; [89]), excluding all outgroup taxa, as well as North American wolves given their ancestry contributions from both Pleistocene wolves and coyotes. Due to the presence of DNA damage and missing data, ancient samples were not used in the construction of

eigenvectors and were instead projected into PC space through a least-squares approach (lsqproject: YES).

We also performed PCA in EMU v.0.9 [90], which utilises expectation-maximisation to "impute" missing values in low-coverage genomes and enables their use in eigenvector construction. We ran EMU on a subset of the pseudo-haploid dataset that contained exclusively ancient dogs and wolves (7,454,020 biallelic variants across 146 individuals – "ancient-only"), calculating the first four eigenvectors (-e 4). These two methods (smartpca and EMU) produced equivalent results (Figure 1C; Supplementary Figure 4).

Shared drift was computed between all ancient and modern dogs and wolves using outgroup-f3 comparisons of the form f3(X, Y; Coyote) as implemented in the *Calc-f3* function in struct-f4 [91]. These were computed for every possible combination (excluding outgroup taxa) and pairwise f3 statistics were converted into a symmetric matrix using a custom script (Extended Figures 3–4; Supplementary Figure 18). We then visualised spatial and temporal patterns in these relationships by comparing genetic distance against the geographic distance between the archaeological sites under comparison. To do this, we computed great-circles (Haversine) distances as follows:

$$a = sin^2\left(\frac{dlat}{2}\right) + cos(lat1) \times cos(lat2) \times sin^2\left(\frac{dlon}{2}\right)$$

$$c = 2 \times atan2\left(\sqrt{a}, \sqrt{1-a}\right)$$

$$d = R \times c$$

Where: a = uses spherical geometry to compare differences in latitude and longitude, c = computes the central angle between the two points, d = calculates the distance between two points, and R = the radius of the earth (6,371km). We excluded ancient dogs from North America, given their erroneously short geographic distance to sites in Western Europe. We found genetic distance was strongly negatively correlated with geographic distance under linear regression in R ($R^2 = 0.490$, $F_{1,125} = 121.9$, p-value < 0.001). To detect outliers, we first calculated the distance of each point to the trend line, and then used these values to compute Z-scores for each individual comparison.

We applied Multiple Regression on Distance Matrices (MRM; [92]) to determine the influence of spatial and temporal distance on pairwise outgroup-f3 values. MRM performs a multiple regression of a response matrix against one or more explanatory matrices, each representing pairwise distances or similarities among *n* subjects (e.g., ecological, spatial, or temporal attributes). Statistical significance is evaluated through permutation testing. Model fitting was conducted in R, using the MRM function implemented in the ecodist package. We included pairwise outgroup-f3 values for 63 ancient dog genomes (>0.1x) and applied a z-transformation to the spatial and temporal distance matrices,

standardising each to have a mean of zero and a standard deviation of one. This transformation accounts for differences in measurement scale and ensures that both predictors are directly comparable in terms of effect size. Standardisation is essential in distance-based multivariate analyses, as variables with larger numeric ranges can exert disproportionate influence on model estimation. Both spatial and temporal distance exert significant effects on outgroup-f3 values, with the influence of spatial distance being approximately twice as strong as that of temporal distance. In MRM models, residuals are not expected to be normally distributed because pairwise distances introduce statistical dependence among observations, violating the independence assumption of standard regression. Consequently, statistical inference is based on permutation testing rather than parametric assumptions. In our analysis, the residuals are approximately normally distributed, with only a few deviations attributable to outlying pairs (Extended Figure 3). The Palaeolithic dog pair (Gough's Cave and Pınarbaşı) does not exhibit abnormal behaviour (Extended Figure 3), as its residual lies within two standard deviations of the mean (approximately zero).

We performed model-based estimation of population structure using the algorithm implemented in ADMIXTURE v.1.3.0 [93]. We used the "ancient-only" dataset, and ran ADMIXTURE using two ancestral components, with 50 bootstrap replicates (-B 50) performed for each run to obtain global cross-validation errors (--cv) (Supplementary Figure 5).

We also performed supervised ADMIXTURE analysis (--supervised) to assess the levels of excess Near Eastern wolf ancestry in ancient West Eurasian dogs (Supplementary Figure 14). Individuals were modelled using two ancestral components, with the following used as sources: East Eurasian dogs (D_Khatystyr1_8645, D_PortauChoix1_4157, D_ShamankaII1_7400, D_Zhokhov1_9515, D_PadKalashkinova2_6900) and Near Eastern wolves (grey: Wolf07, Wolf20, Wolf55, Wolf56, Wolf70, Wolf71, Wolf72, W_Wezmeh1_2708) across 100 bootstrap replicates (-B 100).

Genetic similarity and relatedness

We measured genetic kinship coefficients (θ) using READv2 [94], with default normalisation (median) and block jackknife window-size (5000000) parameters [95]. To reduce the impact of DNA damage, we excluded variants that were only identified within a single individual using a minor allele frequency cut-off (--maf 0.02) in PLINK v.1.90b6.21. We then performed pairwise kinship analysis on all possible pairwise combinations of ancient European (n = 31) and Near Eastern (n = 12) dogs. As these individuals represent two different populations, we normalized P0 values with two different medians. Specifically, we used the median of all European-European (normalized P0 median: 0.077) or Near Eastern-Near Eastern (Normalized P0 median: 0.082) population pairs with common SNP counts higher than 10,000, respectively. The validity of these normalisations was then assessed by measuring the correlations between the estimated θ of renormalized P0 values of each pair, and their respective outgroup-f3 values in the form of f3(X, Y; Coyote)

(Figure 2A; Supplementary Figure 7). We observed high correlation between these two estimates (Spearman correlation coefficient: r = 0.86, p < 0.001) for both normalisation values.

Dog-Human Ancestry Comparison

We sought to investigate the shared evolutionary histories of humans and dogs across Eurasia and the Americas over the last 16,000 years. First, we performed kinship analysis on ancient humans from Europe (n = 21) and the Near East (n = 11), who were either recovered from the same archaeological contexts as dogs, or were associated with the same culture and found in close geographic (<1 degree) and temporal (+-500 years) proximity. Pseudohaploid genotype data for ancient humans at 1240k SNPs were collated from the Allen Ancient DNA Resource v64 (AADR; [96]). We used the median of all European-European (normalized P0 median: 0.256) or Near Eastern-Near Eastern (Normalized P0 median: 0.263) population pairs with common SNP counts higher than 10,000, respectively. We then tested whether relatedness in both humans and dogs followed a simple isolation-by-distance model by calculating the geographic distance between the archaeological sites using Haversine distances. We found kinship was highly linearly correlated with geographic distance under regression performed using the *lm()* function in R for both dogs ($R^2 = 0.356$, $F_{1,859} = 476.2$, p-value << 0.001) and humans ($R^2 = 0.379$, $F_{1,701} = 429.4$, p-value << 0.001), which is expected under an isolation-by-distance model (Supplementary Figure 8).

We extended this investigation to test for correlations in pairwise outgroup-f3 statistics from both humans and dogs found within the same archaeological context and period, or in close geographic and temporal proximity. A total of 35 suitable human-dog pairs were identified across Eurasia and the Americas and all possible pairwise outgroup-f3 values calculated (Supplementary Table 7). We conduct a partial Mantel test (using R *ecodist*) to evaluate the correlation between the dog and human outgroup-f3 distance matrices while controlling for spatial and temporal distance. We find a positive correlation (Mantel $r \approx 0.40$, $p < 0.0002$), indicating a statistically significant association between the two matrices beyond spatial and temporal effects (Extended Figure 3C).

To determine asymmetrical relationships (where a pair of individuals from two archaeological sites share substantially more or less drift compared with the other species from the same sites), we examined distribution and differences in human and dog outgroup-f3 values. We standardised f3-outgroup matrices to have zero mean and unit variance prior to comparison. Several pairs exhibit values exceeding two standard deviations from the mean (Extended Figure 3D), indicative of unusually large discrepancies between dog and human outgroup-f3 similarities. The Palaeolithic human-dog pair [Gough's Cave and Pınarbaşı] are within the first quartile of the distribution, indicating the dogs are more similar to each other than expected given the human relationships (i.e. Magdalenians and Anatolian Hunter-Gatherers; Extended Figure 3D).

AMY2B Gene Copy Number Estimation

To estimate the copy number of the AMY2B gene, we followed the approach outlined in [97] with slight modifications. Specifically, we first used samtools *view* to calculate read coverage across four *AMY2B*-related regions (with a total region size of ~73 kb) of the CanFam3.1 reference genome (chr6:46948800-46956325, chrUn_AAEX03020568, chrUn_AAEX03022739, and chrUn_AAEX03024353), as well as 73 randomly chosen 1kb windows from across the rest of the genome (chr1-38), for all ancient dogs and wolves in our dataset. We then calculated the ratio of reads mapping in amylase/non-amylase regions, and computed confidence intervals (CI) using normal approximation as follows: 1.96 × AMprop × (1 − AMprop)/(amylase reads + non-amylase reads), where AMprop is the proportion of reads in amylase regions. Finally, we converted these ratios to estimates of diploid copy number through multiplication with a scaling factor, which was calculated as the mean value required to scale all wolves to two copies (assuming Eurasian gray wolves have two copies, excluding outlier individuals; Supplementary Figure 10).

D-statistics

We calculated D-statistics using the *qpDstat* function implemented in AdmixTools v.7.0.2 [98]. We treated all individuals as inbred (*inbreed:* YES) to account for pseudohaploidization. Standard errors were estimated by performing weighted block jackknife over 5Mb blocks, and used to calculate Z-scores. To visualise the diversity of ancient dogs (X) with respect to East and West Eurasian dog and wolf ancestries [99] we performed D-statistics of the form: D(Coyote, X, W_Wezmeh1_2708, W_BeleyaGora1_18148) and D(Coyote, X, D_TepeGhelaGap1_5826, D_Zhokhov1_9515) (Supplementary Figure 11). We also used D-statistics of the form: D(Coyote, Near Eastern wolf, X, D_Pinarbasi1_15787 / D_Zhokhov1_9515), and D(Coyote, D_Zhokhov1_9515 / W_Wezmeh1_2708, X, D_Pinarbasi1_15787) to test for gene flow between West Eurasian dogs (X) and Near Eastern wolves (Extended Figure 5, Supplementary Figures 12–13).

TreeMix

We constructed admixture models using TreeMix v.1.13 [100] and AdmixtureBayes [101] with representatives from East (D_Zhokhov1_9515, D_PadKalashkinova2_6900) and West (D_Pinarbasi1_15787, D_GoughsCave1_14269, D_TepeGhelaGap1_5826, Basenji) Eurasian dog lineages, an ancient Iranian wolf (W_Wezmeh1_2708) representative of Near Eastern wolf ancestry, and Pleistocene East (W_BeleyaGora1_18148) and West (W_HoehleFels5_32366) Eurasian wolves. Allele frequencies (*--freq*) were first calculated for these populations using PLINK (--within), with the gunzipped output files converted into TreeMix format using the *plink2treemix.py* script (https://github.com/thomnelson/tools/blob/master/plink2treemix.py). We first estimated a maximum-likelihood (ML) tree without admixture, and then added 1–4 migration edges (-*m*), with all trees rooted against the coyote (Extended Figure 7). Block-size for covariance

estimation was set to 100 SNPs (*-k*), with a total of 1,000 bootstrap replicates (*-bootstrap*) performed (Supplementary Figure 15).

AdmixtureBayes

AdmixtureBayes [101] was run using the same input as TreeMix for 1 to 4 admixture events (--max_admixes 1 to 4). Each was run in triplicate for one million generations (--n 1,000,000) using eight Markov-Chain Monte-Carlo (MCMC) chains (--MCMC_chains 8). We checked for convergence of the MCMC sampler (using EvaluateConvergence.R) before constructing trees (--plot estimates; --plot consensus_trees; --plot treestop_minimal_topologies; --plot estimates) and determining posterior probabilities of both nodes and complete tree.

F4-ratio

To quantify the proportion of Near Eastern wolf ancestry in both ancient Near Eastern dogs and the Basenji (X), we performed F4-ratio estimation using AdmixTools (qpF4ratio) with the following setup:

$$\alpha = \frac{F4(D\_Zhokhov1\_9515, Coyote;\ X, W\_Wezmeh1\_2708)}{F4(D\_Zhokhov1\_9515, Coyote;\ D\_Pinarbasi1\_15787, W\_Wezmeh1\_2708)}$$

Jackknife block-size was set to 100 SNPs (*blgsize:* 0.01) by assigning false physical positions (i.e. increments of 10,000 bases) and genetic distances (0) to each site. Mean admixture proportions (α) and Z-scores were calculated for each Near Eastern dog, with only significant ($|Z|>3$) comparisons considered (Extended Figure 8B).

qpWave and *qpAdm*

qpWave and *qpAdm* analysis was performed using AdmixTools v.7.0.2 to test ancestry models for dog populations across Eurasia [98,102].

qpWave analysis was performed to test the cladidity of dog populations across Eurasia and evaluate the fit of models with a single ancestry source. First, we used Palaeolithic wolves as reference populations given (i) their likely asymmetrical relationships with dogs, and (ii) no chance of admixture between these wolves and dogs, before extending analysis post-hoc to test additional reference model combinations. We used two sets (small and large; following [99],), with results from the smaller set of reference population given more weight given a large set of reference populations can (i) lead to the covariance matrix of f4-statistics to be poorly estimated resulting in inaccurate computed p-values [103], and (ii) introduce artificially asymmetrical population histories between samples. Analysis was performed in AdmixTools v.7.0.2 [98], with option allsnps: YES used in all cases to maximise the number of SNPs used to calculate f4-statistics and thus improved power to reject models.

Reference populations (outgroup) used were as follows:

[Reference population set - large]: W_IndependenceCreek1_29143, W_Razboinichya2_28345, W_QuartzCreek1_29943, W_HoehleFels1_31364, W_BeleyaGora2_32020, W_HoehleFels5_32366, W_Letniaya1_32781, W_HoehleFels2_33163, W_Badyarikha2_33515, W_Ogorokha1_36050, W_Fairbanks4_37604, W_Fairbanks1_37616, W_ParadiseHill1_43821, W_Unnegen1_44450, W_BungeToll1_48210 and W_AufhausenerHoehle3_50238.

[Reference population set - small]: W_AufhausenerHoehle3_50238, W_HoehleFels5_32366, W_BungeToll1_48210, W_BeleyaGora2_32020, W_Razboinichya2_28345, and W_Unnegen1_44450

First, we confirmed that “Near Eastern” dogs (D_TepeGhelaGap1_5826, Basenji05, D_TelGezer1_3500; large: p=0.083; small: p=0.0949) and “Arctic” dogs (D_Zhokhov1_9515, D_PortauChoix1_4157, D_PadKalashkinova2_6900; large: p=0.392, small: p=0.165) could be modelled as single populations. A 10,900 year old dog from Veretye could be included in the Arctic dog clade using the large set of reference population (p=0.295) indicating shared ancestral history, although this reduction in p values, and the rejection of the model when using the small reference population group (p=0.019), indicates there may indeed be ancestry in this 10,900 year old Veretye individual was not present in other individuals in the clade. This conforms with results of D-statistics (e.g. D(Coyote, Veretye; Arctic/Upper Palaeo; Near Eastern and D(Coyote, Arctic; Veretye/Upper Palaeo; Near Eastern)), which shows Veretye and these “Arctic” dogs have differing affinities to other dog and wolf samples (Supplementary Figure 11–13**).** From here on, all reported p-values are those from the small set of reference populations unless otherwise stated.

We then assessed whether LUP dogs and Arctic/Near Eastern dogs can be modelled as a clade. To confirm the divergence of Arctic and Near Eastern dog populations, we combined these two ancestral groups, and attempted to model these individuals as a single clade as above. The model fit poorly (D_TepeGhelaGap1_5826, Basenji05, D_TelGezer1_3500, D_Zhokhov1_9515, D_PortauChoix1_4157, D_PadKalashkinova2_6900; p= 0.00135), with the sequential addition of NE dogs into the Arctic group showing an additional wave of admixture was required to model these groupings in all cases (p = 0.00476; 0.0122; 0.0357). This follows [99], and indicates asymmetrical population history. Palaeolithic dogs were sequentially appended to both Near Eastern and Arctic groups, and the cladidity of each group again tested. Gough’s Cave dog was weakly rejected as forming a clade with the Arctic set (large: p=0.079, small: p=0.0444), but could not be rejected as forming a clade with the Near Eastern (large: p=0.094, small: p= 0.551) group. The dog from Pınarbaşı could be modelled as forming a clade with Arctic (large: p=0.089, small: p=0.403), and Near Eastern (large: p=0.047, small: p=0.403) dogs.

Results of this analysis are difficult to interpret, given the contradictory nature of several of these outputs. The modelling of Pınarbaşı and Gough's Cave as cladal, as inferred from f4-statistics, is to be expected. However, the differing relationship of each to the

Arctic dog clade is not consistent with these results. Results may indicate that each Paleolithic dog has a differing relationship between these Arctic dogs in relation to the outgroup populations, that would result from asymmetrical ancestral histories of these dogs in relation to these outgroup wolves (i.e. asymmetrical gene flow). This is supported by f4-statistics that identified a small proportion of basal canid ancestry in the dog from Gough's Cave (Supplementary Figures 18-20).

Further, results suggesting that Near Eastern dogs and LUP dogs form a clade are inconsistent with results of f4-statistics and tree-based admixture graph analysis, which indicate that Near Eastern dogs possess ancestry from a local wolves (i.e. an ancient Iranian wolf, W_Wezmeh1_2708). These results may be explained by LUP and Near Eastern dogs being monophyletic with respect to the "outgroup" wolf genomes used in the *qpWave* analysis, which do not include any individual carrying indicative "Near Eastern" wolf ancestry .

We thus extend qpWave analysis, using the smaller reference set of Pleistocene wolves with the additional of a post Pleistocene wolf genome from the Near East (W_Wezmeh1_2708):

[Reference population set - Near East] = W_UlakhanSular1_16864, W_AufhausenerHoehle3_50238, W_HoehleFels5_32366, W_BungeToll1_48210, W_BeleyaGora2_32020, W_Razboinichya2_28345, W_QuartzCreek1_29943 and W_Wezmeh1_2708.

Near Eastern dogs could be modelled as forming a single clade (p=0.224), indicating shared ancestral history. However, we could reject all single source models that included any variation of Palaeolithic and Near East dogs. These results support f-statistics and tree-based methods that show Near Eastern dogs possess additional ancestry from Near Eastern wolves when compared to Palaeolithic dogs (Figure 3A; Extended Figure 7).

qpAdm was used to model the ancestry of both modern and ancient West Eurasian dogs using three potential source populations informed from f4-statistics and tree-based methods: D_Pinarbasi1_15787 (West Eurasian Palaeolithic); W_Wezmeh1_2708 (proxy for ancestry stemming from NE wolves); D_Zhokhov1_9515 (East Eurasian ancestry). To ensure these source populations were the best fit for non-West Eurasian dog ancestry, we calculated D-statistics of the form: D(Coyote, W_Wezmeh1_2708, X, D_Pinarbasi1_15787) and D(Coyote, D_Zhokhov1_9515, X, D_Pinarbasi1_15787) (Supplementary Figures 12-13). We used a set of wolves and dogs in the reference population, and ensure these included individuals that showed differential relationships to both source and target individuals following [104]:

[Reference population set - qpAdm] = W_BungeToll1_48210, W_Unnegen1_44450, W_BeleyaGora2_32020, W_Razboinichya1_81793, W_Razboinichya2_28345, D_PortauChoix1_4157, W_AufhausenerHoehle3_50238, Dingo03_35294 and Dhole_Hovk1_70000.

We implemented qpAdm using a wrapper script (https://github.com/pontussk/qpAdm_wrapper.git) that was modified to consider all canid autosomal chromosomes (chr1:38). For each tested model, source populations not considered were added to the reference population. We ranked models based on *P-values* (proportion) for single, dual and triplet sources, and rejected models (i) with *P-values* <0.01, or (ii) whose ancestry proportions contained negatives (i.e. impossible), with the source combination showing the highest *P-value* deemed the best fitting model for that individual. We used allsnps: YES for all qpAdm and qpWave analyses to maximise the number of SNPs used to calculate f4-statistics and thus improve power to reject models (Figure 3B; Extended Figure 9). Mean ancestry proportions for Mesolithic (13,000 to 9,000 years ago), Neolithic (9,000 to 5,200 years ago), Bronze Age (5,200 to 2,800 years ago), Iron Age (2,800 to 1,500 years ago), Medieval (1,500 to 500 years ago) and Modern period dogs were calculated using dogs where the *P*-value for a dual-source West Eurasian-East Eurasian (D_Pinarbasi1_15787 and D_Zhokhov1_9515) model was >0.01 (Supplementary Figure 21). After initial admixture between East and West Eurasian dog lineages in Mesolithic Europe (mirrored in human genomes), we observe an overall decrease in the proportion of East Eurasian ancestry in European dogs through to the Medieval period. This may represent a reduced influx of East Eurasian dog ancestry after the Mesolithic, an influx of dogs that carry West Eurasian dog ancestry (e.g. from the Near East), or the persistence of dog populations that carry West Eurasian dog ancestry from the Mesolithic onwards that are not represented in the current Mesolithic dataset.

Basal ancestry in Gough's Cave dog

Our analyses identified a basal ancestry contribution into the dog [M13794] from Gough's Cave (K = 2; Extended Figure 6). To determine the validity of this signal, we calculated outgroup-f3 statistics of the form: f3(Coyote, X, D_Pinarbasi1_15787/D_GoughsCave1_14269) and D-statistics of the form: D(Coyote, X, D_GoughsCave1_14269, D_Pinarbasi1_15787), where X was either ancient wolves or dogs, to test for asymmetry in allele sharing with Palaeolithic dogs (Supplementary Figures 18–20). We found both dogs and wolves (including extinct Pleistocene populations) showed closer affinities to the Pınarbaşı dog, which further supports the presence of an ancestry component basal to all known *Canis lupus* diversity in the Gough's Cave dog.

To ensure this signal was not the result of contamination, we ran each of the Gough's Cave samples sequencing libraries (both UDG and non-UDG treated) through Kraken2 using a large database of prokaryote and eukaryote genomes[105]. Several libraries showed an excess of reads that were assigned as human contamination (1:1 ratio of reads aligning to the human and dog genomes), primarily those with <0.5% endogenous DNA. These libraries, however, had not been included in the final D_GoughsCave1_14269 sequence alignment as they failed our initial quality control steps.

To ensure that low level contamination across other sequencing libraries was not the cause of this basal ancestry, we performed competitive mapping of all libraries to both the human and dog genomes (following the protocol outlined above in “Raw Data Processing” and “Genotype Calling and Merging” sections). We then calculated D-statistics of the form: D(Coyote, D_Pinarbasi1_15787; D_GoughsCave1_14269, D_GoughsCave1_14269_COMPMAP). No tests were significant (|Z|<3), which indicates that competitive mapping had not removed the basal ancestry, and thus that underlying contamination was not the driver of this signal. We conclude that this signal is genuine, although we lack the resolution to accurately determine the potential source. Given the presence of this unidentified ancestry component in D_GoughsCave1_14269, we solely use D_Pinarbasi1_15787 as the representative genome of Palaeolithic West Eurasian dog ancestry in all nuclear-based genomic analyses.

Outgroup Selection

We combined four coyotes as outgroups for our analyses. These individuals were selected based on their low level of detectable dog ancestry with respect to Coyote01, i.e. the coyote which share the lowest drift with dogs/wolves as measured by outgroup-f3 statistics of the form f3(BlackBackedJackal01, Coyote, Dog/Wolf)).

We then used a F4 ratio of the form:

$$\alpha = \frac{F4(W\_BeleyaGora1\_18148, BlackBackedJackal01;\ X, Coyote01)}{F4(W\_BeleyaGora1\_18148, BlackBackedJackal01;\ Y, Coyote01)}$$

where X represents coyotes with suspected dog ancestry, and Y represents the following diverse dog ancestries: Palaeolithic West Eurasian (D_Pinarbasi1_15787), Near Eastern (D_TepeGhelaGap1_5826), and East Eurasian (D_Zhokhov1_9515), to calculate excess dog ancestry in the other three coyotes used as outgroups. This test shows that all three coyotes possess marginal excesses of dog ancestry: Coyote02 (5.2–6.2%), Coyote05 (2.5–3.3%), and Coyote06 (2.8–3.6%).

To determine the impact of this low level dog ancestry on our analyses, specifically those testing for gene flow from Near Eastern wolves into dogs, we ran D-statistics of the form: D(Outgroup, Near Eastern Wolf, X, D_Pinarbasi1_15787), where "Outgroup" is either Coyote01, or all four coyotes (Supplementary Figure 21). We found no significant difference between the D-statistics (t = -0.26437, p-value = 0.7919) or Z-scores (t = 0.51575, p-value = 0.6069) between the two tests, which suggests the impact of low-level dog ancestry in our coyote outgroup is negligible.

We suspect that the low amount of dog ancestry in these coyotes has a minimal impact on our results because it is unlikely to affect allele polarisation, but rather lead to conflicting genotypes among coyotes which will lead to missing data.

5. Supplementary Figures

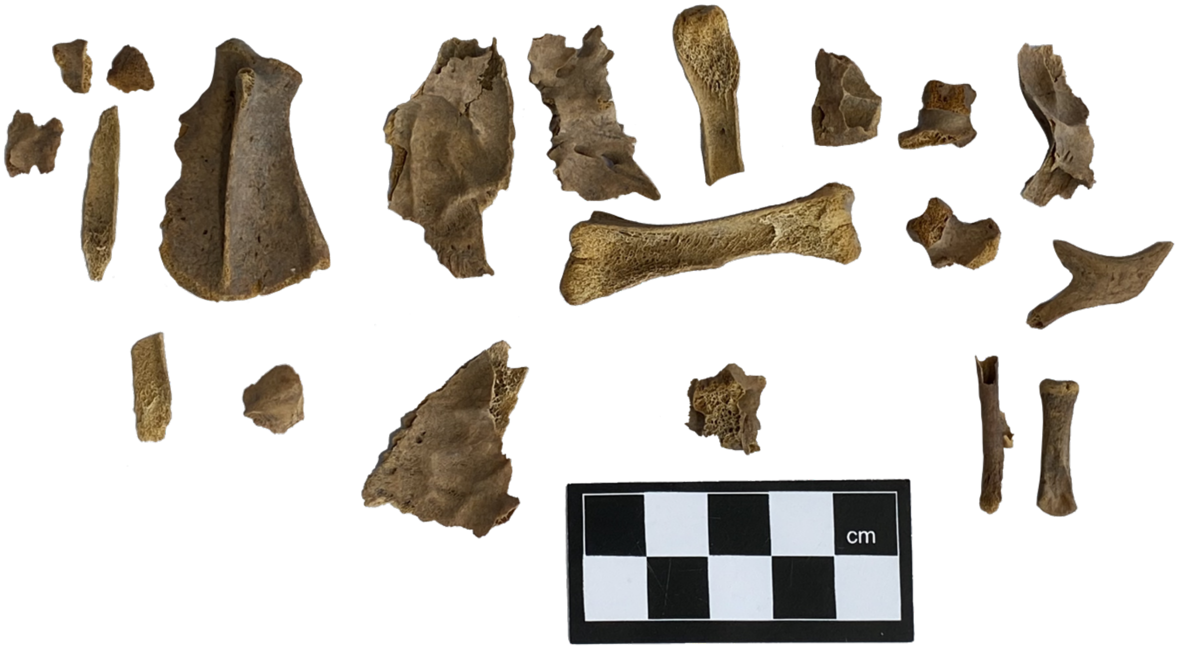

Supplementary Figure 1. Fragmentary perinatal-young dog remains recovered from Pınarbaşı (PB04 BIF 31/8/04). Photo © Lachie Scarsbrook.

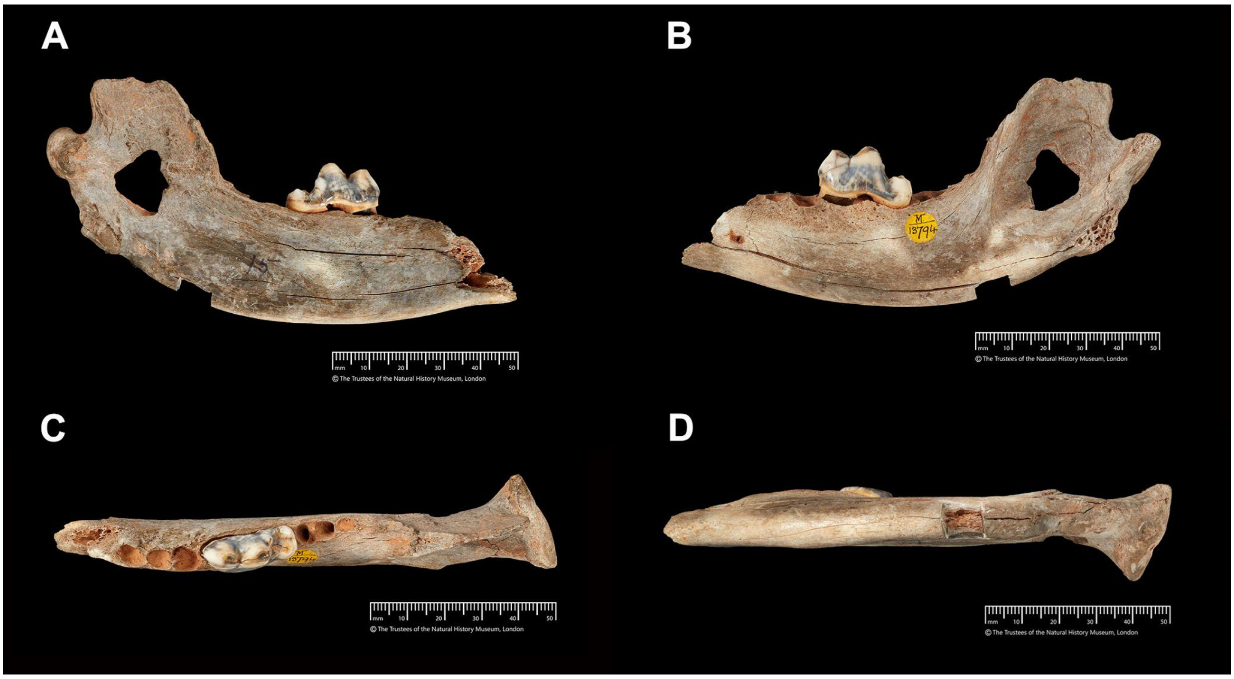

Supplementary Figure 2: Gough’s Cave specimen NHMUK PV M13794. A left canid mandible in **(A)** medial, **(B)** lateral, **(C)** superior, and **(D)** inferior view. Anthropic modification of the masseteric fossa can be seen in view A-B. Photo © The Trustees of the Natural History Museum, London.

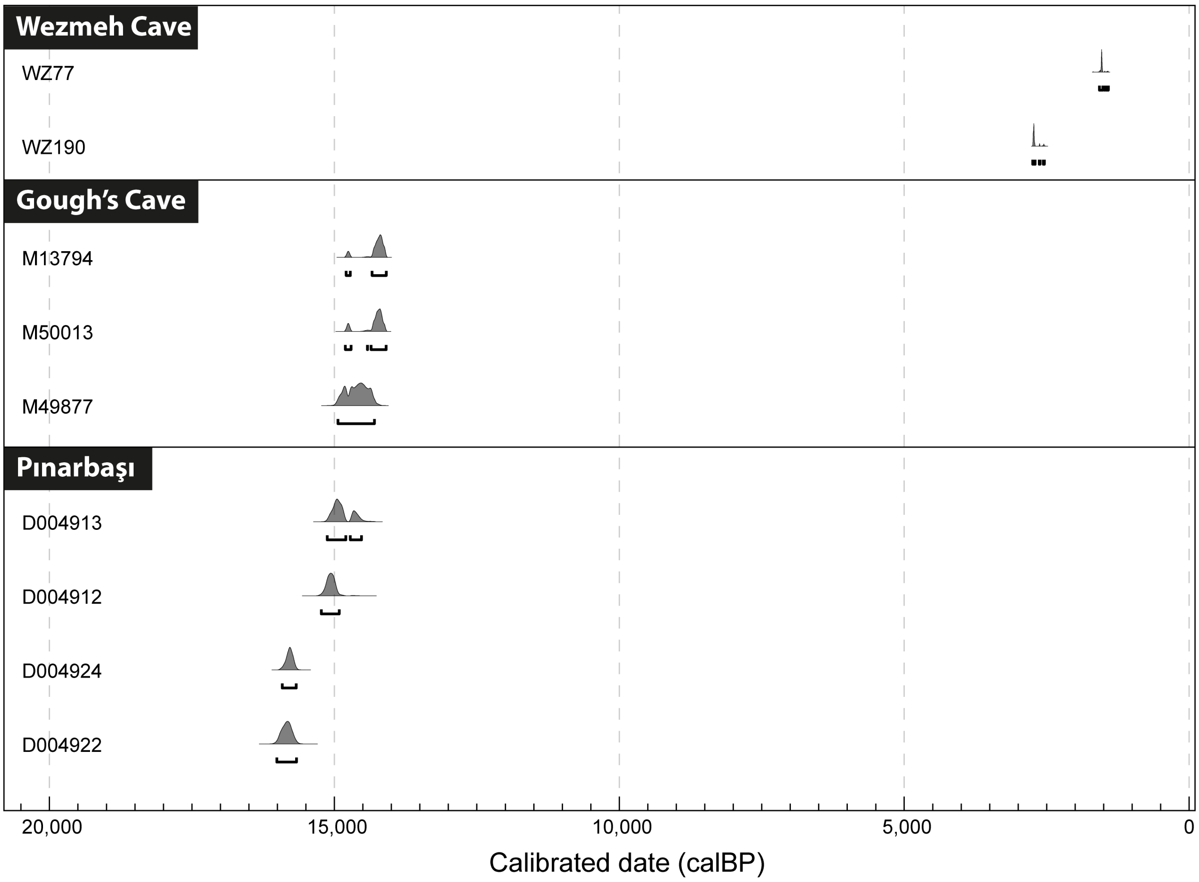

Supplementary Figure 3. Calibrated radiocarbon dates (years calBP) for canids from Pınarbaşı, Gough's Cave, and Wezmeh Cave (see Supplementary Table 2). With the exception of M49877, all dates were generated in this study. Dates were calibrated using OxCal v. 4.4 and the IntCal20 calibration curve, with mean age and 95% confidence-intervals reported.

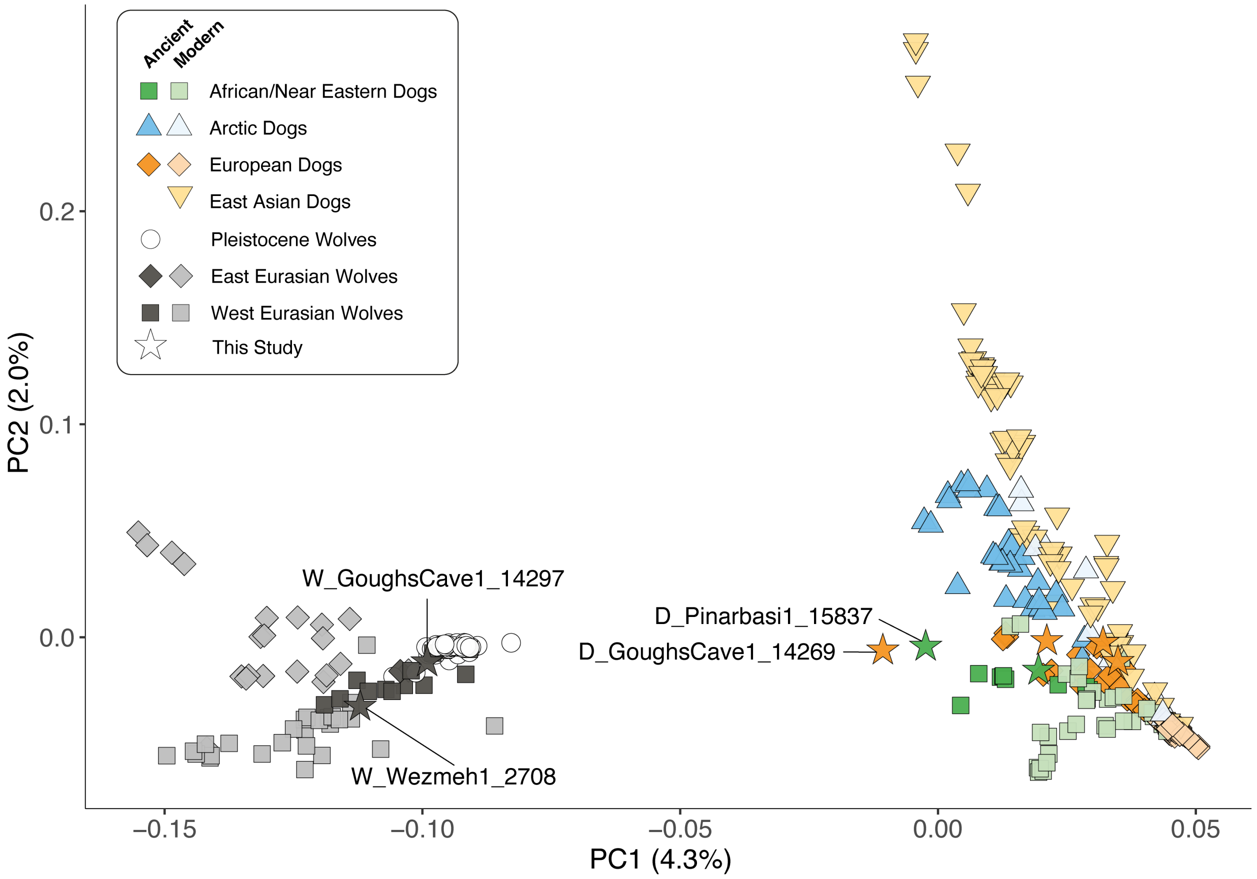

Supplementary Figure 4. Principal component analysis of dogs and wolves (excluding populations in North America) constructed using smartpca. Ancient samples were projected.

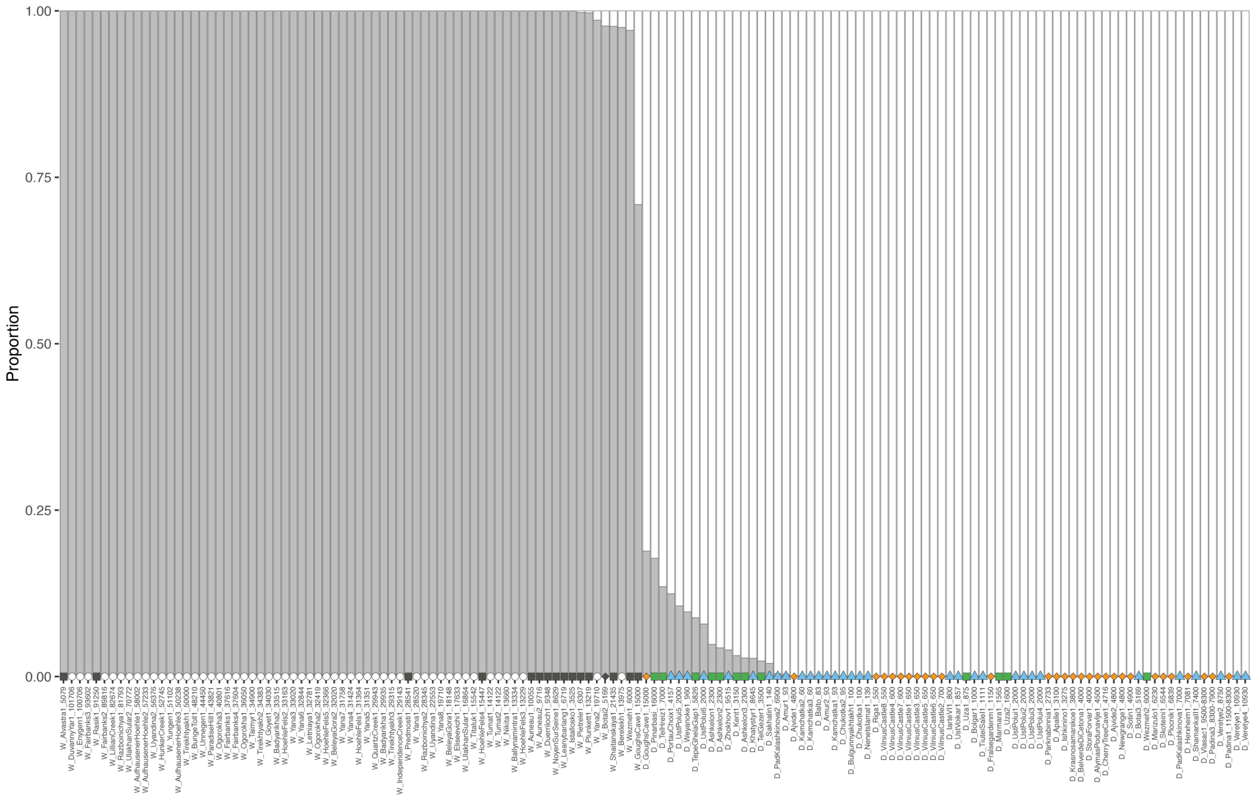

Supplementary Figure 5. Unsupervised ADMIXTURE analysis of canids (y-axis) using two ancestral components (K), which clearly separates wolves (grey) and dogs (white). Coloured shapes correspond to the following major ancestries: Arctic (blue triangle), African and Near Eastern (green square), and European (orange diamond) dogs, and Pleistocene (white circle), West Eurasian (black squares) and East Eurasian (black diamond) wolves.

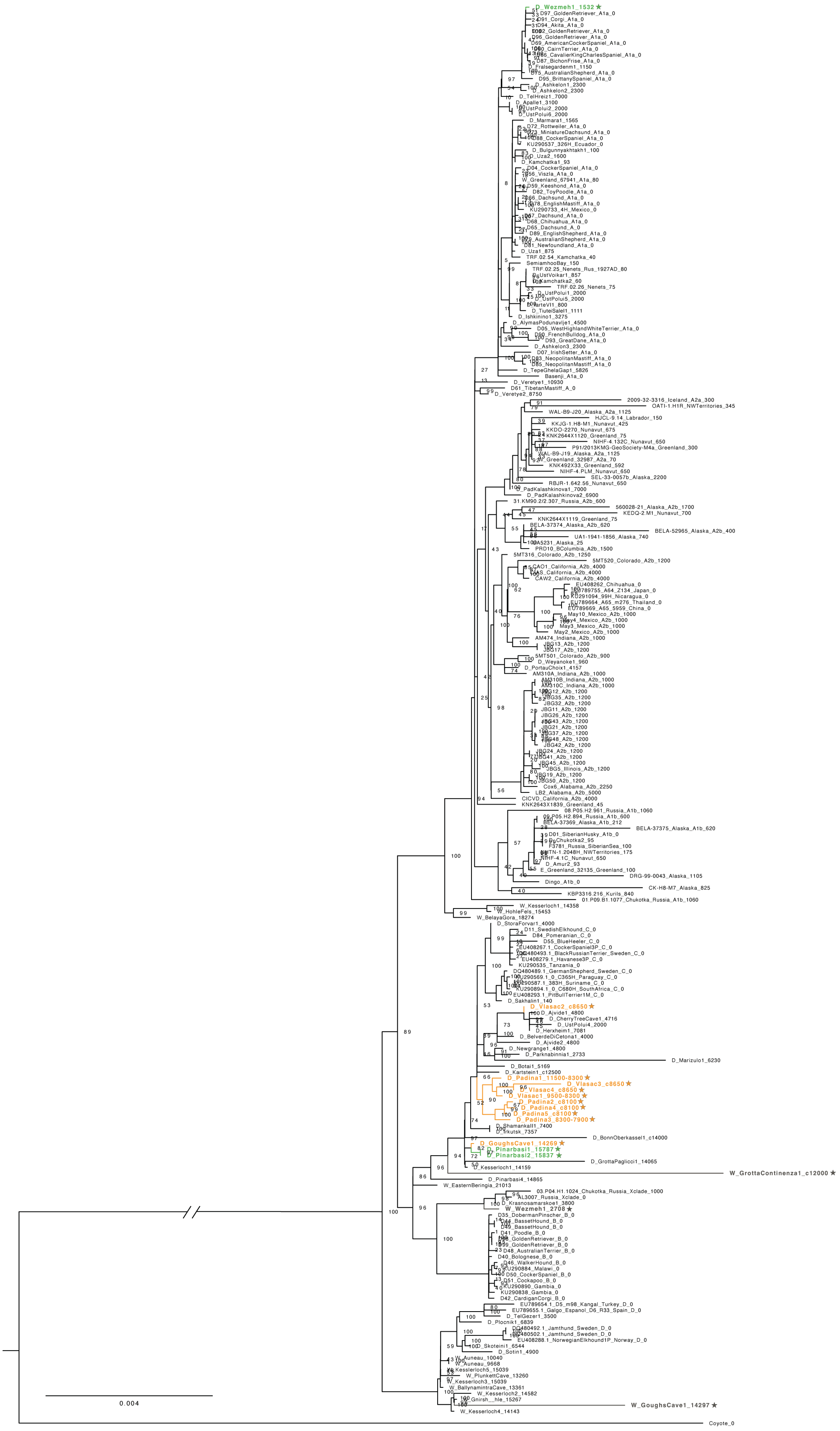

Supplementary Figure 6. Maximum-likelihood tree constructed from 240 canids, with the position of mitochondrial genomes generated in this study (stars) highlighted. Node support values were calculated across 1,000 bootstrap replicates.

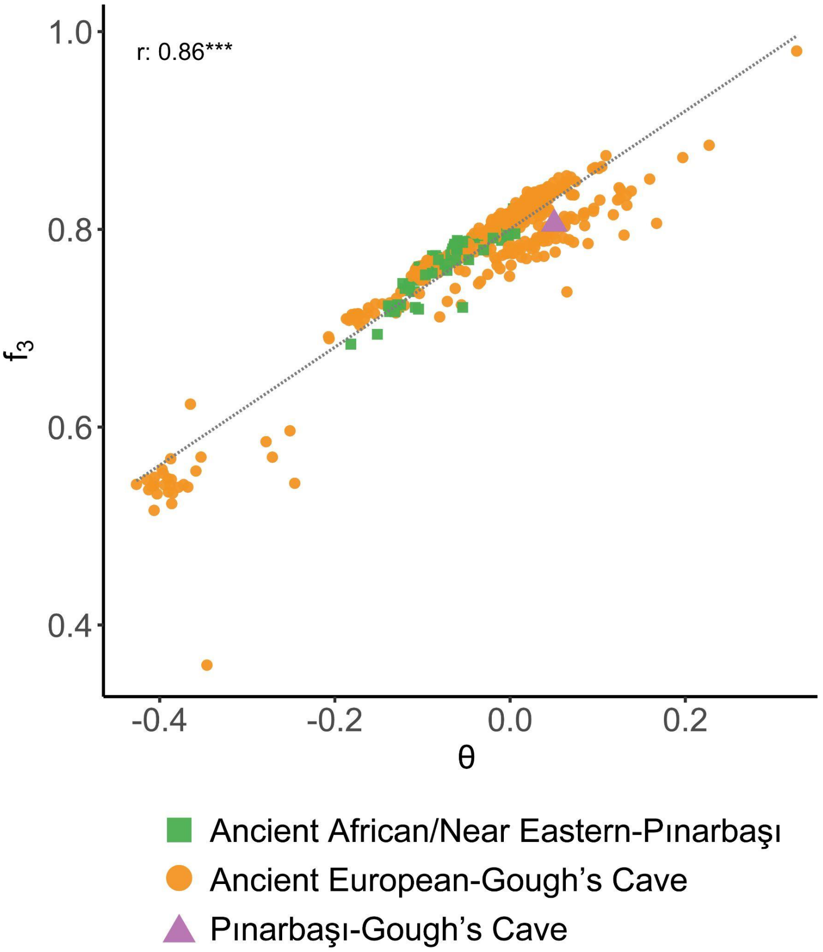

Supplementary Figure 7. Linear regression of normalised kinship coefficients (θ; (x-axis) and corresponding outgroup-f3 values (y-axis) of the form of f3(X, Y; Coyote) of each pair (n=531 comparisons). The grey solid diagonal line represents linear regression. Spearman correlation coefficient: $r = 0.86$, $p < 0.001$.

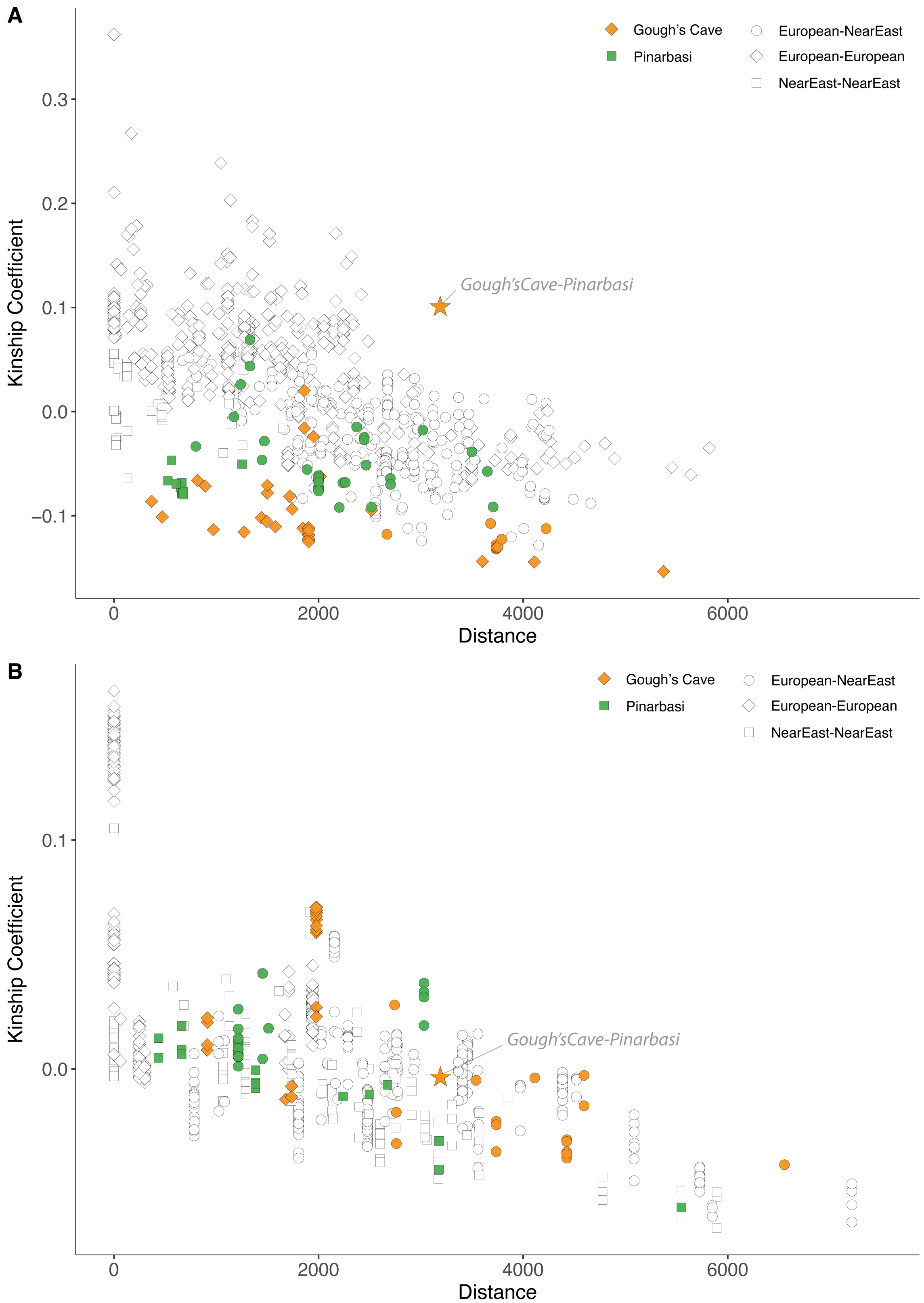

Supplementary Figure 8. Normalised kinship coefficients (θ) of Pınarbaşı and Gough's Cave **(A)** dogs and **(B)** humans calculated in READv2 through comparison against other European (orange diamond) and Near Eastern (green circle) populations. Non-coloured circles reflect population-pairs not including either Pınarbaşı and Gough's Cave. Distance represents Haversine (great-circles) distance in kilometres.

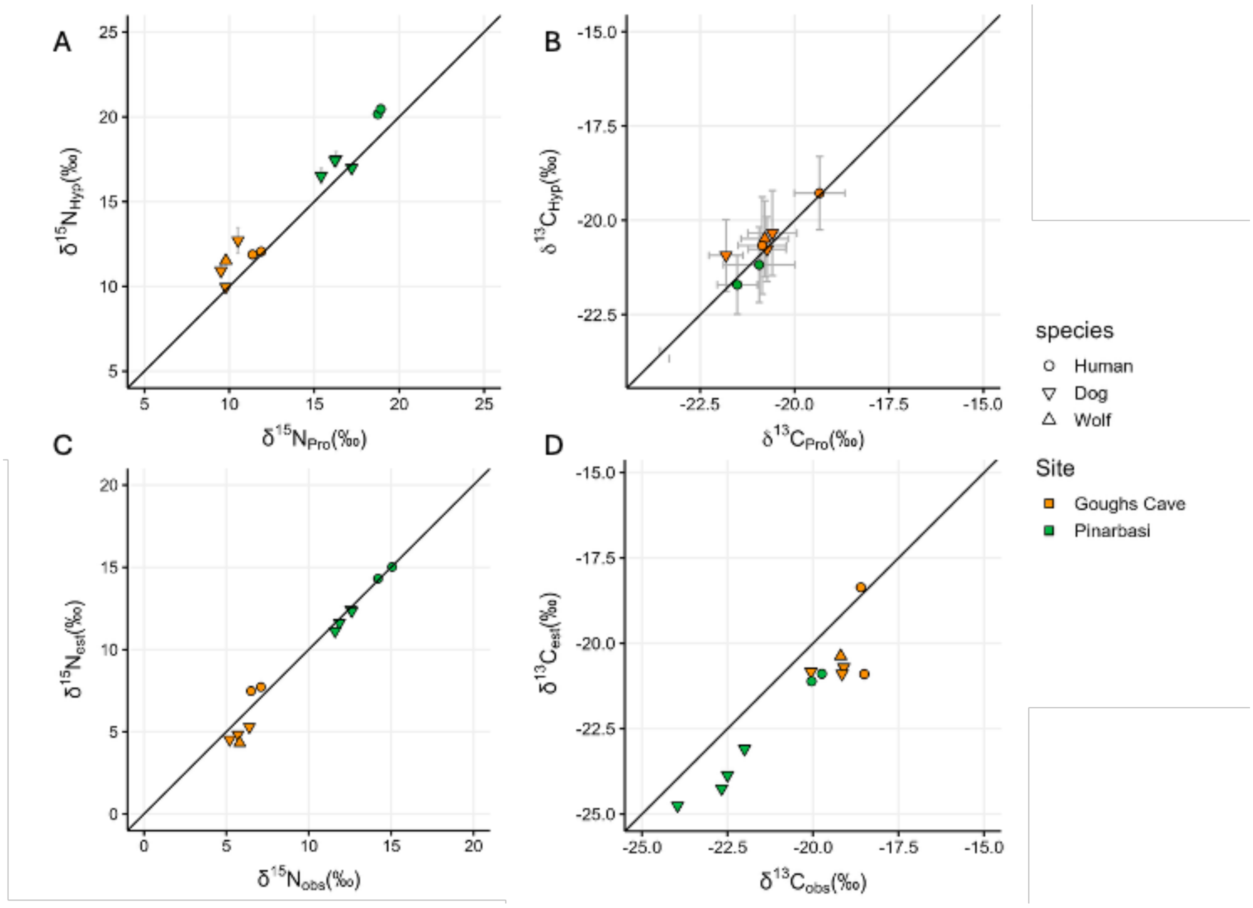

Supplementary Figure 9. Authentication of measured δ^{15}N and δ^{13}C amino acid values. Measured Hydroxyproline (y-axis) vs Proline (x-axis) for δ^{15}N and **(B)** δ^{13}C, where 1:1 ratio is expected. Observed (x-axis) vs Expected (y-axis) bulk isotope values for **(C)** δ^{15}N and **(D)** δ^{13}C. Expected values calculated using mass balance equations that consider the known contribution of each amino acid to bone collagen, observed values measured bulk isotope values.

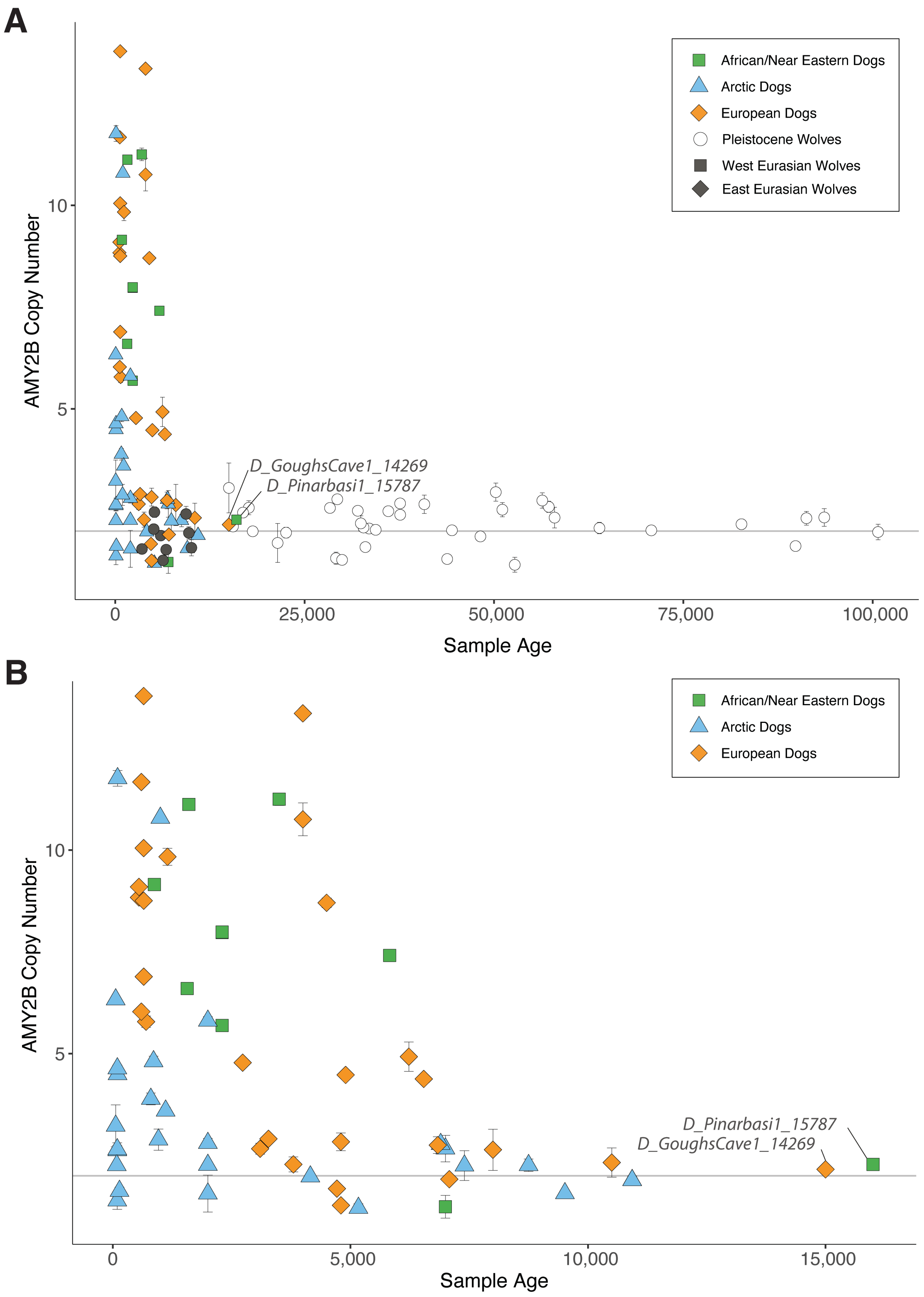

Supplementary Figure 10. Estimates of AMY2B gene copy number in **(A)** ancient dogs and wolves, and **(B)** ancient dogs only, as a function of sample age. Both Pınarbaşı and Gough's Cave possess two copies of AMY2B (similar to ancient wolves), with the earliest evidence for elevated copy number detected in Neolithic Europe. Error bars represent 95% CI.

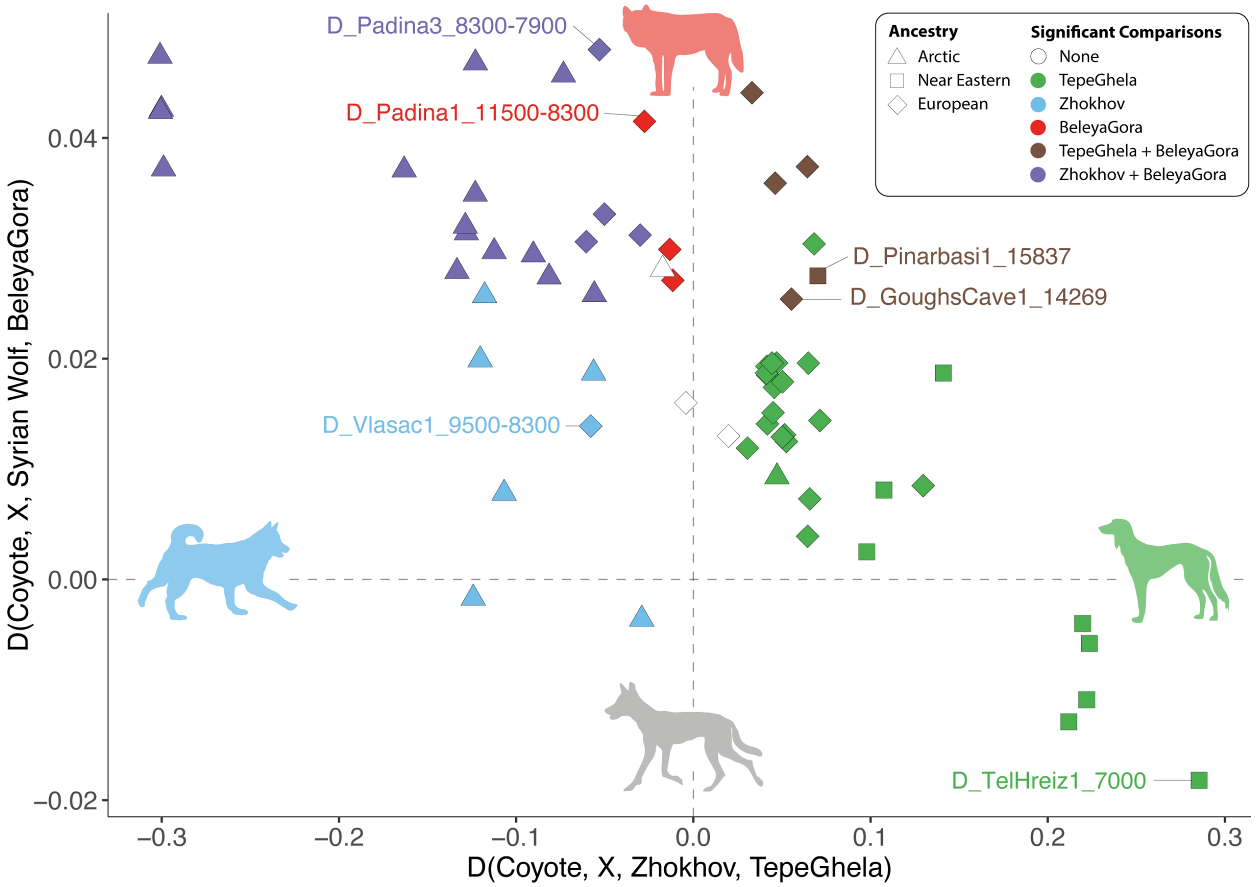

Supplementary Figure 11. D-statistics of the form: D(Coyote, X; D_Zhokhov1_9515, D_TepeGhelaGap1_5826) and: D(Coyote, X; W_BeleyaGora1_18148, Wolf56). Fill colour corresponds to test significance (|Z| < 3; see figure legend). Pınarbaşı and Gough's Cave show significant allele sharing with both TepeGhelaGap and BeleyaGora, which indicates West Eurasian dog ancestry, but not the secondary gene flow from Near Eastern wolves (exemplified here by Tel Hreiz).

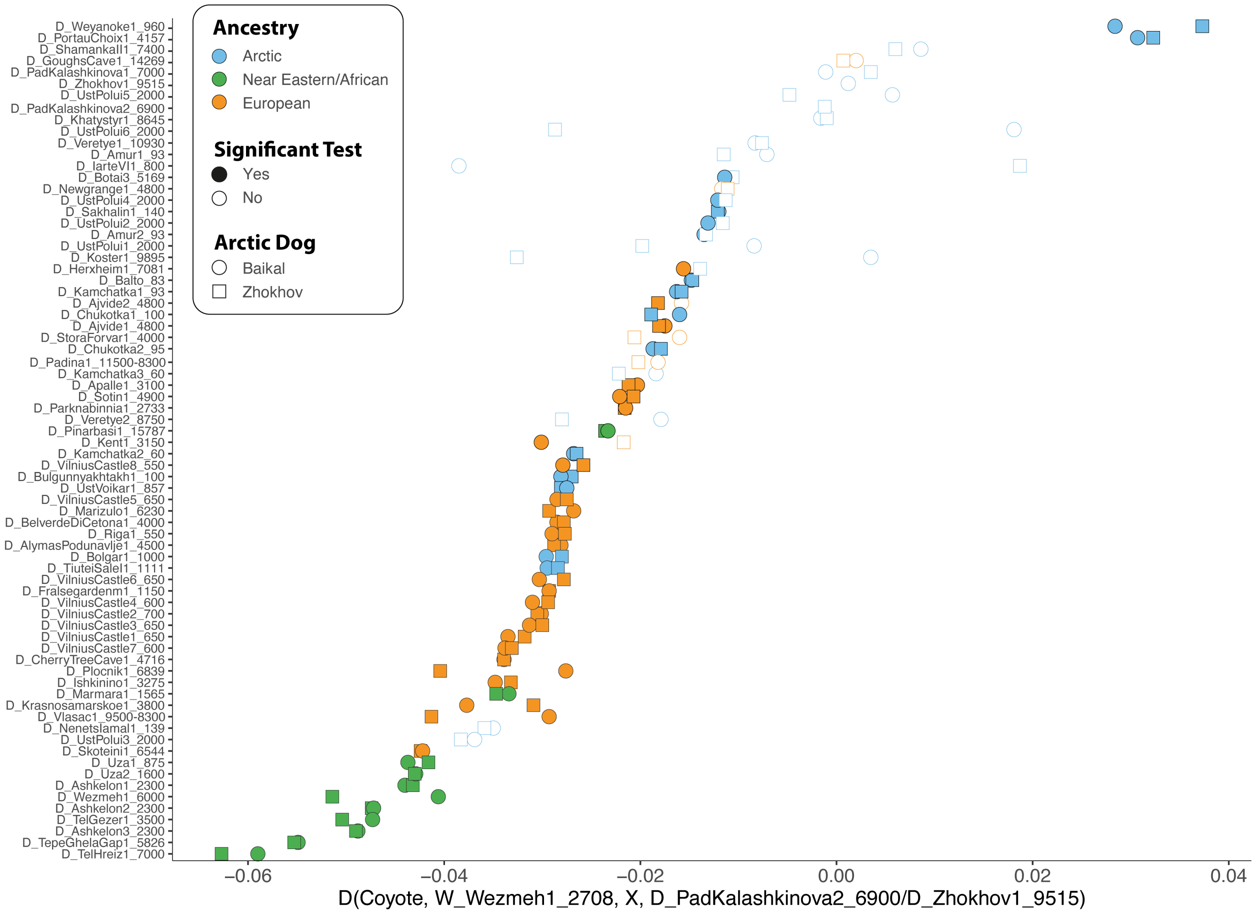

Supplementary Figure 12. D-statistic comparisons of the form: D(Coyote, W_Wezmeh_2708, X, D_PadKalashkinova2_6900/D_Zhokhov1_9515); where X represents all ancient dogs (y-axis). Significantly negative (|Z|<-3) or positive (|Z|>3) values indicate an excess of allele sharing between either the dog on the Y-axis or Arctic dogs, respectively. The majority of West Eurasian dogs show significant allele sharing with the ancient Iranian wolf, which is not seen in East Eurasian dogs (except for historical dogs which possess recent West Eurasian ancestry). This pattern mirrors the trends identified in [99]. With the exception of Pınarbaşı, Near Eastern dogs show an excess of allele sharing relative to all other West Eurasian dogs.

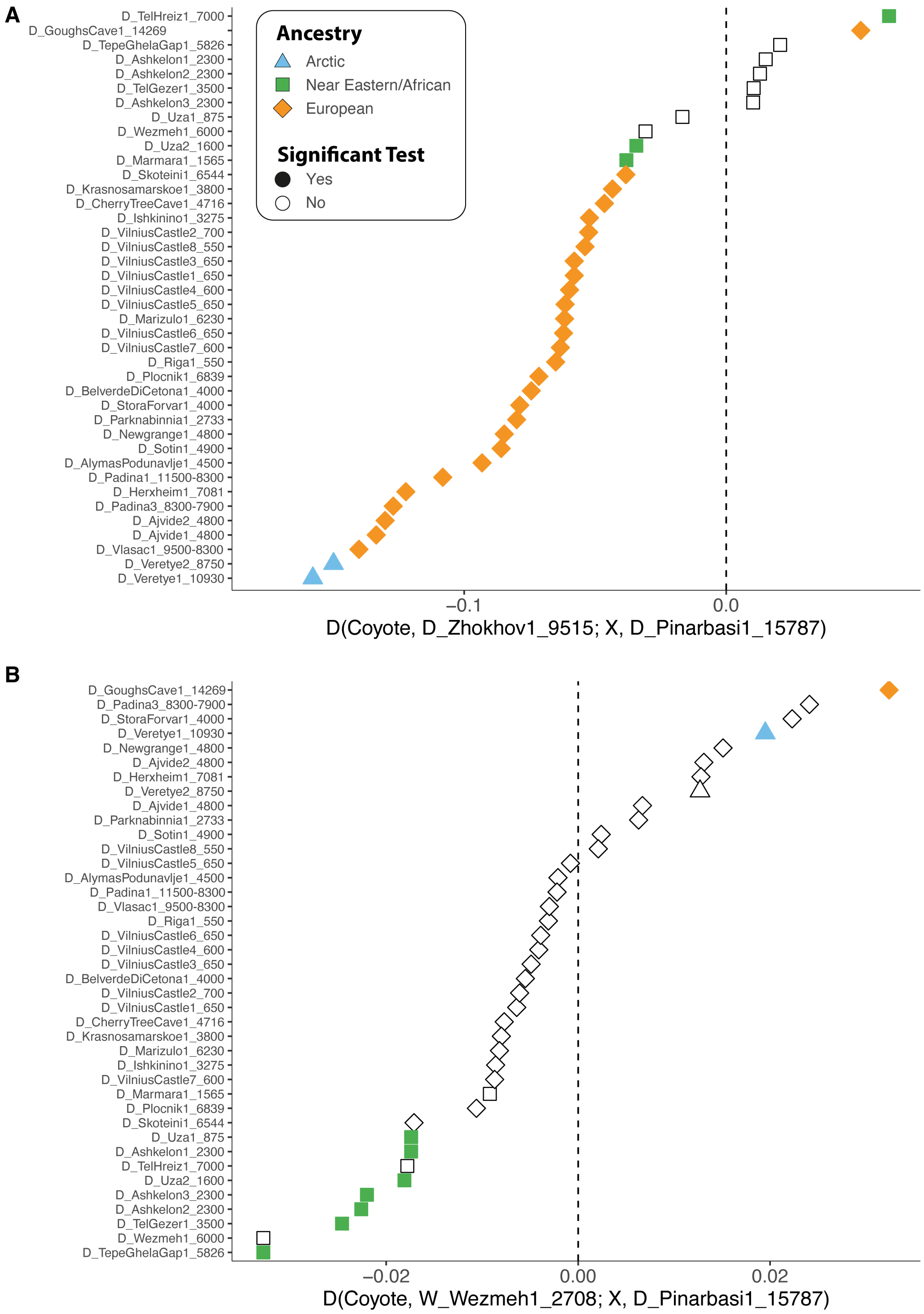

Supplementary Figure 13. D-statistic comparisons of the form: D(Coyote, D_Zhokhov1_9515 **(A)** or W_Wezmeh1_2708 **(B)**, X, D_Pinarbasi1_15787). Significant values (|Z| < 3) are indicated by filled shapes (where colour corresponds to the ancestry of the individual under comparison), with X representing Palaeolithic Holocene dogs (y-axis). In **(A)**, with the exception of Gough's Cave (which is highly positive, likely due to the presence of basal ancestry), European dogs share excess derived alleles with Arctic dogs relative to Pınarbaşı. In contrast, Near Eastern dogs before the Byzantine period lack Arctic ancestry **(A)**, but share an excess of derived alleles with Near Eastern wolves relative to Pınarbaşı **(B)**.

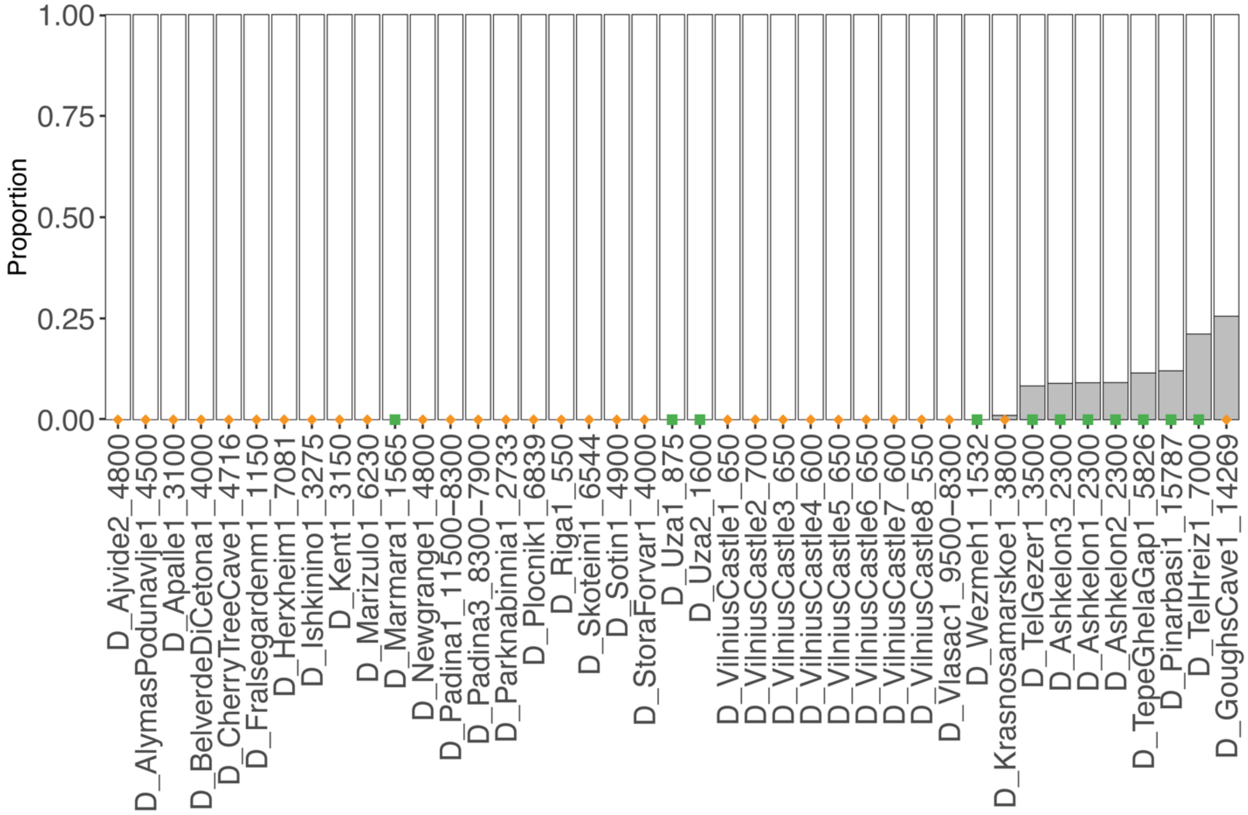

Supplementary Figure 14. Supervised ADMIXTURE analysis using two ancestral components (K) for all ancient West Eurasian dogs. Individuals (on the x-axis) were modelled as a mixture of East Eurasian dog (white: D_Khatystyr1_8645, D_PortauChoix1_4157, D_ShamankaII1_7400, D_Zhokhov1_9515, D_PadKalashkinova2_6900) and Near Eastern wolf (grey: Wolf07, Wolf20, Wolf55, Wolf56, Wolf70, Wolf71, Wolf72, W_Wezmeh1_2708) ancestries. Coloured shapes correspond to the following major ancestries: African and Near Eastern (green square), and European (orange diamond) dogs.

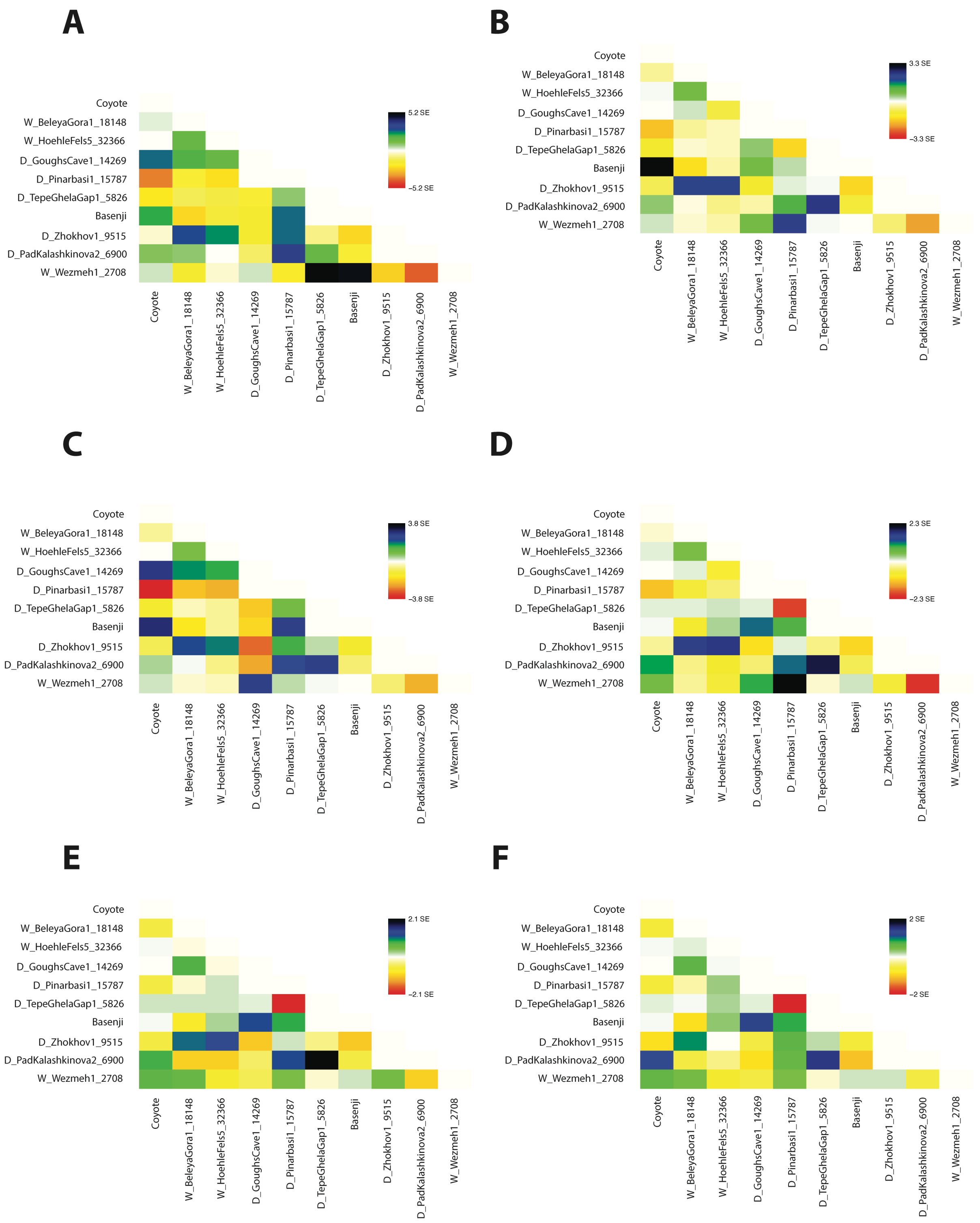

Supplementary Figure 15. Residuals estimated in TreeMix v.1.13 [100] after adding: **(A)** 0, **(B)** 1, **(C)** 2, **(D)** 3, **(E)** 4, and **(F)** 5 migration edges.

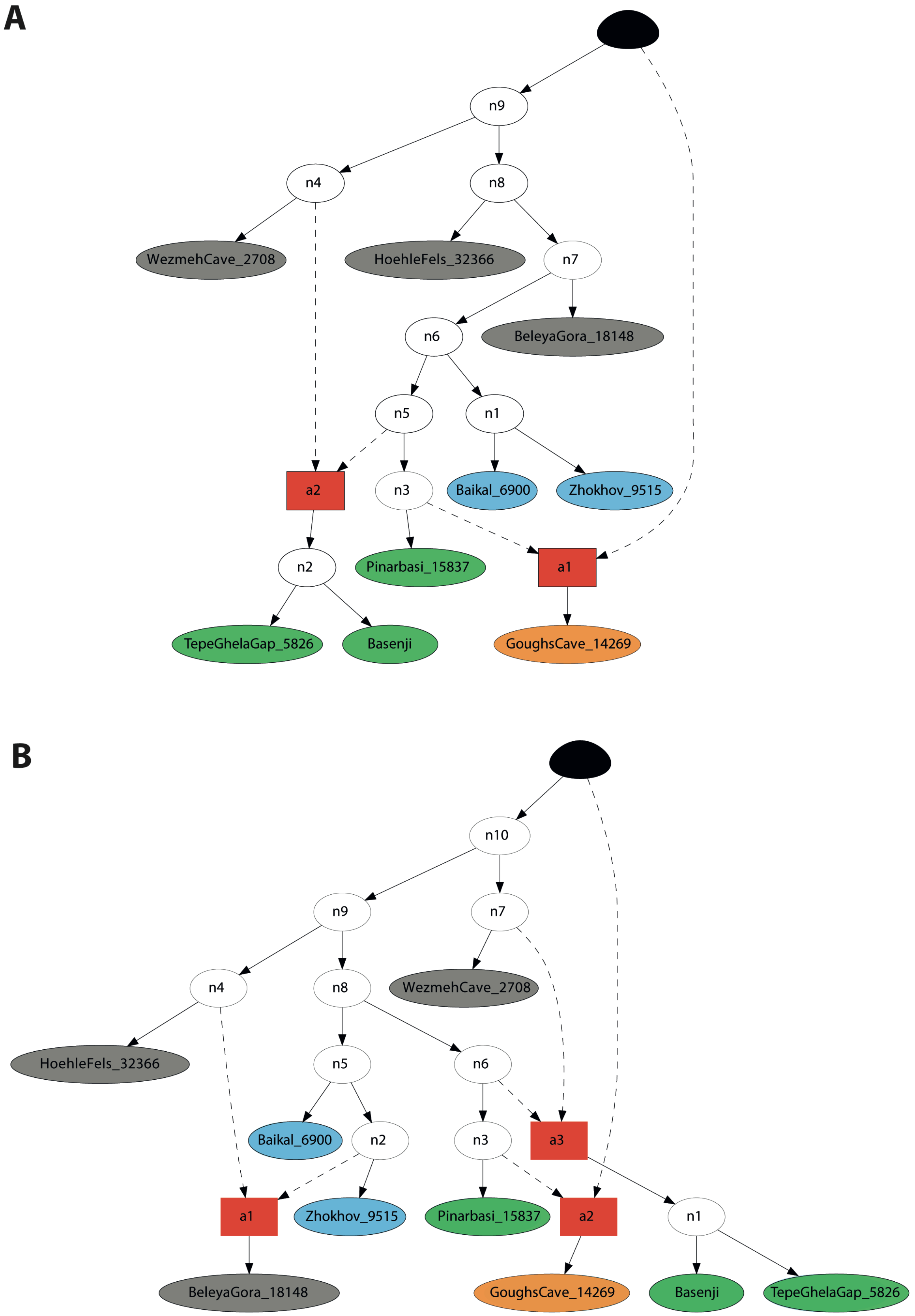

Supplementary Figure 16. Admixture graphs estimated in AdmixtureBayes showing the most well supported admixture graph forcing **(A)** 2 and **(B)** 3 admixture events. Posterior probabilities for trees are as follows: (A) 0.88, (B) 0.14.

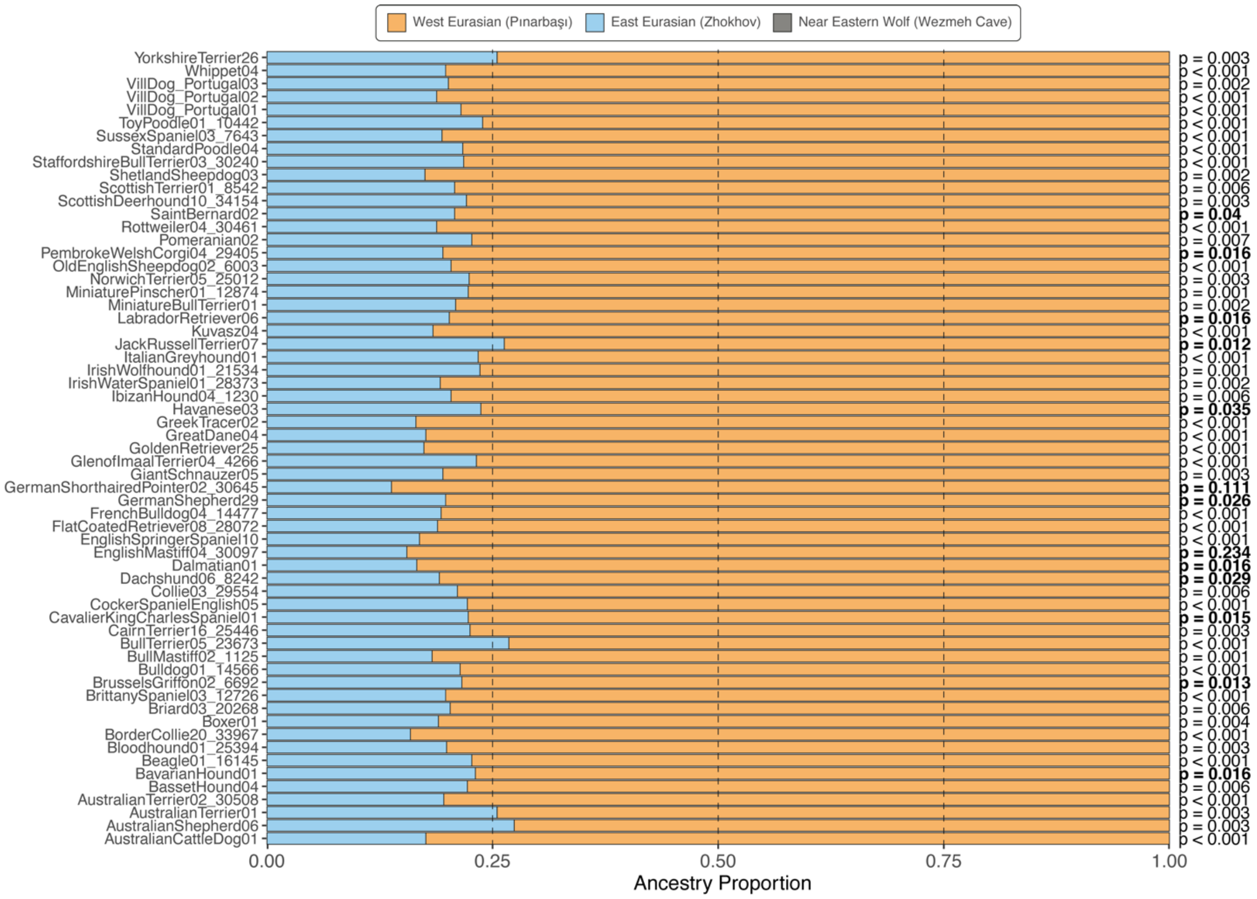

Supplementary Figure 17. Proportion of Palaeolithic West Eurasian (D_Pinarbasi1_15787; orange), East Eurasian (D_Zhokhov1_9515; blue) and Near Eastern wolf (W_Wezmeh1_2708; grey) ancestries in modern European breeds (lefthand y-axis), estimated using qpAdm. Models were deemed significant if they exceed a P-value threshold of 0.01 (bold; righthand y-axis)).

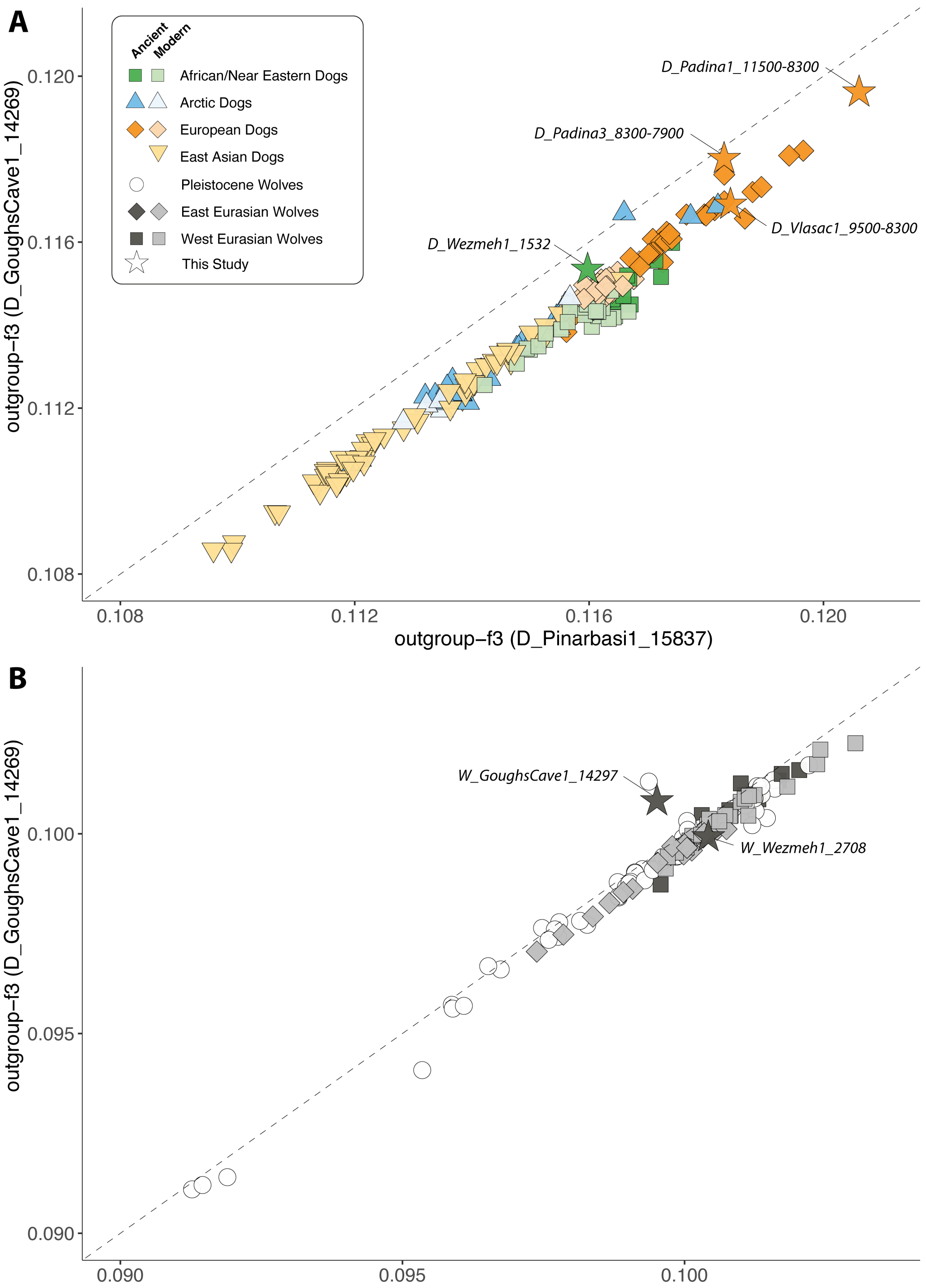

Supplementary Figure 18. (A) Genetic distances between Palaeolithic dogs and all ancient and modern **(A)** dogs and **(B)** wolves, calculated using the following outgroup-f3 test setup: f3(Coyote, X, Pınarbaşı/Gough's Cave). With the exception of W_GoughsCave1_14297 and W_Plunkett1_13219 (a Late Pleistocene wolf from Ireland), most wolves were slightly closer to the Pınarbaşı dog. A similar pattern was identified in dogs, although Mesolithic Serbian dogs (e.g. Padina) were slightly shifted towards Gough's Cave. Altogether, this suggests the Gough's Cave dog may possess basal ancestry, closest to Pleistocene wolf populations in the UK and Ireland.

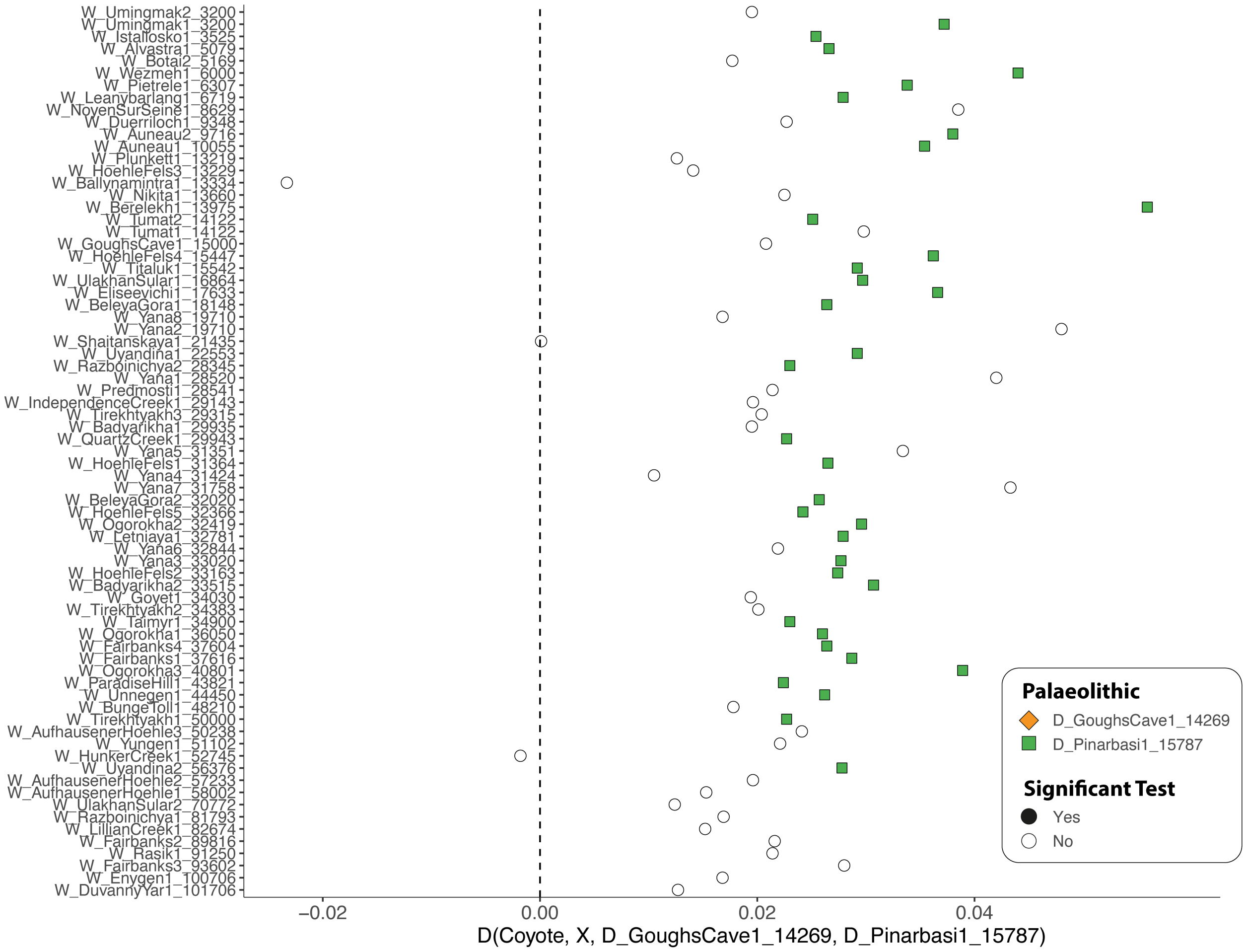

Supplementary Figure 19. D-statistic comparisons of the form: D(Coyote, X, Gough's Cave, Pınarbaşı); where X represents all ancient wolves (ordered by sample age on the y-axis). Significantly negative (|Z|<-3) or positive (|Z|>3) values, which are shown as filled shapes, indicate an excess of allele sharing between either the Gough's Cave or Pınarbaşı dog and the wolf under comparison, respectively. Most wolves share an excess of derived alleles with the Pınarbaşı dog, likely due to the presence of basal wolf ancestry in the Gough's Cave dog.

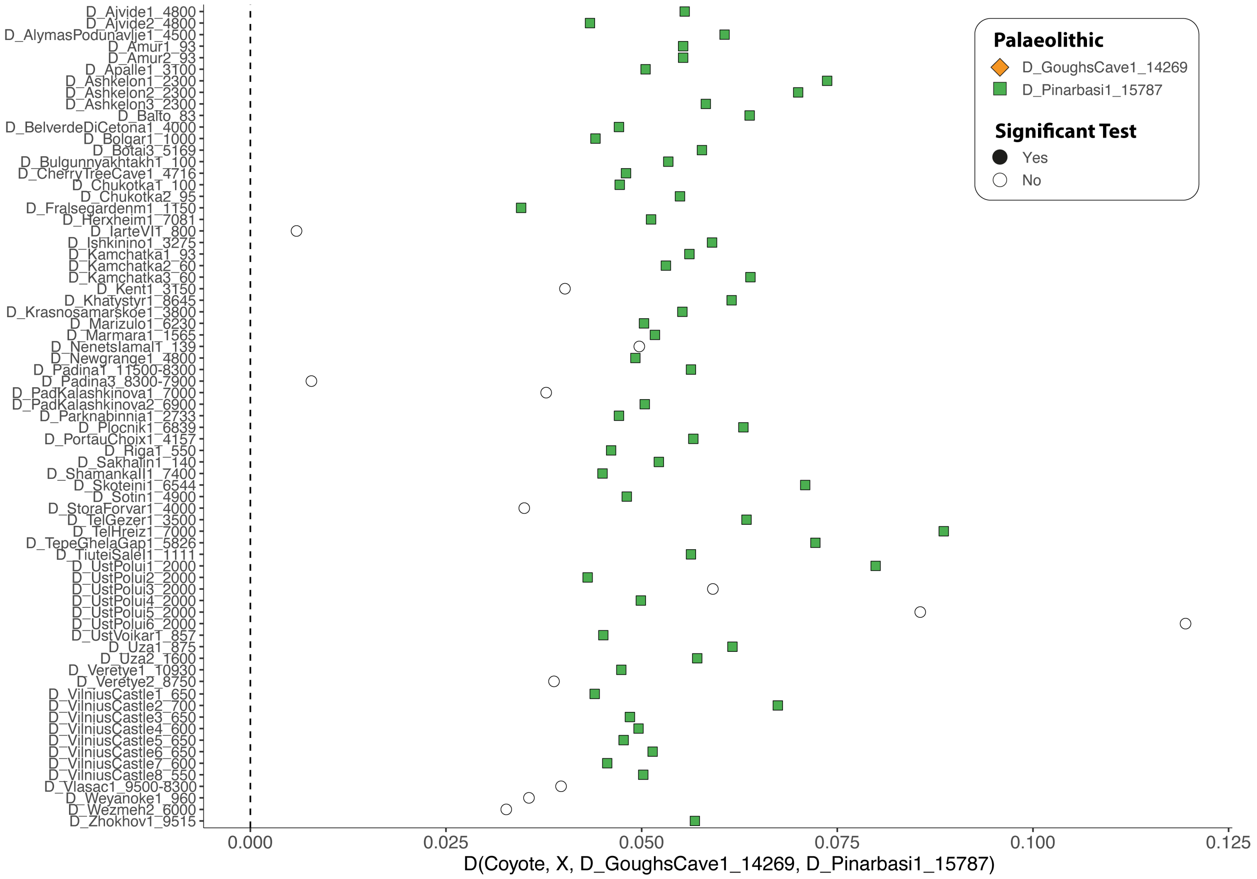

Supplementary Figure 20. D-statistic comparisons of the form: D(Coyote, X, Gough's Cave, Pınarbaşı); where X represents all ancient dogs (y-axis). Significantly negative (|Z|<-3) or positive (|Z|>3) values, which are shown as filled shapes, indicate an excess of allele sharing between either the Gough's Cave or Pınarbaşı dog and the wolf under comparison, respectively. All dogs share an excess of derived alleles with the Pınarbaşı dog, likely due to the presence of basal wolf ancestry in the Gough's Cave dog.

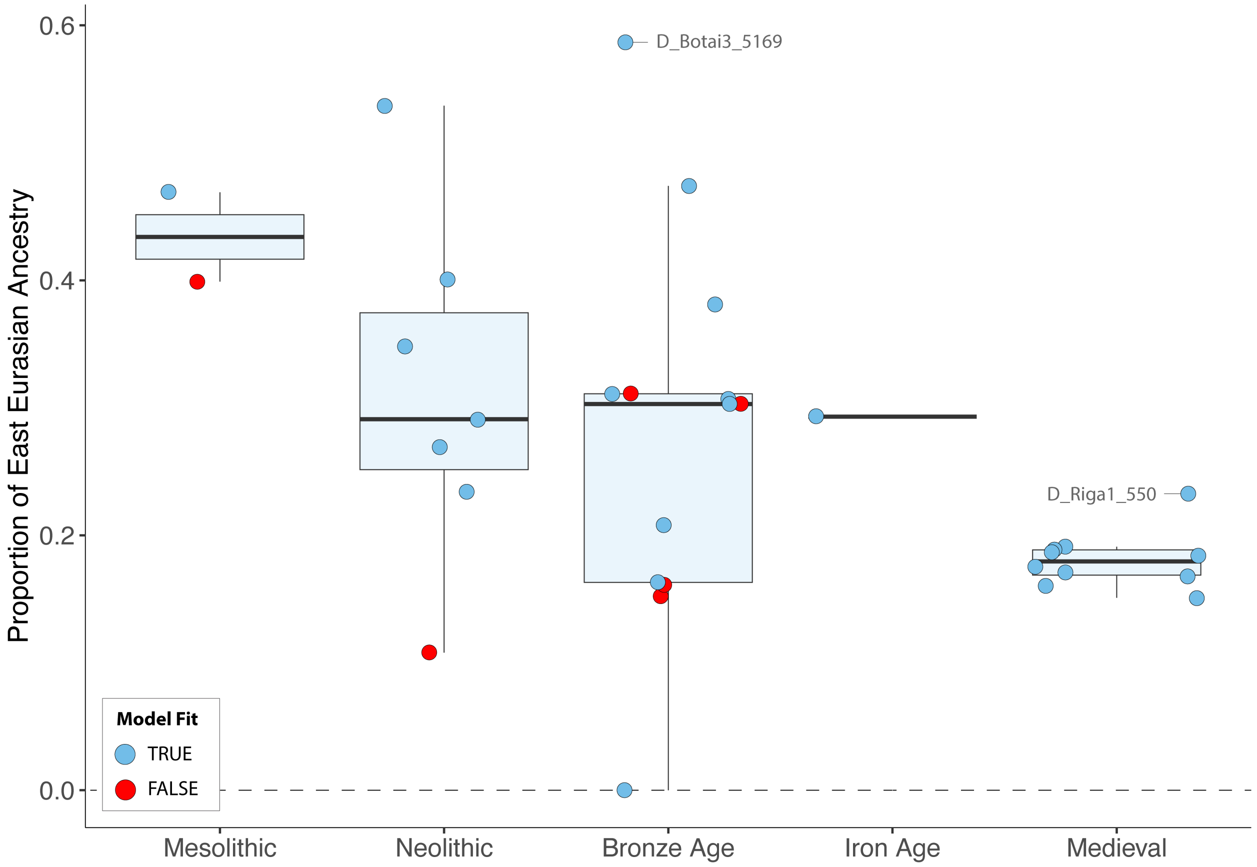

Supplementary Figure 21. Boxplot of the proportion of East Eurasian (D_Zhokhov1_9515) ancestry in Mesolithic to Medieval European dogs, estimated using *qpAdm*. Individuals where dual-source East and West Eurasian dog ancestry (D_Zhokhov1_9515 and D_Pinarbasi1_15787) was not the best fitting model (i.e. P-value does not exceed a threshold of 0.01) are shown in red. Periods are defined as: Mesolithic (13,000–9,000 years ago), Neolithic (9,000–5,200 years ago), Bronze Age (5,200–2,800 years ago), Iron Age (2,800–1,500 years ago) and Medieval (1,500–500 years ago). Statistical outliers are labelled.

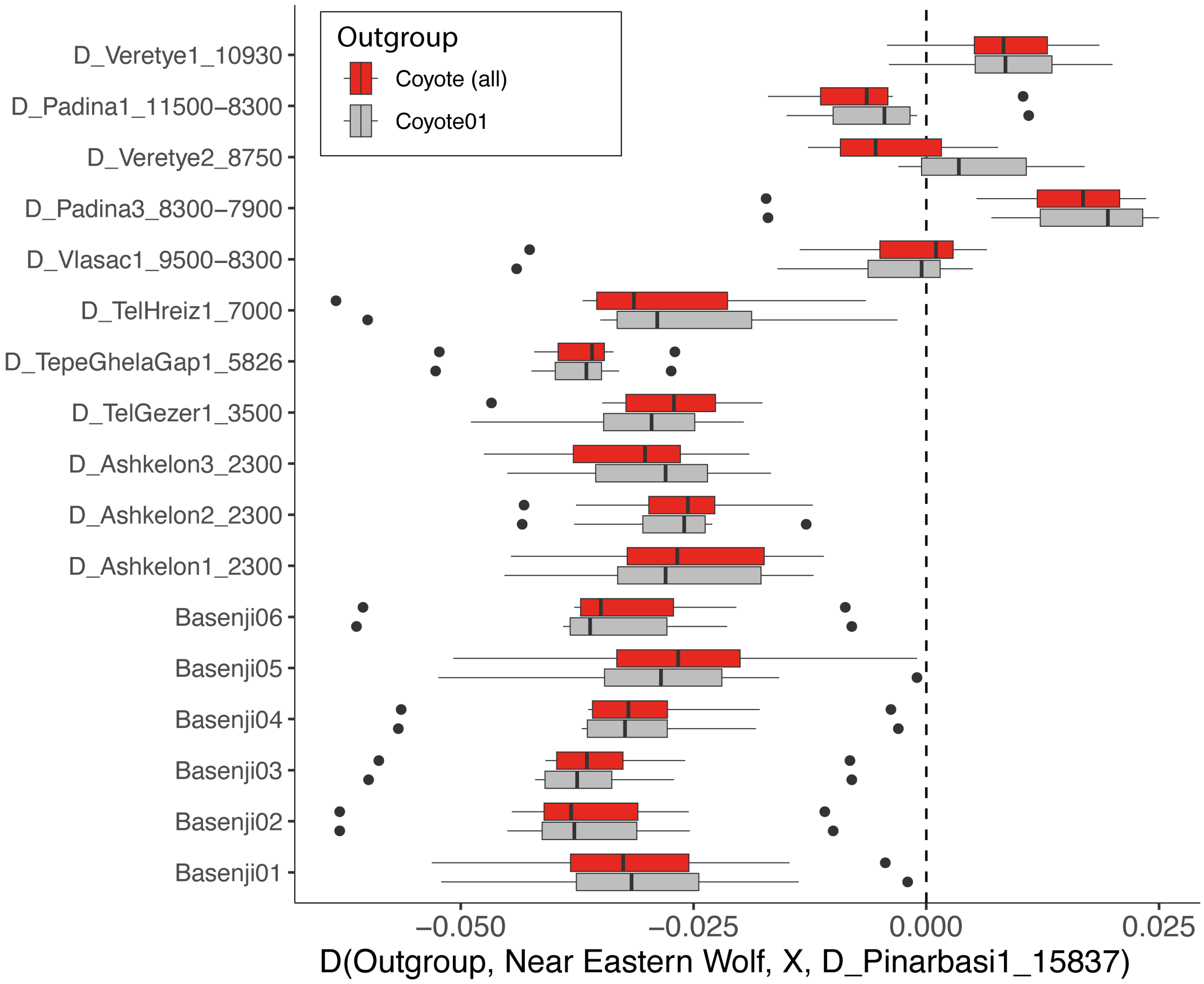

Supplementary Figure 22. Boxplot of D-statistic comparisons of the form: D(Outgroup, Near Eastern Wolf, X, D_Pinarbasi1_15787), where "Outgroup" is either Coyote01 (grey), or all four coyotes (red). A representative subset of Holocene dogs (X) included on the y-axis. There was no significant difference between the two tests for each individual, indicating a negligible impact of variable dog ancestry in coyotes on allele polarisation.

6. References

1. Currant, A. P., Jacobi, R. M. & Stringer, C. B. Excavations at Gough's cave, Somerset 1986–7. *Antiquity* **63**, 131–136 (1989).
2. Davies, H. N. The discovery of human remains under the stalagmite-floor of gough's cavern, Cheddar. *Q. J. Geol. Soc.* **60**, 335–348 (1904).
3. Stringer, C. B. The hominid remains from Gough's Cave. *Proceedings of the University of Bristol Spelaeological Society* **17**, 145–152 (1985).
4. Parkin, R. A. At Gough's Cave, Cheddar, Somerset. *Proceedings of the University of Bristol Spelæological Society* **17**, 311–330 (1986).
5. Jacobi, R. M. & Higham, T. F. G. The early Lateglacial re-colonization of Britain: new radiocarbon evidence from Gough's Cave, southwest England. *Quat. Sci. Rev.* **28**, 1895–1913 (2009).
6. Jacobi, R. The late upper Palaeolithic lithic collection from gough's cave, cheddar, somerset and human use of the cave. *Proc. Prehist. Soc.* **70**, 1–92 (2004).
7. Cook, J. Preliminary report on marked human bones from the 1986-1987 excavations at Gough's Cave, Somerset, England. *Anthropologie* 181–187 (1991).
8. Reimer, P. J. *et al.* The IntCal20 Northern Hemisphere Radiocarbon Age Calibration Curve (0–55 cal kBP). *Radiocarbon* **62**, 725–757 (2020).
9. Charlton, S. *et al.* Dual ancestries and ecologies of the Late Glacial Palaeolithic in Britain. *Nat. Ecol. Evol.* **6**, 1658–1668 (2022).
10. Bello, S. M., Saladié, P., Cáceres, I., Rodríguez-Hidalgo, A. & Parfitt, S. A. Upper Palaeolithic ritualistic cannibalism at Gough's Cave (Somerset, UK): The human remains from head to toe. *J. Hum. Evol.* **82**, 170–189 (2015).
11. Bello, S. M., Parfitt, S. A. & Stringer, C. B. Earliest directly-dated human skull-cups. *PLoS One* **6**, e17026 (2011).
12. Marsh, W. A. & Bello, S. Cannibalism and burial in the late Upper Palaeolithic: Combining archaeological and genetic evidence. *Quat. Sci. Rev.* **319**, 108309 (2023).
13. Jacobi, R. & Higham, T. The later upper Palaeolithic recolonisation of Britain: New results from AMS radiocarbon dating. in *Developments in Quaternary Sciences* 223–247 (Elsevier, 2011).
14. Janssens, L. *et al.* A new look at an old dog: Bonn-Oberkassel reconsidered. *J. Archaeol. Sci.* **92**, 126–138 (2018).
15. Baird, D. The Epipalaeolithic of the Anatolian plateau in its SW Asian context: insights from Pınarbaşı. in *Prehistoric Anatolia and Cyprus. Studies In Mediterranean Archaeology.* (ed. Baird D, C. J.) 13–30 (Astrom Editions, Nicosia, 2025).
16. Baird, D. *et al.* Juniper smoke, skulls and wolves' tails. The Epipalaeolithic of the Anatolian plateau in its South-west Asian context; insights from Pınarbaşı. *Levantina* **45**, 175–209 (2013).
17. Feldman, M. *et al.* Late Pleistocene human genome suggests a local origin for the first farmers of central Anatolia. *Nat. Commun.* **10**, 1218 (2019).
18. Altınbilek-Algül, C., Kayci, O. & Balcı, S. A New Epipalaeolithic Site in the Central Taurus Mountains: Eşek Deresi Cave (Mersin/Turkey). *ArchéOrient-Le Blog* **18**, 1–9 (2022).
19. Moore, A., Tangye, M., Hillman, G. C. & Legge, A. J. *Village on the Euphrates: From Foraging to Farming at Abu Hureyra*. (Oxford University Press, Oxford, 2000).
20. Carter, T. *et al.* Marginal perspectives: Sourcing epi-Palaeolithic to Chalcolithic obsidian from the Öküzini cave (SW turkey). *Paleobiology* **37**, 123–149 (2011).

21. Pettitt, P. *The Palaeolithic Origins of Human Burial*. (Routledge, 2010).
22. Sparacello, V. S. *et al.* New insights on Final Epigravettian funerary behavior at Arene Candide Cave (Western Liguria, Italy). *J. Anthropol. Sci.* **96**, 161–184 (2018).
23. Russell, N. Changing use of birds across the agricultural transition at Pınarbaşı, Turkey. *Quat. Int.* **626-627**, 43–51 (2022).
24. Feider, M. Scourge or Sustenance: Using microfauna to explore the palaeoenvironment and palaeoeconomics of Epipalaeolithic and early Neolithic communities in Anatolia. (Bournemouth University, 2022).
25. Baird, D. *et al.* Agricultural origins on the Anatolian plateau. *Proc. Natl. Acad. Sci. U. S. A.* **115**, E3077–E3086 (2018).
26. Davis, S. When and why did prehistoric people domesticate animals? Some evidence from Israel and Cyprus. in *The Natufian Culture in the Levant. International monographs in Prehistory* (ed. Bar-Yosef O, V. F.) 381–390 (Ann Arbor, 1991).
27. Boschian, G., Serradimigni, M., Colombo, M., Ghislandi, S. & Grifoni Cremonesi, R. Change fast or change slow? Late Glacial and Early Holocene cultures in a changing environment at Grotta Continenza, Central Italy. *Quat. Int.* **450**, 186–208 (2017).
28. Giraudi, C. Lake levels and climate for the last 30,000 years in the fucino area (Abruzzo-Central Italy) — A review. *Palaeogeogr. Palaeoclimatol. Palaeoecol.* **70**, 249–260 (1989).
29. Serradimigni, M. *et al.* Spatial analysis of the portable art objects in the epigravettian deposit of the Grotta continenza (abruzzo, Italy) and their relationship to the burials and combustion features. *Palethnologie* (2013) doi:10.4000/palethnologie.5034.
30. Nava, A. *et al.* Multipronged dental analyses reveal dietary differences in last foragers and first farmers at Grotta Continenza, central Italy (15,500-7000 BP). *Sci. Rep.* **11**, 4261 (2021).
31. Mashkour, M. *et al.* Carnivores and their prey in the Wezmeh Cave (Kermanshah, Iran): a Late Pleistocene refuge in the Zagros. *Int. J. Osteoarchaeol.* **19**, 678–694 (2009).
32. Djamali, M. *et al.* Pollen analysis of coprolites from a late Pleistocene–Holocene cave deposit (Wezmeh Cave, west Iran): insights into the late Pleistocene and late Holocene vegetation and flora of the central Zagros Mountains. *J. Archaeol. Sci.* **38**, 3394–3401 (2011).
33. Abdi, K., Biglari, F. & Heydari, S. Islamabad project 2001. *Test excavations at Wezmeh cave*. *Archäol Mitt Iran Turan* **34**, 171–194 (2002).
34. Biglari, F. *et al.* Excavation at the Wezmeh Cave and survey of the surrounding area in the Qaziwand Mountains, The Islamabad Plain, Kermanshah. in *A Collection of Archaeological Finds From Excavations in Iran, 2019-2020* (Research Institute of Cultural Heritage & Tourism, 2021).
35. Monchot, H. Des hyènes tachetées au Pléistocène supérieur dans le Zagros (grotte Wezmeh, Iran). *MOM Éditions* **49**, 65–78 (2008).
36. Monchot, H., Mashkour, M., Biglari, F. & Abdi, K. The Upper Pleistocene brown bear (Carnivora, Ursidae) in the Zagros: Evidence from Wezmeh Cave, Kermanshah, Iran. *Ann. Paleontol.* **106**, 102381 (2020).
37. Trinkaus, E. *et al.* Late Pleistocene human remains from Wezmeh Cave, western Iran. *Am. J. Phys. Anthropol.* **135**, 371–378 (2008).
38. Zanolli, C. *et al.* A Neanderthal from the Central Western Zagros, Iran. Structural

reassessment of the Wezmeh 1 maxillary premolar. *J. Hum. Evol.* **135**, 102643 (2019).
39. Broushaki, F. *et al.* Early Neolithic genomes from the eastern Fertile Crescent. *Science* **353**, 499–503 (2016).
40. Srejović, D. *Lepenski Vir – Nova Praistorijska Kultura U Podunavlju*. (Srpska knji_zevna zadruga, Beograd, 1969).
41. Srejović, D. *Europe's First Monumental Sculpture: New Discoveries at Lepenski Vir*. (Thames and Hudson, London, 1972).
42. Borić, D. Adaptations and Transformations of the Danube Gorges Foragers (c.13,000-5500cal.BC): an overview. in *Beginnings – New Research in the Appearance of the Neolithic Between Northwest Anatolia and the Carpathian Basin* (ed. Krauß, R.) 157–203 (Leidorf, 2011).
43. Radovanović, I. *The Iron Gates Mesolithic*. (International Monographs in Prehistory, Michigan, 1996).
44. Bonsall, C. The Mesolithic of the Iron Gates. in *Mesolithic Europe* (eds. Bailey, G. & Spikins, P.) 238–279 (Cambridge University Press, Cambridge, 2009).
45. Bonsall, C. & Boroneanț, A. The Iron Gates Mesolithic–a brief review of recent developments. *Anthropologie* **122**, 264–280 (2018).
46. Borić, D., French, C. & Dimitrijević, V. Vlasac revisited: formation processes, stratigraphy and dating. *Documenta Praehistorica* **35**, 1–28 (2008).
47. Dimitrijević, V., Živaljević, I. & Stefanović, S. Becoming sedentary? The seasonality of food resource exploitation in the Mesolithic-Neolithic Danube gorges. *Doc. Praehist.* **43**, 103–122 (2016).
48. Borić, D. & Dimitrijević, V. When did the 'Neolithic package reach Lepenski Vir? Radiometric and faunal evidence. *Documenta Praehistorica* **34**, 53–71 (2007).
49. Borić, D. *et al.* Late Mesolithic lifeways and deathways at Vlasac (Serbia). *Journal of Field Archaeology* **39**, 4–31 (2014).
50. Jovanović, J. *et al.* Last hunters–first farmers: new insight into subsistence strategies in the Central Balkans through multi-isotopic analysis. *Archaeol. Anthropol. Sci.* **11**, 3279–3298 (2019).
51. Mathieson, I. *et al.* The genomic history of southeastern Europe. *Nature* **555**, 197–203 (2018).
52. Marchi, N. *et al.* The genomic origins of the world's first farmers. *Cell* **185**, 1842–1859.e18 (2022).
53. Borić, D. New Discoveries at the Mesolithic-Early NeolithicSite of Vlasac: Preliminary Notes. *Mesolithic Miscellany* **18**, 7–14 (2006).
54. Jovanović, B. Chronological Frames of the Iron Gate Group of the Early Neolithic Period. *Archaeologica Iugoslavica* **10**, 23–38 (1969).
55. Jovanović, B. Micro-regions of the Lepenski Vir culture: Padina in the Upper Gorge and Hajdučka Vodenica in the Lower Gorge of the Danube. *Doc. Praehist.* **35**, 289–324 (2008).
56. Živanović, S. A note on the anthropological characteristics of the Padina population. *Z. Morphol. Anthropol.* **66**, 161–175 (1975).
57. Dimitrijević, V. & Vuković, S. Was the dog locally domesticated in the Danube gorges? Morphometric study of dog cranial remains from four Mesolithic-early neolithic archaeological sites by comparison with contemporary wolves: Dog domestication in the Danube gorges Mesolithic. *Int. J. Osteoarchaeol.* **25**, 1–30 (2015).
58. Higham, T. F. G., Jacobi, R. M. & Ramsey, C. B. AMS radiocarbon dating of ancient bone using ultrafiltration. *Radiocarbon* **48**, 179–195 (2006).

59. Brock, F., Higham, T., Ditchfield, P. & Ramsey, C. B. Current Pretreatment Methods for AMS Radiocarbon Dating at the Oxford Radiocarbon Accelerator Unit (Orau). *Radiocarbon* **52**, 103–112 (2010).
60. Santos, G. M. *et al.* AMS 14C sample preparation at the KCCAMS/UCI facility: Status report and performance of small samples. *Radiocarbon* **49**, 255–269 (2007).
61. Beverly, R. K. *et al.* The Keck carbon cycle AMS laboratory, University of California, Irvine: Status report. *Radiocarbon* **52**, 301–309 (2010).
62. Longin, R. New method of collagen extraction for radiocarbon dating. *Nature* **230**, 241–242 (1971).
63. Stuiver, M. & Polach, H. A. Discussion Reporting of 14C Data. *Radiocarbon* **19**, 355–363 (1977).
64. Wood, R. E., Bronk Ramsey, C. & Higham, T. F. G. Refining Background Corrections for Radiocarbon Dating of Bone Collagen at Orau. *Radiocarbon* **52**, 600–611 (2010).
65. Bronk Ramsey, C., Higham, T., Bowles, A. & Hedges, R. Improvements to the pretreatment of bone at oxford. *Radiocarbon* **46**, 155–163 (2004).
66. DeNiro, M. J. Postmortem preservation and alteration of in vivo bone collagen isotope ratios in relation to palaeodietary reconstruction. *Nature* **317**, 806–809 (1985).
67. van Klinken, G. J. Bone collagen quality indicators for palaeodietary and radiocarbon measurements. *J. Archaeol. Sci.* **26**, 687–695 (1999).
68. Styring, A. K. *et al.* Refining human palaeodietary reconstruction using amino acid δ15N values of plants, animals and humans. *J. Archaeol. Sci.* **53**, 504–515 (2015).
69. Docherty, G., Jones, V. & Evershed, R. P. Practical and theoretical considerations in the gas chromatography/combustion/isotope ratio mass spectrometry δ13C analysis of small polyfunctional compounds. *Rapid Commun. Mass Spectrom.* **15**, 730–738 (2001).
70. Dabney, J. *et al.* Complete mitochondrial genome sequence of a Middle Pleistocene cave bear reconstructed from ultrashort DNA fragments. *Proc. Natl. Acad. Sci. U. S. A.* **110**, 15758–15763 (2013).
71. Gamba, C. *et al.* Comparing the performance of three ancient DNA extraction methods for high-throughput sequencing. *Mol. Ecol. Resour.* **16**, 459–469 (2016).
72. Meyer, M. & Kircher, M. Illumina sequencing library preparation for highly multiplexed target capture and sequencing. *Cold Spring Harb. Protoc.* **2010**, db.prot5448 (2010).
73. Boessenkool, S. *et al.* Combining bleach and mild predigestion improves ancient DNA recovery from bones. *Mol. Ecol. Resour.* **17**, 742–751 (2017).
74. Kemp, B. M. & Smith, D. G. Use of bleach to eliminate contaminating DNA from the surface of bones and teeth. *Forensic Sci. Int.* **154**, 53–61 (2005).
75. Rohland, N., Glocke, I., Aximu-Petri, A. & Meyer, M. Extraction of highly degraded DNA from ancient bones, teeth and sediments for high-throughput sequencing. *Nat. Protoc.* **13**, 2447–2461 (2018).
76. Prendergast, M. E. *et al.* Ancient DNA reveals a multistep spread of the first herders into sub-Saharan Africa. *Science* **365**, eaaw6275 (2019).
77. Carøe, C. *et al.* Single-tube library preparation for degraded DNA. *Methods Ecol. Evol.* **9**, 410–419 (2018).
78. Fellows Yates, J. A. *et al.* Reproducible, portable, and efficient ancient genome reconstruction with nf-core/eager. *PeerJ* **9**, e10947 (2021).
79. Okonechnikov, K., Conesa, A. & García-Alcalde, F. Qualimap 2: advanced multi-sample quality control for high-throughput sequencing data. *Bioinformatics* **32**,

292–294 (2016).
80. Bougiouri, K. *et al.* Imputation of ancient canid genomes reveals inbreeding history over the past 10,000 years. *bioRxiv* (2024) doi:10.1101/2024.03.15.585179.
81. Danecek, P. *et al.* Twelve years of SAMtools and BCFtools. *Gigascience* **10**, (2021).
82. Li, H. *et al.* The Sequence Alignment/Map format and SAMtools. *Bioinformatics* **25**, 2078–2079 (2009).
83. Katoh, K. & Standley, D. M. MAFFT multiple sequence alignment software version 7: improvements in performance and usability. *Mol. Biol. Evol.* **30**, 772–780 (2013).
84. Nguyen, L.-T., Schmidt, H. A., von Haeseler, A. & Minh, B. Q. IQ-TREE: A Fast and Effective Stochastic Algorithm for Estimating Maximum-Likelihood Phylogenies. *Mol. Biol. Evol.* **32**, 268–274 (2015).
85. Kalyaanamoorthy, S., Minh, B. Q., Wong, T. K. F., von Haeseler, A. & Jermiin, L. S. ModelFinder: fast model selection for accurate phylogenetic estimates. *Nat. Methods* **14**, 587–589 (2017).
86. Bouckaert, R. *et al.* BEAST 2: a software platform for Bayesian evolutionary analysis. *PLoS Comput. Biol.* **10**, e1003537 (2014).
87. Taming the BEAST - A community teaching material resource for BEAST 2. *Systematic Biology*.
88. Rambaut, A., Drummond, A. J., Xie, D., Baele, G. & Suchard, M. A. Posterior Summarization in Bayesian Phylogenetics Using Tracer 1.7. *Syst. Biol.* **67**, 901–904 (2018).
89. Patterson, N., Price, A. L. & Reich, D. Population structure and eigenanalysis. *PLoS Genet.* **2**, e190 (2006).
90. Meisner, J., Liu, S., Huang, M. & Albrechtsen, A. Large-scale inference of population structure in presence of missingness using PCA. *Bioinformatics* **37**, 1868–1875 (2021).
91. Librado, P. & Orlando, L. Struct-f4: a Rcpp package for ancestry profile and population structure inference from f4-statistics. *Bioinformatics* **38**, 2070–2071 (2022).
92. Lichstein, J. W. Multiple regression on distance matrices: a multivariate spatial analysis tool. *Plant Ecol.* **188**, 117–131 (2007).
93. Alexander, D. H., Novembre, J. & Lange, K. Fast model-based estimation of ancestry in unrelated individuals. *Genome Res.* **19**, 1655–1664 (2009).
94. Alaçamlı, E. *et al.* READv2: advanced and user-friendly detection of biological relatedness in archaeogenomics. *Genome Biol.* **25**, 216 (2024).
95. Monroy Kuhn, J. M., Jakobsson, M. & Günther, T. Estimating genetic kin relationships in prehistoric populations. *PLoS One* **13**, e0195491 (2018).
96. Mallick, S. *et al.* The Allen Ancient DNA Resource (AADR) a curated compendium of ancient human genomes. *Sci. Data* **11**, 182 (2024).
97. Bergström, A. *et al.* Origins and genetic legacy of prehistoric dogs. *Science* **370**, 557–564 (2020).
98. Patterson, N. *et al.* Ancient admixture in human history. *Genetics* **192**, 1065–1093 (2012).
99. Bergström, A. *et al.* Grey wolf genomic history reveals a dual ancestry of dogs. *Nature* **607**, 313–320 (2022).
100. Pickrell, J. K. & Pritchard, J. K. Inference of population splits and mixtures from genome-wide allele frequency data. *PLoS Genet.* **8**, e1002967 (2012).
101. Nielsen, S. V. *et al.* Bayesian inference of admixture graphs on Native American and Arctic populations. *PLoS Genet.* **19**, e1010410 (2023).

102. Harney, É., Patterson, N., Reich, D. & Wakeley, J. Assessing the performance of qpAdm: a statistical tool for studying population admixture. *Genetics* **217**, iyaa045 (2021).
103. Flegontova, O. *et al.* Performance of qpAdm-based screens for genetic admixture on graph-shaped histories and stepping stone landscapes. *Genetics* **230**, iyaf047 (2025).
104. Mallick, S. & Reich, D. The Allen Ancient DNA Resource (AADR): A curated compendium of ancient human genomes. Harvard Dataverse https://doi.org/10.7910/DVN/FFIDCW (2023).
105. Wood, D. E., Lu, J. & Langmead, B. Improved metagenomic analysis with Kraken 2. *Genome Biol.* **20**, 257 (2019).
