# Supplementary material for: Dogs were widely distributed across western Eurasia during the Palaeolithic
Source: Nature. 2026 Mar 25;651(8107):995–1003. doi: 10.1038/s41586-026-10170-x (PMC13017512; doi:10.1038/s41586-026-10170-x)
Supplement: Supplementary file 2 — Reporting Summary [file 41586_2026_10170_MOESM2_ESM.pdf]

nature portfolio

Corresponding author(s): William Marsh, Lachie Scarsbrook, Ian Barnes, Greger Larson, Laurent Frantz

Last updated by author(s): 16/01/2026

Reporting Summary

Nature Portfolio wishes to improve the reproducibility of the work that we publish. This form provides structure for consistency and transparency in reporting. For further information on Nature Portfolio policies, see our Editorial Policies and the Editorial Policy Checklist.

Statistics

For all statistical analyses, confirm that the following items are present in the figure legend, table legend, main text, or Methods section.

n/a	Confirmed	
☐	☒	The exact sample size (n) for each experimental group/condition, given as a discrete number and unit of measurement
☐	☒	A statement on whether measurements were taken from distinct samples or whether the same sample was measured repeatedly
☐	☒	The statistical test(s) used AND whether they are one- or two-sided<br>*Only common tests should be described solely by name; describe more complex techniques in the Methods section.*
☐	☒	A description of all covariates tested
☐	☒	A description of any assumptions or corrections, such as tests of normality and adjustment for multiple comparisons
☐	☒	A full description of the statistical parameters including central tendency (e.g. means) or other basic estimates (e.g. regression coefficient) AND variation (e.g. standard deviation) or associated estimates of uncertainty (e.g. confidence intervals)
☐	☒	For null hypothesis testing, the test statistic (e.g. F, t, r) with confidence intervals, effect sizes, degrees of freedom and P value noted<br>*Give P values as exact values whenever suitable.*
☐	☒	For Bayesian analysis, information on the choice of priors and Markov chain Monte Carlo settings
☒	☐	For hierarchical and complex designs, identification of the appropriate level for tests and full reporting of outcomes
☐	☒	Estimates of effect sizes (e.g. Cohen's d, Pearson's r), indicating how they were calculated

Our web collection on statistics for biologists contains articles on many of the points above.

Software and code

Policy information about availability of computer code

Data collection	*Provide a description of all commercial, open source and custom code used to collect the data in this study, specifying the version used OR state that no software was used.*
Data analysis	Radiocarbon Analyis: Oxcal v.4.4<br>Isotopic Analysis: R Studio<br>Ancient DNA Analysis: nf-core/eager v.2.4.6, CanID v1.0, bcftools, ANSGD, samtools v.1.16.1, MAFFT v7.505, IQ-TREE v.2.1.4, BEAST2 v.2.6.7, Tracer v.2.6.7, LogCombiner v.2.6.7, EIGENSOFT v.8.0.0, EMU v.0.9, struct-f4, ADMIXTURE v.1.3.0, READv2, GLIMPSE v1.1.1, PLINK v.1.90b6.21, AdmixTools v.7.0.2, TreeMix v.1.13, AdmixtureBayes, R Studio

For manuscripts utilizing custom algorithms or software that are central to the research but not yet described in published literature, software must be made available to editors and reviewers. We strongly encourage code deposition in a community repository (e.g. GitHub). See the Nature Portfolio guidelines for submitting code & software for further information.

Data

Policy information about availability of data

All manuscripts must include a data availability statement. This statement should provide the following information, where applicable:

- Accession codes, unique identifiers, or web links for publicly available datasets
- A description of any restrictions on data availability
- For clinical datasets or third party data, please ensure that the statement adheres to our policy

All newly generated ancient sequences (SAMEA120632851–SAMEA120632875) are available in the ENA under BioProject PRJEB104454 (https://www.ebi.ac.uk/ena/browser/view/PRJEB104454). Raw radiocarbon age estimates are reported in Supplementary Dataset 2. Raw isotopic measurements are reported in Supplementary Dataset 6. All other data are included in the manuscript and/or supporting information.

Research involving human participants, their data, or biological material

Policy information about studies with human participants or human data. See also policy information about sex, gender (identity/presentation), and sexual orientation and race, ethnicity and racism.

Reporting on sex and gender	*Use the terms sex (biological attribute) and gender (shaped by social and cultural circumstances) carefully in order to avoid confusing both terms. Indicate if findings apply to only one sex or gender; describe whether sex and gender were considered in study design; whether sex and/or gender was determined based on self-reporting or assigned and methods used. Provide in the source data disaggregated sex and gender data, where this information has been collected, and if consent has been obtained for sharing of individual-level data; provide overall numbers in this Reporting Summary. Please state if this information has not been collected. Report sex- and gender-based analyses where performed, justify reasons for lack of sex- and gender-based analysis.*
Reporting on race, ethnicity, or other socially relevant groupings	*Please specify the socially constructed or socially relevant categorization variable(s) used in your manuscript and explain why they were used. Please note that such variables should not be used as proxies for other socially constructed/relevant variables (for example, race or ethnicity should not be used as a proxy for socioeconomic status). Provide clear definitions of the relevant terms used, how they were provided (by the participants/respondents, the researchers, or third parties), and the method(s) used to classify people into the different categories (e.g. self-report, census or administrative data, social media data, etc.) Please provide details about how you controlled for confounding variables in your analyses.*
Population characteristics	*Describe the covariate-relevant population characteristics of the human research participants (e.g. age, genotypic information, past and current diagnosis and treatment categories). If you filled out the behavioural & social sciences study design questions and have nothing to add here, write "See above."*
Recruitment	*Describe how participants were recruited. Outline any potential self-selection bias or other biases that may be present and how these are likely to impact results.*
Ethics oversight	*Identify the organization(s) that approved the study protocol.*

Note that full information on the approval of the study protocol must also be provided in the manuscript.

Field-specific reporting

Please select the one below that is the best fit for your research. If you are not sure, read the appropriate sections before making your selection.

☐ Life sciences ☐ Behavioural & social sciences ☒ Ecological, evolutionary & environmental sciences

For a reference copy of the document with all sections, see nature.com/documents/nr-reporting-summary-flat.pdf

Ecological, evolutionary & environmental sciences study design

All studies must disclose on these points even when the disclosure is negative.

Study description	Analysis of suspected Palaeolithic and Mesolithic dog genomes from across Western Eurasia.
Research sample	Ancient dog (Canis lupus familiaris; n = 22) and wolf (Canis lupus lupus; n = 5) remains were sampled from across Western Eurasia including the UK, Serbia, Italy, Türkiye and Iran. Detailed information including site and specimen descriptions can be found in the Supplementary Methods.
Sampling strategy	All individuals which possessed enough bone/tooth material were sampled for this study (see Supplementary Methods). Material was obtained through collaboration with archaeologists working in the respective regions (see author list).
Data collection	DNA was extracted, and double-stranded dual-indexed libraries were prepared from samples at either: Ancient DNA Laboratory, Natural History Museum, London; Department of Veterinary Medicine, Ludwig Maximilian University, Munich; or Research Lab for

	Archaeology and the History of Art, University of Oxford, Oxford.
Timing and spatial scale	Samples ranged in age from 1532 to 15837 years calBP.
Data exclusions	No data was excluded from the analysis.
Reproducibility	Detailed description of all experimental work is available in the supplementary information. All institutional/archaeological codes from which samples were taken are also detailed. No-template ("blank") controls were processed alongside all samples. These samples failed to amplify efficiently and no reads sequenced from no-template libraries could be unambiguously mapped, indicating that background and cross-contamination were minimal.
Randomization	No experiment requiring randomization was conducted in this study.
Blinding	No experiment requiring blinding was conducted in this study.

Did the study involve field work? ☐ Yes ☒ No

Reporting for specific materials, systems and methods

We require information from authors about some types of materials, experimental systems and methods used in many studies. Here, indicate whether each material, system or method listed is relevant to your study. If you are not sure if a list item applies to your research, read the appropriate section before selecting a response.

Materials & experimental systems

n/a	Involved in the study	
☒	☐	Antibodies
☒	☐	Eukaryotic cell lines
☐	☒	Palaeontology and archaeology
☒	☐	Animals and other organisms
☒	☐	Clinical data
☒	☐	Dual use research of concern
☒	☐	Plants

Methods

n/a	Involved in the study	
☒	☐	ChIP-seq
☒	☐	Flow cytometry
☒	☐	MRI-based neuroimaging

Palaeontology and Archaeology

Specimen provenance	M13794, Goughs Cave, UK (NHM)<br>M49877, Goughs Cave, UK (NHM)<br>M13795, Goughs Cave, UK (NHM)<br>M50014, Goughs Cave, UK (NHM)<br>M50015, Goughs Cave, UK (NHM)<br>M13796, Goughs Cave, UK (NHM)<br>M50013, Goughs Cave, UK (NHM)<br>AL2934, Grotta Continenza, Italy (Oxford)<br>OL4117, Padina, Serbia (Oxford)<br>OL4090 / SC1178, Padina, Serbia (Oxford)<br>OL4109 / SC1182, Padina, Serbia (Oxford)<br>OL4118 / LS0288, Padina, Serbia (Oxford)<br>OL4098 / SC1180, Padina, Serbia (Oxford)<br>D004924, Pinarbasi, Türkiye (LMU)<br>D004924, Pinarbasi, Türkiye (LMU)<br>D004924, Pinarbasi, Türkiye (LMU)<br>AL2921/ D004922, Pinarbasi, Türkiye (LMU)<br>AL2918 / D004912, Pinarbasi, Türkiye (LMU)<br>AL2919 / D004913, Pinarbasi, Türkiye (LMU)<br>AL2884, Vlasac, Serbia (Oxford)<br>OL4071, Vlasac, Serbia (Oxford)<br>OL4073 / SC1188, Vlasac, Serbia (Oxford)<br>WZ77, Wezmeh, Iran (LMU)<br>WZ189, Wezmeh, Iran (LMU)<br>WZ194, Wezmeh, Iran (LMU)<br>WZ190, Wezmeh, Iran (LMU)
Specimen deposition	Specimens from: Gough's Cave are stored at the Natural History Museum of London (Simon A Parfitt, Silvia M Bello); Wezmeh Cave are stored at Muséum National d'Histoire Naturelle (Marjan Mashkour), Paris; Pinarbasi are stored at University of Liverpool, UK (Douglas Baird, Louise Martin); and Serbia are stored at University of Belgrade - Faculty of Philosophy, Serbia (Vesna Dimitrijevic,

Sonja Vukovic).

Dating methods

Collagen extraction and AMS dating was conducted at either the Oxford Radiocarbon Accelerator Unit (Oxford, UK), or the Radiocarbon laboratory of the University of Vienna (Vienna, Austria). Mean age and 95% confidence-intervals were estimated from raw dates (see Supplementary Methods) using the IntCal20 calibration correction in Oxcal v.4.4.

☒ Tick this box to confirm that the raw and calibrated dates are available in the paper or in Supplementary Information.

Ethics oversight

No ethical approval or guidance was required in this study.

Note that full information on the approval of the study protocol must also be provided in the manuscript.

Plants

Seed stocks

Report on the source of all seed stocks or other plant material used. If applicable, state the seed stock centre and catalogue number. If plant specimens were collected from the field, describe the collection location, date and sampling procedures.

Novel plant genotypes

Describe the methods by which all novel plant genotypes were produced. This includes those generated by transgenic approaches, gene editing, chemical/radiation-based mutagenesis and hybridization. For transgenic lines, describe the transformation method, the number of independent lines analyzed and the generation upon which experiments were performed. For gene-edited lines, describe the editor used, the endogenous sequence targeted for editing, the targeting guide RNA sequence (if applicable) and how the editor was applied.

Authentication

Describe any authentication procedures for each seed stock used or novel genotype generated. Describe any experiments used to assess the effect of a mutation and, where applicable, how potential secondary effects (e.g. second site T-DNA insertions, mosiacism, off-target gene editing) were examined.
