# Supplementary material for: Dogs were widely distributed across western Eurasia during the Palaeolithic
Source: Nature. 2026 Mar 25;651(8107):995–1003. doi: 10.1038/s41586-026-10170-x (PMC13017512; doi:10.1038/s41586-026-10170-x)
Supplement: Supplementary file 3 — Supplementary Tables 1–7. [file 41586_2026_10170_MOESM3_ESM.zip › 2025-05-12885B-s3_WM_Updated/supplementary-table-legends.docx]

**Supplementary Table 1**. Ancient nuclear genome metadata. Metadata for all new and publicly available ancient dogs and wolves used in this study (which includes archaeological ID and material, locality information, and ENA accession numbers). Radiocarbon dates were calibrated using the IntCal20 calibration curve (as all samples were from the Northern Hemisphere).

**Supplementary Table 2**. Radiocarbon dates for suspected Palaeolithic canids, and publicly-available data from humans at the same sites. Dates were calibrated using the IntCal20 calibration curve (as all samples were from the Northern Hemisphere).

**Supplementary Table 3**. Modern nuclear genome metadata. ENA accession numbers, and breed/population or locality information for publicly available dogs and wolves used in this study.

**Supplementary Table 4**. Ancient mitochondrial genome metadata. Metadata for individuals included in mitochondrial genome analysis including taxonomic status (dog or wolf) based on CanID, and breadth/depth statistics.

**Supplementary Table 5**. Ancient and modern mitochondrial genome metadata. Metadata for individuals used in maximum-likelihood and Bayesian phylogenies.

**Supplementary Table 6**. Compound-specific Isotope Analysis (CSIA) metadata for ancient dogs and humans. Results of novel CSIA performed on Gough’s Cave and Pinarbasi canid and human specimens. Analysis performed at the University of York. Data authenticated using observed vs expected bulk isotope values and observed Hydroxyproline:Proline *δ*^15^N and *δ*^13^C amino acid ratios.

**Supplementary Table 7**. Metadata for paired ancient dog and human populations. This includes ENA accession codes for human nuclear genome data (ENA accession codes for dogs can be found in Supplementary Table 1), which were retrieved from the open-access Allen Ancient DNA Resource (AADR).
